# Antioxidants as Molecular Probes: Structurally Novel Dihydro-*m*-Terphenyls as Turn-On Fluorescence Chemodosimeters for Biologically Relevant Oxidants

**DOI:** 10.3390/antiox9070605

**Published:** 2020-07-10

**Authors:** Víctor González-Ruiz, Jegathalaprathaban Rajesh, Ana I. Olives, Damiano Rocchi, Jorge Gómez-Carpintero, Juan F. González, Vellaisamy Sridharan, M. Antonia Martín, J. Carlos Menéndez

**Affiliations:** 1 Unidad de Química Analítica, Departamento de Química en Ciencias Farmacéuticas, Facultad de Farmacia, Universidad Complutense, 28040 Madrid, Spain; victor.gonzalez@unige.ch (V.G.-R.); mkuraji@gmail.com (J.R.); aiolives@farm.ucm.es (A.I.O.); 2 Unidad de Química Orgánica y Farmacéutica, Departamento de Química en Ciencias Farmacéuticas, Facultad de Farmacia, Universidad Complutense, 28040 Madrid, Spain; rocchid83@gmail.com (D.R.); jgomez21892@gmail.com (J.G.-C.); jfgonzal@ucm.es (J.F.G.); 3 Department of Chemistry and Chemical Sciences, Central University of Jammu, Rahya-Suchani (Bagla), District-Samba, Jammu-181143, J&K, India; vesridharan@gmail.com

**Keywords:** reactive oxygen species, dihydroterphenyl antioxidants, fluorescence probes, inner charge transfer, glucose quantitation

## Abstract

One interesting aspect of antioxidant organic molecules is their use as probes for the detection and quantitation of biologically relevant reactive oxidant species (ROS). In this context, a small library of dihydroterphenyl derivatives has been synthesised and studied as fluorescent chemodosimeters for detecting reactive oxygen species and hypochlorite. The fluorescence quantum yields of these molecules are negligible, while the corresponding aromatized compounds formed upon oxidation show moderate to high native fluorescence, depending on their structures. The fluorescence signal is quickly developed in the presence of trace amounts of the probe and the analytes in acetonitrile media at room temperature, with good analytical figures. ROS detection in aqueous media required incubation at 37 °C in the presence of horseradish peroxidase, and was applied to glucose quantitation by coupling glucose oxidation by O_2_ to fluorescence detection of H_2_O_2_. The mild reaction conditions and sensitive fluorescent response lead us to propose dihydroterphenyls with an embedded anthranilate moiety as chemosensors/chemodosimeters for ROS detection.

## 1. Introduction

Reactive oxygen species (ROS) [1] exert important physiological roles, being involved in processes such as cell signaling and immune responses [2,3], but on the other hand, their buildup in cells is linked to the mechanisms leading to a number of pathological conditions including cardiovascular disorders [4,5], cancer [6,7] and neurodegenerative diseases [8,9]. Hydrogen peroxide is a product of the natural aerobic metabolism in cells, but its overproduction can be related to alterations in the mitochondrial electron transport chain, and high levels of mitochondrial hydrogen peroxide are a contributing factor in the pathogenesis of Parkinson’s [10] and Alzheimer’s [11] diseases. Other biological hydroperoxides such as prostaglandin G2 [12] or protein hydroperoxides [13] are also important agents of oxidative stress. Another significant ROS is hypochlorite, which is synthesized in living organisms from hydrogen peroxide and chloride ions, mainly inside leukocytes, in a reaction catalyzed by the enzyme myeloperoxidase (MPO) [14]. Hypochlorite can react with a variety of biomolecules, causing damage to biological systems, and, therefore, the interest in hypochlorous acid sensors has risen in recent years [15,16,17].

Synthetic probes able to respond to the variation of ROS concentrations are of great interest as diagnostic agents. Due to the sensitivity of fluorimetric detection, fluorescence sensors and chemodosimeters are particularly attractive, because they offer valuable qualitative and quantitative information without the need for expensive equipment. Thus, there is a growing interest in fluorescence spectroscopy as a non-invasive diagnostic tool for real-time visualization of biomolecules in living systems [18,19,20,21]. Several characteristics are desirable for a fluorescent dye with sensing properties [22,23,24,25], namely: (a) an adequate hydrophobicity/hydrophilicity balance to ensure both membrane permeability and solubility in biological media; (b) biocompatibility, with no interference with endogenous cellular processes; (c) good analytical features: sensitivity, selectivity and linear range; (d) a high fluorescence emission intensity, preferably only upon reaction with the target analyte (i.e., turn-on behaviour); (e) photo-stability; (f) in the case of sensors to be employed for bioimaging, emission in the red or near infrared is an additional useful feature.

Due to the nature of the fluorescence emission phenomena, several excited state relaxation processes can be exploited to connect the ground and excited states, events that are always reversible and therefore ideal for analyte sensing. Among them, photoinduced electron transfer PET) has been successfully employed for the detection of HClO in several environments [26,27]. Intra-molecular charge transfer ICT) is observed in organic dyes with conjugated electron-donating and electron-withdrawing groups placed at opposite ends of the molecule (push-pull systems), with a high level of electronic conjugation. Among other examples, the reduction to amine of an azide group remotely attached to a 1,8-naphthalimide framework via a benzyloxycarbonylamino spacer in the presence of Na_2_S causes the cleavage of the spacer, leading to a change of green to blue fluorescence via an ICT event. A good linear relationship between the emission intensity ratios and sulphide concentrations was observed in PBS buffer and bovine serum, and the method also allowed the ratiometric measurement of sulphide levels in mouse hippocampus [28]. Förster resonance energy transfer FRET) is a process frequently produced in organic dyes, where donor and acceptor moieties may exchange their excited-state energies, in an event that causes a fluorescence enhancement. For instance, FRET from coumarin to rhodamine moieties is observed in a novel probe after reaction with HClO, providing imaging of endogenous HClO in living cells [29]. In some cases, the sensor response involves two mechanisms, namely PET and excited state proton transfer ESPT) acting simultaneously, facilitating the detection of several peroxides in alkaline media [30]. The interaction between dyes and macromolecules (or between dyes and nanoparticles) can produce aggregates, with a concomitant fluorescence enhancement (aggregation-induced emission (AIE)), which has been exploited for detecting ROS [31,32] and also other species of biological interest [33].

**Scheme 1:**
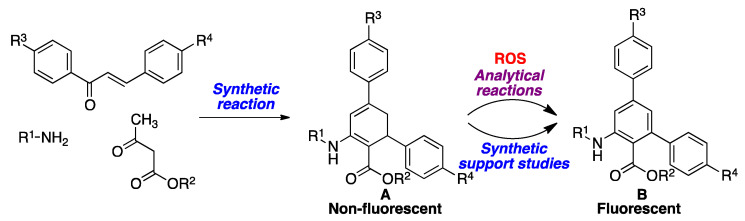
A general scheme of the reactive oxidant species (ROS) sensing reaction presented in this article.

Many of the known ROS probes have the disadvantage of being fluorescent by themselves, which poses practical problems associated to the measurement of an emission signal drop against a bright background. This shortcoming does not exist in turn-on fluorescent probes, i.e., compounds that have very low or no native fluorescence, but are transformed into a fluorescent species after undergoing a chemical reaction induced by the analyte. In this context, non-fluorescent organic compounds that become aromatic in the presence of oxidizing reagents such as ROS, are of interest as potential chemosensors/chemodosimeters. Most known redox fluorescence sensors of this type belong to a few structural classes containing well-established chromophores, in particular, chromone precursors, dichlorofluorescein and its derivatives, hydroethidine, dihydrorhodamine, and boronates, which are probably due to limited synthetic accessibility. As a result of the interesting fluorescence properties of both anthranilate esters [34] and *m*-terphenyl derivatives [35,36,37], it can be inferred that dihydroterphenyls, with a latent anthranilate moiety, would be ideal candidates for the desired turn-on behavior upon exposure to ROS, since they should be easily dehydrogenated to the corresponding aromatized derivatives, rendering them highly fluorescent through an efficient ICT process. Furthermore, the amino and ester groups have the potential to be employed as synthetic handles for further functionalization processes, e.g., with polymers. Such dihydroterphenyl sensors would be synthesized by application of a three-component reaction developed by our group [38], and starting from chalcones, primary amines, and β-ketoesters (Scheme 1). As a panel of model biologically relevant ROS species, hydrogen peroxide was chosen because of its well-known importance in oxidative stress; hypochlorite, which has been much less studied from a sensor design perspective; and *tert*-butyl hydroperoxide as a model of the biological hydroperoxides. 

## 2. Materials and Methods

### 2.1. General Experimental Information

All reagents (Aldrich, Fluka, SDS, Probus) and solvents (SDS) were of commercial quality and were used as received. Reactions were monitored by thin layer chromatography, on aluminum plates coated with silica gel with fluorescent indicator (SDS CCM221254). Separations by flash chromatography were performed on silica gel (SDS 60 ACC 15 40–63 μm) or neutral alumina (Merck S22). Melting points were measured on a Reichert 723 hot stage microscope, and are uncorrected. Infrared spectra were recorded on a Perkin Elmer Paragon 1000 FT-IR spectrophotometer (Perkin Elmer España, Tres Cantos, Spain), with all compounds examined as KBr pellets or as thin films on NaCl 20 disks. NMR spectra were obtained on a Bruker Avance 250 spectrometer working at 250 MHz for ^1^H and 63 MHz for ^13^C and operated via the standard Bruker software (CAI de Resonancia Magnética Nuclear, Universidad Complutense, Madrid, Spain). Elemental analyses were performed by the CAI de Microanálisis Elemental, Universidad Complutense, using a Leco 932 CHNS combustion microanalyzer. For the fluorescence studies, all reagents and solvents employed were analytical grade and ultrapure water was obtained from a Milli-Q Direct 8 system (Millipore). Ultraviolet-visible absorption spectra were recorded with an automatic double beam Uvikon 810 spectrophotometer (Kontron, Augsburg, Germany). Corrected excitation and emission fluorescence spectra, as well as fluorescence emission at fixed wavelengths, were carried out with a FluoroMax-4P spectrofluorometer equipped with the control and data acquisition software FluorEssence 2.1 (Horiba-Jobin Yvon, Kyoto, Japan). Measurements at fixed and variable wavelengths were carried out on a MicroMax 384 multi-well plate reader coupled to a FluoroMax-4P spectrofluorometer (both from Horiba-Jobin Yvon, Kyoto, Japan) using excitation and emission slits of 5 nm band pass, quartz cells of 1 cm path length and on black 96-well micro-plates.

### 2.2. Synthesis

#### 2.2.1. Synthesis of Compounds **A**

To a stirred solution of ethyl acetoacetate (3 mmol, 1 eq) and the suitable primary amine (3 to 3.9 mmol, 1 to 1.3 eq) in ethanol (5 mL) was added Cerium(IV) ammonium nitrate (CAN) (5 mol%). Stirring was continued for 30 min at room temperature. Chalcone (2.4 to 3.6 mmol, 0.8 to 1.2 eq) was then added to the stirred solution and the mixture was heated under reflux for 8 h (compounds **A1**–**A7**) or 20 h (compounds **A8–A10**). After completion of the reaction, as indicated by thin layer chromatography (TLC), the mixture was dissolved in ether (30 mL), washed successively with water and brine and dried (anhydrous Na_2_SO_4_). The solvent was evaporated under reduced pressure and pure compounds **A** were purified by flash column chromatography on silica gel, eluting with a petroleum ether-ethyl acetate mixture (96:4, v/v). Compounds **A1**, **A6**, **A7** and **A8** were known in the literature, and their characterization data were identical to those previously described [38]. Data for new compounds are given below.

Ethyl 2-(butylamino)-4-(4-chlorophenyl)-6-phenylcyclohexa-1,3-dienecarboxylate (**A2**). Mp 88–89 °C. IR (neat) 3263.8, 2957.1, 2868.7, 1646.3, 1574.3, 1448.6, 1215.2, 1078.1 cm^−1^. ^1^H-NMR (CDCl_3_, 250 MHz) δ 0.99 (t, *J* = 7.2 Hz, 3H), 1.17 (t, *J* = 7.1 Hz, 3H), 1.44-1.56 (m, 2H), 1.61–1.71 (m, 2H), 2.88 (dd, *J* = 16.7, 1.3 Hz, 1H), 3.14 (ddd, *J* = 16.7, 8.3, 2.7 Hz, 1H), 3.34-3.45 (m, 2H), 4.00–4.16 (m, 2H), 4.27 (dd, *J* = 8.3, 1.3 Hz, 1H), 6.62 (d, *J* = 2.7 Hz, 1H), 7.13–7.34 (m, 9H), 9.03 (bt, *J* = 4.8 Hz, 1H). ^13^C-NMR (CDCl_3_, 62.9 MHz) δ 14.3, 14.9, 20.6, 33.1, 35.5, 37.0, 43.1, 59.2, 89.9, 117.0, 126.3, 127.5, 127.6, 128.3, 129.1, 134.8, 139.1, 145.3, 145.9, 155.2, 170.8. Analysis. Calcd for C_25_H_28_ClNO_2_: C, 73.25; H, 6.88; N, 3.42. Found: C, 73.48; H, 6.73; N, 3.71.

Ethyl 2-(butylamino)-6-(4-methylphenyl)-4-phenylcyclohexa-1,3-dienecarboxylate (**A3**). Viscous oil. IR (neat) 3264.1, 2956.5, 2866.7, 1646.0, 1578.7, 1439.2, 1216.0, 1079.0 cm^−1^. ^1^H-NMR (CDCl_3_, 250 MHz) δ 1.00 (t, *J* = 7.2 Hz, 3H), 1.19 (t, *J* = 7.1 Hz, 3H), 1.42-1.56 (m, 2H), 1.65–1.73 (m, 2H), 2.28 (s, 3H), 2.94 (dd, *J* = 16.7, 1.3 Hz, 1H), 3.15 (ddd, *J* = 16.7, 8.3, 2.8 Hz, 1H), 3.35–3.46 (m, 2H), 4.00–4.18 (m, 2H), 4.25 (dd, *J* = 8.3, 1.3 Hz, 1H), 6.64 (d, *J* = 2.8 Hz, 1H), 7.02 (d, *J* = 8.5 Hz, 2H), 7.17 (d, *J* = 8.5 Hz, 2H), 7.29–7.38 (m, 5H), 9.05 (bt, *J* = 5.0 Hz, 1H). ^13^C-NMR (CDCl_3_, 62.9 MHz)* δ 14.3, 14.9, 20.6, 21.4, 33.1, 35.5, 36.6, 43.1, 59.1, 89.8, 116.6, 126.3, 127.5, 128.9, 129.0, 135.5, 140.8, 143.2, 146.8, 155.4, 170.9 (one aromatic carbon signal is merged with others). Anal. Calcd for C_26_H_31_NO_2_: C, 80.17; H, 8.02; N, 3.60. Found: C, 79.41; H, 7.75; N, 3.70.

Ethyl 2-(butylamino)-6-(4-methoxyphenyl)-4-phenylcyclohexa-1,3-dienecarboxylate (**A4**). Viscous oil. IR (neat) 3271.3, 2956.6, 2930.6, 1645.3, 1574.5, 1508.5, 1440.0, 1245.0, 1216.1, 1078.9 cm^−1^. ^1^H-NMR (CDCl_3_, 250 MHz) δ 1.00 (t, *J* = 7.3 Hz, 3H), 1.19 (t, *J* = 7.1 Hz, 3H), 1.42-1.56 (m, 2H), 1.62–1.74 (m, 2H), 2.93 (dd, *J* = 16.8, 1.7 Hz, 1H), 3.14 (ddd, *J* = 16.8, 8.2, 2.8 Hz, 1H), 3.33–3.51 (m, 2H), 3.77 (s, 3H), 3.98-4.18 (m, 2H), 4.24 (dd, *J* = 8.2, 1.7 Hz, 1H), 6.65 (d, *J* = 2.8 Hz, 1H), 6.76 (d, *J* = 8.7 Hz, 2H), 7.19 (d, *J* = 8.7 Hz, 2H), 7.31–7.42 (m, 5H), 9.04 (bt, *J* = 4.6 Hz, 1H). ^13^C-NMR (CDCl_3_, 62.9 MHz)* δ 14.3, 14.9, 20.6, 33.1, 35.7, 36.2, 43.1, 55.5, 59.1, 90.0, 113.6, 116.6, 126.3, 128.6, 128.9, 138.3, 140.8, 146.7, 155.4, 158.0, 170.9. Anal. Calcd for C_26_H_31_NO_3_: C, 77.01; H, 7.71; N, 3.45. Found: C, 75.20; H, 7.31; N, 3.65.

*tert*-Butyl 2-(butylamino)-4,6-diphenylcyclohexa-1,3-dienecarboxylate (**A5**). Mp 88–89 °C. IR (neat) 3255.6, 2959.0, 2930.1, 1645.9, 1575.2, 1452.9, 1290.2, 1163.0, 1077.4 cm^−1^. ^1^H-NMR (CDCl_3_, 250 MHz) δ 0.99 (t, *J* = 7.1 Hz, 3H), 1.35 (s, 9H), 1.43-1.52 (m, 2H), 1.60-1.71 (m, 2H), 2.90 (dd, *J* = 16.8, 1.3 Hz, 1H), 3.16 (ddd, *J* = 16.8, 8.2, 2.8 Hz, 1H), 3.31–3.43 (m, 2H), 4.20 (dd, *J* = 8.2, 1.3 Hz, 1H), 6.64 (d, *J* = 2.8 Hz, 1H), 7.11–7.36 (m, 10H), 8.95 (bt, *J* = 4.8 Hz, 1H). ^13^C-NMR (CDCl_3_, 62.9 MHz) δ 14.4, 20.6, 28.9, 33.4, 35.4, 37.9, 43.2, 78.6, 91.6, 116.8, 126.0, 126.2, 127.7, 128.2, 128.8, 128.9, 140.9, 145.8, 146.7, 154.8, 170.9. Anal. Calcd for C_27_H_33_NO_2_: C, 80.36; H, 8.24; N, 3.47. Found: C, 80.12; H, 8.07; N, 3.59. 

Ethyl 2-(4-methylphenylamino)-4,6-diphenylcyclohexa-1,3-dienecarboxylate (**A9**). Mp 104–105 °C. IR (neat) 3247.9, 2976.9, 2924.9, 1646.2, 1567.9, 1514.3, 1449.8, 1230.4, 1077.3 cm^−1^. ^1^H-NMR (CDCl_3_, 250 MHz) δ 1.20 (t, *J* = 7.1 Hz, 3H), 2.38 (s, 3H), 3.01 (dd, *J* = 16.7, 1.6 Hz, 1H), 3.25 (ddd, *J* = 16.7, 8.5, 2.8 Hz, 1H), 4.06–4.22 (m, 2H), 4.38 (dd, *J* = 8.5, 1.6 Hz, 1H), 6.67 (d, *J* = 2.8 Hz, 1H), 7.06 (d, *J* = 8.3 Hz, 2H), 7.15–7.33 (m, 12H), 10.73 (bs, 1H). ^13^C-NMR (CDCl_3_, 62.9 MHz) δ *14.8, 2*1.3, 34.9, 37.2, 59.7, 94.6, 118.4, 123.9, 126.3, 126.4, 127.7, 128.4, 128.8, 128.9, 130.1, 134.1, 137.5, 140.3, 144.4, 145.6, 152.0, 170.6. Anal. Calcd for C_28_H_27_NO_2_: C, 82.12; H, 6.65; N, 3.42. Found: C, 82.49; H, 6.62; N, 3.66.

Ethyl 2-(4-methoxyphenylamino)-4,6-diphenylcyclohexa-1,3-dienecarboxylate (**A10**). Mp 139–140 °C. IR (neat) 3247.9, 2977.6, 2917.9, 1644.7, 1568.7, 1511.7, 1450.7, 1229.8, 1077.6 cm^−1^. 1H-NMR (CDCl3, 250 MHz) δ 1.20 (t, *J* = 7.1 Hz, 3H), 3.00 (dd, *J* = 16.7, 1.5 Hz, 1H), 3.25 (ddd, *J* = 16.7, 8.4, 2.8 Hz, 1H), 3.85 (s, 3H), 4.06–4.22 (m, 2H), 4.37 (dd, *J* = 8.4, 1.5 Hz, 1H), 6.58 (d, J = 2.8 Hz, 1H), 6.92 (d, *J* = 8.9 Hz, 2H), 7.12 (d, *J* = 8.9 Hz, 2H), 7.15–7.37 (m, 10H), 10.73 (bs, 1H). ^13^C-NMR (CDCl_3_, 62.9 MHz) δ 14.9, 35.0, 37.2, 55.9, 59.6, 93.7, 114.7, 118.2, 126.0, 126.2, 126.4, 127.7, 128.4, 128.8, 133.1, 140.3, 144.7, 145.7, 152.6, 157.1, 170.7. Anal. Calcd for C_28_H_27_NO_3_: C, 79.03; H, 6.40; N, 3.29. Found: C, 78.92; H, 6.30; N, 3.58.

#### 2.2.2. Synthesis of Compounds **B**

To a stirred solution of the suitable compound **A** (1 mmol) in benzene (5 mL) 2,3-dichloro-5,6-dicyano-1,4-benzoquinone (DDQ) (1.1 mmol) was added, and the mixture was stirred at room temperature for 1 h. After completion of the reaction, the mixture was diluted with ether, washed with water, dried (anhydrous Na_2_SO_4_), and evaporated. The aromatized products **B** were purified by flash silica gel column chromatography, eluting with a petroleum ether-ethyl acetate mixture (96:4 to 85:15, v/v). Compounds **B1**, **B6**, **B7** and **B8** were known in the literature, and their characterization data were identical to those previously described [38]. Data for new compounds are given below.

Ethyl 2-(butylamino)-4-(4-chlorophenyl)-6-phenylbenzoate (**B2**). Mp 85–86 °C. IR (neat) 3393.0, 2959.1, 2930.0, 1681.9, 1579.5, 1493.9, 1248.1, 1110.4, 1014.4 cm^−1^. ^1^H-NMR (CDCl_3_, 250 MHz) δ 0.72 (t, *J* = 7.1 Hz, 3H), 1.01 (t, *J* = 7.2 Hz, 3H), 1.52 (sextet, *J* = 7.2 Hz, 2H), 1.73 (quin, *J* = 7.2 Hz, 2H), 3.27 (q, *J* = 6.7 Hz, 2H), 3.91 (q, *J* = 7.1 Hz, 2H), 6.56 (bt, *J* = 4.8 Hz, 1H), 6.80–6.83 (m, 2H), 7.29–7.44 (m, 7H), 7.58 (d, *J* = 8.4 Hz, 2H). ^13^C-NMR (CDCl_3_, 62.9 MHz) δ 13.5, 14.4, 20.8, 31.7, 43.6, 60.8, 108.8, 112.4, 117.4, 127.2, 128.3, 128.4, 128.9, 129.3, 134.4, 139.8, 143.9, 144.1, 145.6, 150.1, 170.2. Anal. Calcd for C_25_H_26_ClNO_2_: C, 73.61; H, 6.42; N, 3.43. Found: C, 73.63; H, 6.34; N, 3.69.

Ethyl 2-(butylamino)-6-(4-methylphenyl)-4-phenylbenzoate (**B3**). Viscous oil. IR (neat) 3390.2, 2958.3, 2928.8, 1682.0, 1567.1, 1429.1, 1248.3, 1110.2, 1064.2 cm^−1^. ^1^H-NMR (CDCl_3_, 250 MHz) δ 0.77 (t, *J* = 7.2 Hz, 3H), 1.02 (t, *J* = 7.2 Hz, 3H), 1.53 (sextet, *J* = 7.0 Hz, 2H), 1.71 (quin, *J* = 7.0 Hz, 2H), 2.43 (s, 3H), 3.28 (q, *J* = 6.8 Hz, 2H), 3.94 (q, *J* = 7.2 Hz, 2H), 6.48 (bt, *J* = 4.4 Hz, 1H), 6.87 (d, *J* = 1.6 Hz, 1H), 6.88 (d, *J* = 1.6 Hz, 1H), 7.21 (d, *J* = 8.3 Hz, 2H), 7.27 (d, *J* = 8.3 Hz, 2H), 7.37-7.51 (m, 3H), 7.66 (dd, *J* = 8.3, 1.6 Hz, 2H). ^13^C-NMR (CDCl_3_, 62.9 MHz) δ 13.6, 14.4, 20.8, 21.6, 31.8, 43.6, 60.8, 108.9, 112.4, 117.8, 127.7, 128.2, 128.3, 128.9, 129.1, 136.8, 141.2, 141.5, 145.1, 145.3, 149.9, 170.5. Anal. Calcd for C_26_H_29_NO_2_: C, 80.59; H, 7.54; N, 3.61. Found: C, 79.62; H, 7.29; N, 3.73.

Ethyl 2-(butylamino)-6-(4-methoxyphenyl)-4-phenylbenzoate (**B4**). Viscous oil. IR (neat) 3388.2, 2957.8, 2931.9, 1682.0, 1565.7, 1517.1, 1245.6, 1110.7, 1033.6 cm^−1^. ^1^H-NMR (CDCl_3_, 250 MHz) δ 0.82 (t, *J* = 7.2 Hz, 3H), 1.02 (t, *J* = 7.2 Hz, 3H), 1.53 (sextet, *J* = 7.1 Hz, 2H), 1.71 (quin, *J* = 7.1 Hz, 2H), 3.28 (q, *J* = 6.8 Hz, 2H), 3.88 (s, 3H), 3.97 (q, *J* = 7.1 Hz, 2H), 6.43 (bt, *J* = 4.7 Hz, 1H), 6.86-6.87 (m, 2H), 6.95 (d, *J* = 8.5 Hz, 2H), 7.31 (d, *J* = 8.5 Hz, 2H), 7.40-7.50 (m, 3H), 7.66 (d, *J* = 7.4 Hz, 2H). ^13^C-NMR (CDCl_3_, 62.9 MHz) δ 13.8, 14.4, 20.8, 31.8, 43.6, 55.8, 60.8, 108.8, 112.5, 113.8, 117.8, 127.7, 128.3, 129.1, 129.5, 136.7, 141.5, 144.8, 145.1, 149.9, 159.1, 170.5. Anal. Calcd for C_26_H_29_NO_3_: C, 77.39; H, 7.24; N, 3.47. Found: C, 77.33; H, 7.28; N, 3.59.

*tert*-Butyl 2-(butylamino)-4,6-diphenylcyclohexa-1,3-dienecarboxylate (**B5**). Viscous oil. IR (neat) 3384.9, 2959.5, 2930.0, 1677.7, 1566.6, 1367.0, 1249.6, 1124.6 cm^−1^. ^1^H-NMR (CDCl_3_, 250 MHz) δ 1.03 (t, *J* = 7.2 Hz, 3H), 1.18 (s, 9H), 1.54 (sextet, *J* = 7.1 Hz, 2H), 1.76 (quint, *J* = 7.1 Hz, 2H), 3.30 (q, *J* = 6.8 Hz, 2H), 6.59 (bt, *J* = 4.4 Hz, 1H), 6.83 (d, *J* = 1.3 Hz, 1H), 6.91 (d, *J* = 1.3 Hz, 1H), 7.36-7.51 (m, 8H), 7.66 (dd, *J* = 8.3, 1.4 Hz, 2H). ^13^C-NMR (CDCl_3_, 62.9 MHz) δ 14.4, 20.8, 27.9, 31.8, 43.6, 81.5, 109.1, 113.8, 117.8, 127.0, 127.7, 128.2, 128.3, 128.9, 129.1, 141.6, 144.4, 144.7, 145.1, 149.9, 169.4. Anal. Calcd for C_27_H_31_NO: C, 80.76; H, 7.78; N, 3.49. Found: C, 80.95; H, 7.91; N, 3.39.

Ethyl 2-(4-methylphenylamino)-4,6-diphenylcyclohexa-1,3-dienecarboxylate (**B9**). Mp 127-128 °C. IR (neat) 3368.2, 2979.5, 2958.2, 1684.2, 1560.1, 1407.2, 1257.3, 1103.2 cm^−1^. ^1^H-NMR (CDCl_3_, 250 MHz) δ 0.79 (t, *J* = 7.2 Hz, 3H), 2.39 (s, 3H), 3.98 (q, *J* = 7.2 Hz, 2H), 7.07 (d, *J* = 1.7 Hz, 1H), 7.19-7.23 (m, 4H), 7.38-7.48 (m, 8H), 7.52 (d, *J* = 1.7 Hz, 1H), 7.59 (dd, *J* = 8.3, 1.7 Hz, 2H), 8.17 (bs, 1H). ^13^C-NMR (CDCl_3_, 62.9 MHz)* δ 13.6, 21.3, 61.2, 112.8, 115.9, 120.4, 121.9, 127.4, 127.7, 128.4, 128.5, 129.2, 130.5, 132.9, 139.2, 140.8, 143.5, 144.6, 145.0, 146.3, 170.1 (one aromatic carbon signal is merged with others). Anal. Calcd for C_28_H_25_NO_2_: C, 82.53; H, 6.18; N, 3.44. Found: C, 82.63; H, 6.24; N, 3.60.

Ethyl 2-(4-methoxyphenylamino)-4,6-diphenylcyclohexa-1,3-dienecarboxylate (**B10**). Mp 109–110 °C. IR (neat) 3366.8, 2980.1, 2904.7, 1682.8, 1561.4, 1512.9, 1407.7, 1241.9, 1102.8 cm^−1^. ^1^H-NMR (CDCl_3_, 250 MHz) δ 0.77 (t, *J* = 7.2 Hz, 3H), 3.86 (s, 3H), 3.97 (q, *J* = 7.2 Hz, 2H), 6.95 (d, *J* = 8.8 Hz, 2H), 7.01 (d, *J* = 1.7 Hz, 1H), 7.26 (d, *J* = 8.8 Hz, 2H), 7.30 (d, *J* = 1.7 Hz, 1H), 7.36-7.46 (m, 8H), 7.55 (dd, *J* = 8.2, 2.0 Hz, 2H), 8.12 (bs, 1H). ^13^C-NMR (CDCl_3_, 62.9 MHz) δ 13.6, 55.9, 61.6, 111.9, 114.7, 115.2, 119.8, 125.0, 127.3, 127.6, 128.3, 128.4, 128.5, 129.1, 134.6, 140.9, 143.7, 144.7, 145.2, 147.6, 156.6, 170.2. Anal. Calcd for C_28_H_25_NO_3_: C, 79.41; H, 5.95; N, 3.31. Found: C, 79.95; H, 5.99; N, 3.52.

### 2.3. Computational Studies

The geometrical parameters of compounds **B1** and **B8** and their electronic properties were calculated using MacSpartan 10 (Wavefunction Inc., Irvine, CA, USA). A conformational search was performed with the suitable Spartan tool at the semiempirical PM6 level, and the most stable conformer thus found was further optimized using the density-functional theory (DFT) approximation at the B3LYP//6-311 + G* level, which was also used for the molecular orbital calculations. 

#### 2.4.1. Native Fluorescence Studies

Some 1.0 × 10^−3^ M stock solutions in EtOH of compounds of the series **A** and **B** were prepared from accurately weighed amounts of each compound. Appropriate aliquots were taken from these solutions, evaporated in vacuo, and dissolved in the desired solvent (ethanol, acetonitrile, or cyclohexane) to a final 1.0 × 10^−6^ M concentration to record their fluorescence spectra. The fluorescence quantum yields (Φ) were determined from the corrected fluorescence spectra, integrated under the full fluorescence emission range and using quinine sulphate in 0.1 M H_2_SO_4_ as a fluorescence standard, from the following equation:

$$\Phi_{F}(x)=\Phi_{F}(s)\frac{F_{x}}{F_{s}}\frac{A_{s}}{A_{x}}$$

where *x* corresponds to the compound under assay and *s* to the quinine standard, *F_x_* and *F_s_* are the integrated areas of the emission spectra, and *A_x_* and *A_s_* are the absorbance values corresponding to the maximum excitation wavelengths. These fluorescence measurements were carried out for diluted solutions showing absorbance values below 0.05 at the excitation wavelengths.

#### 2.4.2. Fluorimetric Characterisation of the Analytical Reaction with ROS

For preliminary experiments, the oxidation reaction was carried out employing solutions of different concentrations of H_2_O_2_ in acetonitrile:water. Appropriate aliquots from these solutions were added to the benzene solution of the sensor (the same solvent employed for the synthetic reaction) with the reaction proceeding in 2–3 min under such conditions.

For the reactions carried out in acetonitrile, stock aqueous solutions of hydrogen peroxide (0.1 M), *tert*-butyl hydroperoxide (0.1 M) and sodium hypochlorite (1.0 × 10^−3^ M) were prepared. The aqueous solutions of hydrogen peroxide and *tert*-butyl hydroperoxide were diluted up to 1.0 × 10^−3^ M in acetonitrile. To explore the analytical reaction in organic media, adequate aliquots (20 μL) of the 1.0 × 10^−3^ M solutions were added to solutions of compounds **A** in acetonitrile so that the final concentration of the sensors was 1.0 × 10^−6^ M, and 1.0 × 10^−5^ M for the oxidants. The water proportion in the working solutions was 0.01:100 v:v water:acetonitrile for oxidants solutions of hydrogen peroxide and *tert*-butyl hydroperoxide and 1:100 v:v water:acetonitrile for sodium hypochlorite. For quantitative assays, serial dilutions of the oxidants were prepared in acetonitrile in the range of 0.0–1.0 × 10^−3^ M. Aliquots of 20 μL of every solution of the oxidants in acetonitrile were added to independent sensor solutions in acetonitrile. The concentration range of oxidants varied from 0 to 7 × 10^−5^ M in the working solution and the final concentration of compounds **A** was 1.0 × 10^−6^ M. Thus, the sensor concentration remained constant, and the oxidant concentration changed in the mentioned range. The proportion of water in the acetonitrile media was not higher than 10% in the case of sodium hypochlorite.

For the reaction time optimization, the experiments were performed at 25 °C in acetonitrile in the fluorimeter quartz cell. The instrument took automatic readings at 1-min intervals over 5 h at fixed wavelengths corresponding to the excitation and emission maxima of the isolated compounds **B**. Subsequently, and in order to reduce concern about possible changes in the properties of the reaction product, the evolution of the fluorescence signal with time was also checked by recording fluorescence emission spectra in 15 min intervals for 5 h.

#### 2.4.3. Determination of Glucose in Aqueous Media Coupled to the Oxidation of Compounds **A**

A glucose stock solution (400 mM in phosphate buffer saline pH 7.4, PBS) was adequately diluted with additional PBS to obtain the calibration and quality control solutions. The working mix consisted of PBS containing glucose oxidase (final concentration into the well 1 U/mL), horseradish peroxidase (final conc. 0.1 U/mL) and A1 (100 μM in DMSO, final DMSO:water ratio 1:10 v:v) or Amplex^®^ Red (Thermo Fisher—Invitrogen, Madrid, Spain) (50 μM in DMSO, final DMSO:water ratio 1:100 v:v). The excitation/emission wavelengths were 360/450 nm in the case of B1 and 571/585 nm for Amplex^®^ Red. A 20 μM H_2_O_2_ solution was used as a positive control, and plain PBS as a blank. A 50 μL aliquot of every calibration or quality control solution, as well as of the positive control and blanks, was placed into a 96-well microplate. A 50 μL aliquot of the working mix were then added to each well. The plates were incubated in the darkness at 37 °C and the fluorescence was measured every 30 min. Slits were set at a 5 nm bandwidth. The recorded emission intensity values were corrected using a reference detector in the excitation beam.

## 3. Results and Discussion

### 3.1. Synthesis

**Scheme 2:**
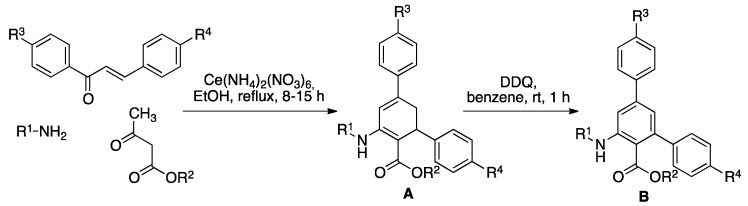
Synthesis of sensors **A** by a three-component reaction from chalcones, primary amines and β-ketoesters and their 2,3-dichloro-5,6-dicyanobenzoquinone (DDQ)-promoted dehydrogenation to furnish compounds **B.**

As summarized in Scheme 2, the preparation of compounds **A** designed as ROS sensors was carried out using the previously reported [38] three-component reaction from chalcones, primary amines and β-ketoesters in refluxing ethanol, in the presence of Cerium(IV) ammonium nitrate (CAN) as a catalyst, with the results shown in Table 1. Due to the need to have the isolated products formed in the analytical reaction in hand for reference purposes, compounds **A** were dehydrogenated in good to excellent yields to the aromatic derivatives **B** by exposure to 2,3-dichloro-5,6-dicyanobenzoquinone (DDQ) at room temperature.

### 3.2. Native Fluorescence Studies

UV-Vis absorption spectra were obtained with the aim of characterizing aromatized compounds **B**, as these data are required for the determination of the fluorescence quantum yields. Compounds **B1**–**B7** showed two absorption maxima in the surroundings of 250 nm and 360 nm, which can be slightly shifted depending on the substituents on the aromatic *m*-terphenyl moiety (R^1^, R^3^ and R^4^). The *N*-aryl derivatives **B8**–**B10** showed more complex spectra, with three peaks at ca. 245, 272 and 364 nm. The spectroscopic absorption parameters of all compounds **B** are summarized in Table S1 (see the Supporting Materials).

**Figure 1:**
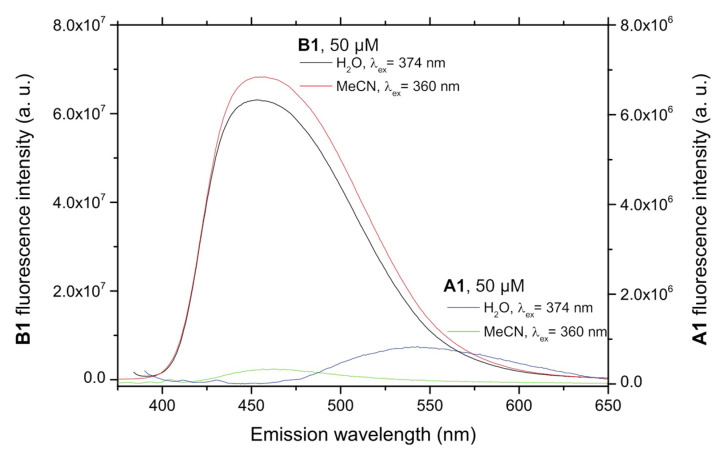
Comparison of the fluorescence spectra of compound **A1** and its dehydrogenation derivative **B1**. Note that the scale corresponding to the non-oxidized probe **A1** is expanded ten-fold so that its emission spectra are visible.

In order to assess whether the desired turn-on fluorescence behavior was possible, the fluorescence of compounds **A** and **B** needed to be studied. Figure 1 shows a comparison of the emission of native **A1** and dehydrogenated **B1** compounds in two solvents of different polarities, showing a remarkably different native fluorescence. The solutions were measured at 5.0 × 10^−5^ M, because no fluorescence was detected for lower concentrations of **A1**, due to its very low native fluorescence.

**Figure 2:**
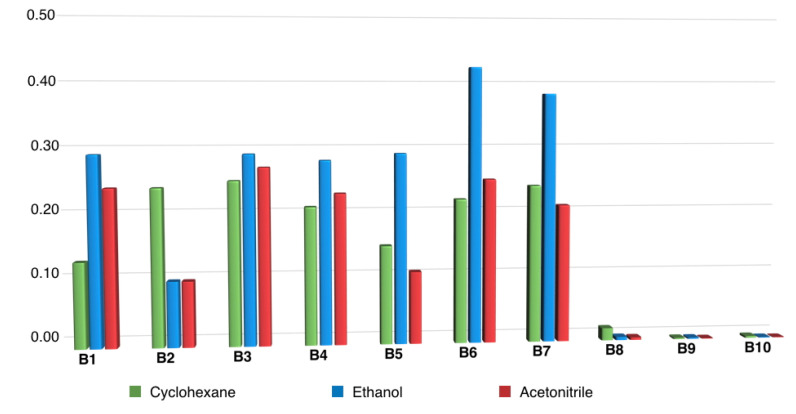
Fluorescence quantum yields of compounds **B**, highlighting the very low native fluorescence of *N-*aryl derivatives **B8**–**B10.**

Generally speaking, that compounds belonging to series **A** were very weakly or not at all fluorescent (fluorescence quantum yields Φ_F_ < 0.001), while significant fluorescence emission was observed in the case of most of their *m*-terphenyl dehydrogenation derivatives (compounds **B**), which was studied in three solvents covering a broad polarity range. Fluorescence emission maxima (Table S2, see Supporting Materials) were located around 450 nm for the *N-*alkyl or *N-*arylalkyl compounds **B1**–**B7**, and it was found that neither the nature of substituents nor solvent polarity had a significant effect on the position of the excitation and fluorescence maxima. Fluorescence quantum yields of compounds **B1**–**B7** were in the 0.10–0.42 range, with some dependence on the chemical structure and the solvent, as shown in Figure 2. Studies performed in aqueous environments (e.g., from a biological sample) will normally involve altering the polarity of the reaction medium by addition of water, so compounds showing large solvent-associated differences in quantum yields cannot be considered ideal probes. In this light, the performance of the *N-*benzyl derivatives **B6** and **B7** might be affected, although it was nevertheless decided to study one of them (**B6**), due to its high fluorescence quantum yields. Among the *N-*alkyl derivatives, **B1**, **B3** and **B4** offered a good compromise between high fluorescence yield and insensitivity to solvent polarity, especially for the water-miscible solvents in which the analytical reaction is more likely to be developed, with **B1** being finally chosen due to its better solubility.

On the other hand, a remarkable effect of *N-*aryl substituents (compounds **B8**–**B10**) on the fluorescence properties was noticed. In these cases, the fluorescence emission maximum is red-shifted in comparison to compounds **B1**–**B7**, and the fluorescence emission is almost totally quenched, as shown by the negligible fluorescence quantum yields. These observations show that the analytical oxidation reaction is not the only factor to be considered, and that photophysical issues associated with the reaction products can also play an important role. 

**Figure 3:**
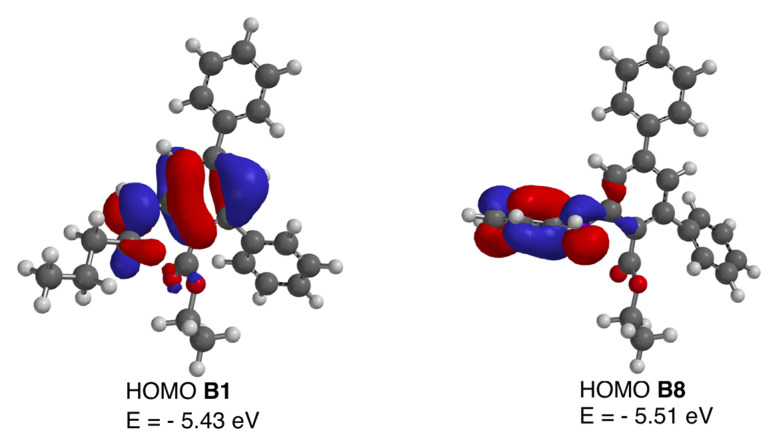
HOMO molecular orbitals of compounds **B1** and **B8**.

In order to explain the abrupt difference between the *N-*alkyl or *N-*arylalkyl derivatives **B1**–**B7** and their *N-*aryl counterparts **B8**–**B10**, DFT calculations were undertaken on **B1** and **B8** as representative examples. The geometries of both classes of compounds at the anthranilate moiety are similar, and, thus, the calculated N-C2-C1-C(O) dihedral angle is 4.79° for **B1** and 3.51° for **B8**. The lack of fluorescence of compounds **B8**–**B10** can be explained by an intramolecular electron transfer (ICT) process, where the lone electron pair on the amine nitrogen is delocalized through the phenyl substituent, which acts as an acceptor and hampers electron donation to the terphenyl system. However, in **B1**, the amino group acts as an electron donor towards the terphenyl system, whereas in the case of **B8** it donates electron density to the *N-*aryl substituent. This effect can be verified by comparing the HOMO orbitals of compounds **B1** and **B8**, which are shown in Figure 3. This conjugation is geometrically feasible, since the C2′-C1′-N-C2 dihedral angle in **B8** was found to be 6.22°. 

### 3.3. Dihydro-m-Terphenyls A for ROS Fluorimetric Determination

**Figure 4:**
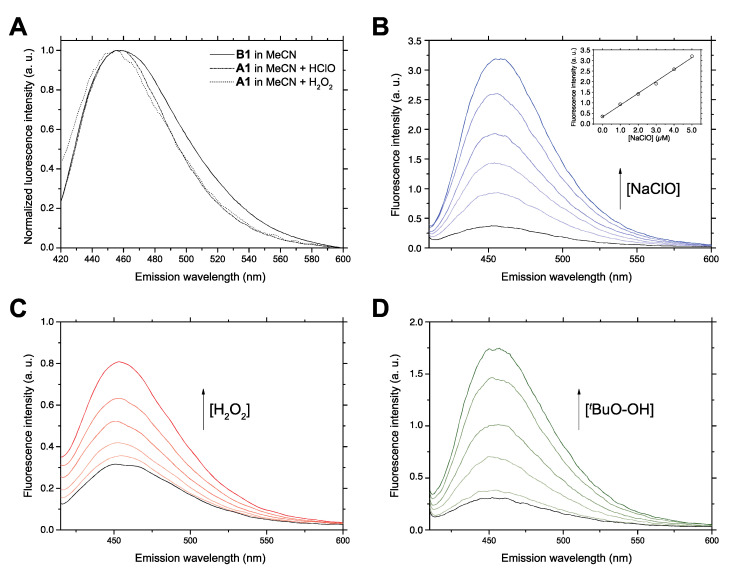
(**A**) Overlaid fluorescence emission spectra (λ_ex_ 360 nm) of synthetic compound **B1** and those obtained by oxidation of compound **A1** under analytical conditions, i.e., from a reaction medium containing **A1** and H_2_O_2_ or NaClO in acetonitrile-water (the signals were normalized in order to allow better comparison of the spectral shapes and position of the maxima). (**B**) Fluorescence emission spectra (λ_ex_ 364 nm) of the reaction product of compound **A1** with increasing amounts of sodium hypochlorite. Inset: calibration curve corresponding to fluorimetric titration at fixed excitation and emission wavelengths. (**C**,**D**) The same experiments performed with hydrogen peroxide and *tert*-butyl hydroperoxide, respectively. In panels B–D, the trace in black corresponds to the absence of oxidant (c = 0) and the ones corresponding to increasing amounts of antioxidant (c = 1.0 × 10^−6^ M, 2.0 × 10^−6^ M, 3.0 × 10^−6^ M, 4.0 × 10^−6^ M and 5.0 × 10^−6^ M) are now shown in varying shades of color. In panels **B**-**D**, the values of fluorescence intensity in arbitray units (a.u.) should be multiplied by 10^5^.

Considering the fluorescence quantum yields found for the dehydrogenated derivatives **B** compounds **A1** and **A6** were chosen as representative *N-*alkyl and *N-*arylalkyl chemodosimeters for optimization of the analytical reaction conditions. As a proof-of-concept experiment, Figure 4A shows the evolution of its non-fluorescent precursor **A1** in acetonitrile in the presence of H_2_O_2_ and HClO. Fluorescence emission was developed after a few minutes and the obtained emission spectra showed an excellent agreement with the fluorescence emission spectra of the isolated aromatic compound **B1** in acetonitrile (Figure 4A). This preliminary experiment showed that the fluorescence developed by compounds **A** in the presence of ROS species is due to the corresponding compound **B**, and can be exploited for the fluorometric detection of ROS. While water proved an unsuitable reaction medium because of the very low aqueous solubility of compounds **A**, both acetonitrile and ethanol could be employed for the analytical process, and the reaction media could contain up to 10% water without any significant loss in response, making the reaction compatible with the addition of biological samples. The best results in terms of reaction rate were obtained in the polar, non-protic solvent acetonitrile, which was employed for all subsequent work using hydrogen peroxide, *tert*-butyl hydroperoxide and sodium hypochlorite as analytes. 

In the next step, the linear relationship between fluorescence signal and ROS concentration and the influence of reaction time on the analytical response were studied. In order to facilitate the reproducible and accurate quantitative assay of ROS using the compounds described here, the kinetics of the reactions starting from **A1** were monitored in order to determine an optimal assay time, and also included **A6** in the study. For analytical purposes, 30 min was established as a suitable reaction time for the analytical ROS assays. Figure 4B shows the successive fluorescence emission spectra of an acetonitrile solution containing compound **A1**, taken 30 min after the addition of increasing amounts of sodium hypochlorite. The linear regression analysis of the calibration curve obtained by plotting fluorescence signal against NaClO concentration (shown inset) showed a good linear response (*R^2^* = 0.999). Similar results were obtained for hydrogen peroxide (Figure 4C) and *tert*-butyl hydroperoxide (Figure 4D). Compound **A6** was also assayed with the same oxidants as shown in the Supporting Materials (Figures S1–S3). In all cases, the linearity of the response was good (*R^2^* > 0.95), although **A6** gave poorer analytical figures (see below). Due to the very low quantum yield obtained for the isolated compound **B8**, the fluorescent response observed for **A8** in the presence of sodium hypochlorite was negligible, even when the measurements were extended to 21 h (Figure S4).

With the aim of studying the potential analytical application of **A1** and **A6** as chemodosimeters in organic media, analytical figures of merit, i.e., the limit of detection (LOD), limit of quantitation (LOQ), and precision were determined in acetonitrile for a reaction time of 30 min. To obtain the calibration curves and the regression parameters, five replicates of every titration were carried out and the fluorescence intensities at the excitation and emission maxima were measured and processed. LOD and LOQ were determined based on the calibration curve [39], and they were calculated as the concentration interpolated from the signal value corresponding to the ordinate in the origin of the calibration curve plus three times its standard deviation (LOD), or plus ten times its standard deviation (LOQ). Satisfactory results were obtained, including linear range from 0–50 μM of oxidants (*R^2^* of 0.99), with a single exception (**A6** with hypochlorite) where the linear range was 0–70 μM, LOD of 1 μM and precision lower than 5%, evaluated as relative standard deviation (%RSD) at two levels of concentrations (Table 2; Table 3). The overall analytical properties of **A1** were better than those of **A6**, in terms of the evolution of fluorescence response with time and stability of the response at the selected reaction time. This difference was not wholly unexpected, taking into consideration the previously discussed deleterious influence of abrupt solvent polarity changes in its fluorescence quantum yield. Furthermore, LOQ values obtained for **A1** (ca. 1 μM) are better than those obtained for **A6** (ca. 10 μM), as shown in Table 2

**Figure 5:**
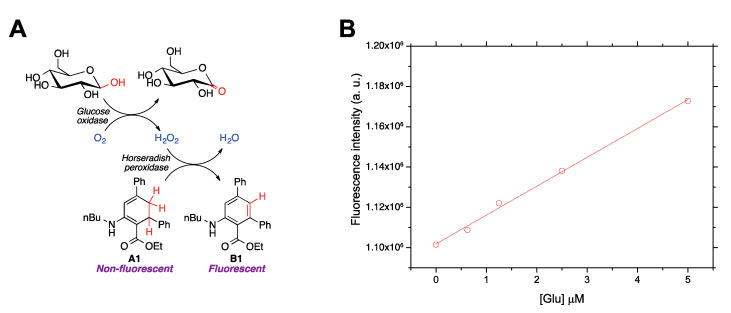
(**A**) Enzymatic reaction cascade coupling glucose oxidation to fluorescence detection of H_2_O_2_ by using compound **A1** as a chemodosimeter. (**B**) Calibration curves showing the dependence of the developed fluorescence, due to the formation of **B1**, on glucose concentration.

Finally, to demonstrate the ability of compound **A1** to detect ROS in aqueous environments and in a biological context, this molecule was employed to quantify glucose by enzymatically coupling glucose oxidation to H_2_O_2_ production (Figure 5a). Amplex^®^ Red, a well-established ROS sensor [40,41], was employed as a reference. ROS quantitation in aqueous media was carried out in a miniaturized format using a 96-well plate. This device made it possible to analyze simultaneously a large number of samples in a short time and using a reaction volume as low as 100 μL. **A1** was incubated with the glucose samples at 37 °C up to 120 min in presence of glucose oxidase and horseradish peroxidase in aqueous buffered media. Dimethyl sulfoxide (DMSO) was added in order to ensure the full dissolution of **A1** while maintaining enzymatic activity. Several DMSO-water ratios were assayed (5%, 7%, and 10%), and the best analytical figures of merit were obtained for the latter case (Figure 5b). The fluorescent emission was measured at the excitation and emission maxima corresponding to **B1**, providing a satisfactory fluorescence response vs. H_2_O_2_ (i.e., glucose) concentration. Good analytical features were obtained, which were similar to those of Amplex^®^ Red, but measurements with **A1** provided a lower relative error (Table 4). These data further demonstrate the usefulness of **A1** as a fluorescence chemodosimeter for H_2_O_2_, and therefore also for biologically interesting molecules producing H_2_O_2_ in aqueous environments.

## 4. Conclusions

In conclusion, dihydro-*m*-terphenyls bearing an anthranilate moiety in their central ring are easily prepared from simple open-chain starting materials via a three-component reaction catalyzed by CAN. These compounds behave as structurally novel fluorescent chemodosimeters that yield highly fluorescent dehydrogenation derivatives in the presence of biologically relevant oxidants, such as hydrogen peroxide, sodium hypochlorite, and a model hydroperoxide in an experimentally convenient, fast reaction not requiring enzymatic catalysis. The fluorescence signal is quickly developed in the presence of trace amounts of the probe and the analytes in acetonitrile media at room temperature with good analytical figures. The suitability of each particular dihydroterphenyl derivative as a ROS chemodosimeter depends not only on its successful aromatization in the presence of ROS, but also on photophysical phenomena associated to its structure, with *N-*alkyl derivatives exhibiting the best behavior, since they were not subject to intramolecular electron transfer (ICT) phenomena that detracted from their fluorescence.

The compounds presented here act as turn-on sensors, a highly desirable property for a fluorescent sensor, because it avoids the practical problems associated with the measurement of an optical signal against a bright background. The analytical reaction using **A1** as fluorescence sensor, coupled to enzymatic catalysis has been applied in microscale assay using a 96-well microplate device for the quantitation of glucose mediated by H_2_O_2_ production, showing analytical performance comparable to that of a commercial kit. One additional advantage of the compounds proposed here is the presence of amino and ester functional groups, which will allow their future functionalization to improve their physico-chemical properties, while maintaining fluorescence emission. Thus, hydrophilic esters, e.g., polyethyleneglycol derivatives, which can avoid the use of DMSO in the reaction media, should be readily available via transesterification or hydrolysis followed by esterification. Similarly, conjugation to biocompatible polymers for targeting purposes or as a first step towards building sensor devices should also be feasible.

## Figures and Tables

**Table 1:** Structures and yields of compounds **A** and **B** ^a^.

Compound	R^1^	R^2^	R^3^	R^4^	A, %	B, %
**1**	(CH_2_)_3_CH_3_	CH_2_CH_3_	H	H	88	93
**2**	(CH_2_)_3_CH_3_	CH_2_CH_3_	Cl	H	70	92
**3**	(CH_2_)_3_CH_3_	CH_2_CH_3_	H	CH_3_	72	91
**4**	(CH_2_)_3_CH_3_	CH_2_CH_3_	H	OCH_3_	70	89
**5**	(CH_2_)_3_CH_3_	C(CH_3_)_3_	H	H	70	88
**6**	CH_2_Ph	CH_2_CH_3_	H	H	78	78
**7**	CH_2_Ph	C(CH_3_)_3_	H	H	80	78
**8**	Ph	CH_2_CH_3_	H	H	73	94
**9**	4-MeC_6_H_4_	CH_2_CH_3_	H	H	72	92
**10**	4-MeOC_6_H_4_	CH_2_CH_3_	H	H	76	91

^a^ Compounds **1**, **6**, **7** and **8** were known in the literature [38].

**Table 2:** Analytical sensitivity of the analytical reaction studied in acetonitrile.

Sensor	Analyte	Linear range	R^2^	LOD	LOQ
**A1**	H_2_O_2_	0–5.0 × 10^−5^ M	0.9993	1.08 × 10^−6^ M	3.59 × 10^−6^ M
	NaClO	0–5.0 × 10^−5^ M	0.9954	2.75 × 10^−6^ M	9.15 × 10^−6^ M
	*^t^*BuO-OH	0–5.0 × 10^−5^ M	0.9908	3.91 × 10^−6^ M	1.30 × 10^−5^ M
**A6**	H_2_O_2_	0–5.0 × 10^−5^ M	0.9909	3.88 × 10^−6^ M	1.29 × 10^−5^ M
	NaClO	0–7.0 × 10^−5^ M	0.9645	9.07 × 10^−6^ M	2.48 × 10^−5^ M
	*^t^*BuO-OH	0–5.0 × 10^−5^ M	0.9719	6.98 × 10^−6^ M	2.33 × 10^−5^ M

**Table 3:** Precision evaluated as relative standard deviation (%RSD) of the analytical reaction determined at two concentrations levels, in acetonitrile.

Sensor	Analyte	Concentration	% RSD (*n* = 5) ^a^
**A1**	H_2_O_2_	1.0 × 10^−5^ M	1.3
	H_2_O_2_	3.0 × 10^−5^ M	2.4
	*^t^*BuO-OH	1.0 × 10^−5^ M	1.6
	*^t^*BuO-OH	3.0 × 10^−5^ M	4.6
	ClO^-^	1.0 × 10^−5^ M	2.6
	ClO^-^	3.0 × 10^−5^ M	2.1
**A6**	H_2_O_2_	1.0 × 10^−5^ M	3.6
	H_2_O_2_	3.0 × 10^−5^ M	4.6
	*^t^*BuO-OH	1.0 × 10^−5^ M	6.9
	*^t^*BuO-OH	3.0 × 10^−5^ M	5.6
	ClO^-^	1.0 × 10^−5^ M	11.0
	ClO^-^	3.0 × 10^−5^ M	7.0

^a^ RSD: Relative standard deviation for five replicates.

**Table 4:** Analytical features of chemodosimeter **A1** applied to glucose quantitation in aqueous media.

Linear Range	*R^2^*	% RSD (*n* = 6) ^a^		[Glucose] Found with A1	[Glucose] Found with Amplex^®^ Red ^b^
		1.25 μM	10 μM	5.0 μM	5.0 μM
0–5 μM	0.9970	6.56	2.35	4.96 μM (−0.8%)	4.86 μM (−2.8%)

^a^ RSD: Relative standard deviation at two levels of concentrations for 6 replicates. ^b^ For Amplex^®^ Red, the percentage of DMSO in the reaction media was 1%. Experimental concentrations of glucose were determined at a theoretical concentration of 5.0 μM. The relative error for each probe is given in parentheses.

## Supplementary Materials

The following are available online at https://www.mdpi.com/2076-3921/9/7/605/s1, Copies of NMR spectra of all compounds, Figure S1: Fluorescence emission spectra (λ_ex_ 360 nm) of the product obtained from the reaction of **A6** with increasing amounts of sodium hypochlorite, Figure S2: Fluorescence emission spectra (λ_ex_ 360 nm) of the product obtained from the reaction of **A6** with increasing amounts of hydrogen peroxide, Figure S3: Fluorescence emission spectra (λ_ex_ 360 nm) of the product obtained from the reaction of **A6** with increasing amounts of *tert*-butyl hydroperoxide; Figure S4: Fluorescence emission spectra (λ_ex_ 361 nm) of the product obtained from the reaction of compound **A8** with the maximum amount of sodium hypochlorite employed for all other cases, after increasing the reaction time up to 21 h.
