# Supplementary material for: Antioxidants as Molecular Probes: Structurally Novel Dihydro-m-Terphenyls as Turn-On Fluorescence Chemodosimeters for Biologically Relevant Oxidants
Source: Antioxidants (Basel). 2020 Jul 10;9(7):605. doi: 10.3390/antiox9070605 (PMC7402136; doi:10.3390/antiox9070605)
Supplement: Supplementary file 1 [file antioxidants-09-00605-s001.pdf]

Supplementary materials

Antioxidants as molecular probes: Structurally novel dihydro-*m*-terphenyls as turn-on fluorescence chemodosimeters for biologically relevant oxidants

Víctor González-Ruiz, [1,†] Jegathalaprathaban Rajesh, [1,‡] Ana I. Olives, [1] Damiano Rocchi, [2] Jorge Gómez-Carpintero, [2] Juan F. González, [2] Vellaisamy Sridharan, [3] M. Antonia Martín [1,*] and J. Carlos Menéndez [2,*]

[1] Unidad de Química Analítica, Departamento de Química en Ciencias Farmacéuticas. Facultad de Farmacia, Universidad Complutense, 28040 Madrid, Spain; aiolives@ucm.es, mantonia@ucm.es

[2] Unidad de Química Orgánica y Farmacéutica, Departamento de Química en Ciencias Farmacéuticas. Facultad de Farmacia, Universidad Complutense, 28040 Madrid, Spain; jgomez21892@gmail.com; rocchid83@gmail.com; jfgonzalez@ucm.es, josecm@ucm.es

[3] Department of Chemistry and Chemical Sciences, Central University of Jammu. Rahya-Suchani (Bagla), District-Samba, Jammu-181143, J&K, India; vesridharan@gmail.com.

[‡] Present address: Department of Chemistry, Sethu Institute of Technology. Pullor, Kariapatti - 626 115 Virudhunagar (D.T), Tamil Nadu, India; mkuraji@gmail.com.

[†] Present address: Analytical Sciences, School of Pharmaceutical Sciences and Institute of Pharmaceutical Sciences of Western Switzerland, University of Geneva, 1 rue Michel-Servet, 1205 Geneva, Switzerland; victor.gonzalez@unige.ch. ORCID: 0000-0001-7204-2363.

[*] Correspondence: mantonia@ucm.es (MAM) and josecm@ucm.es (JCM).

1. Copies of NMR spectra

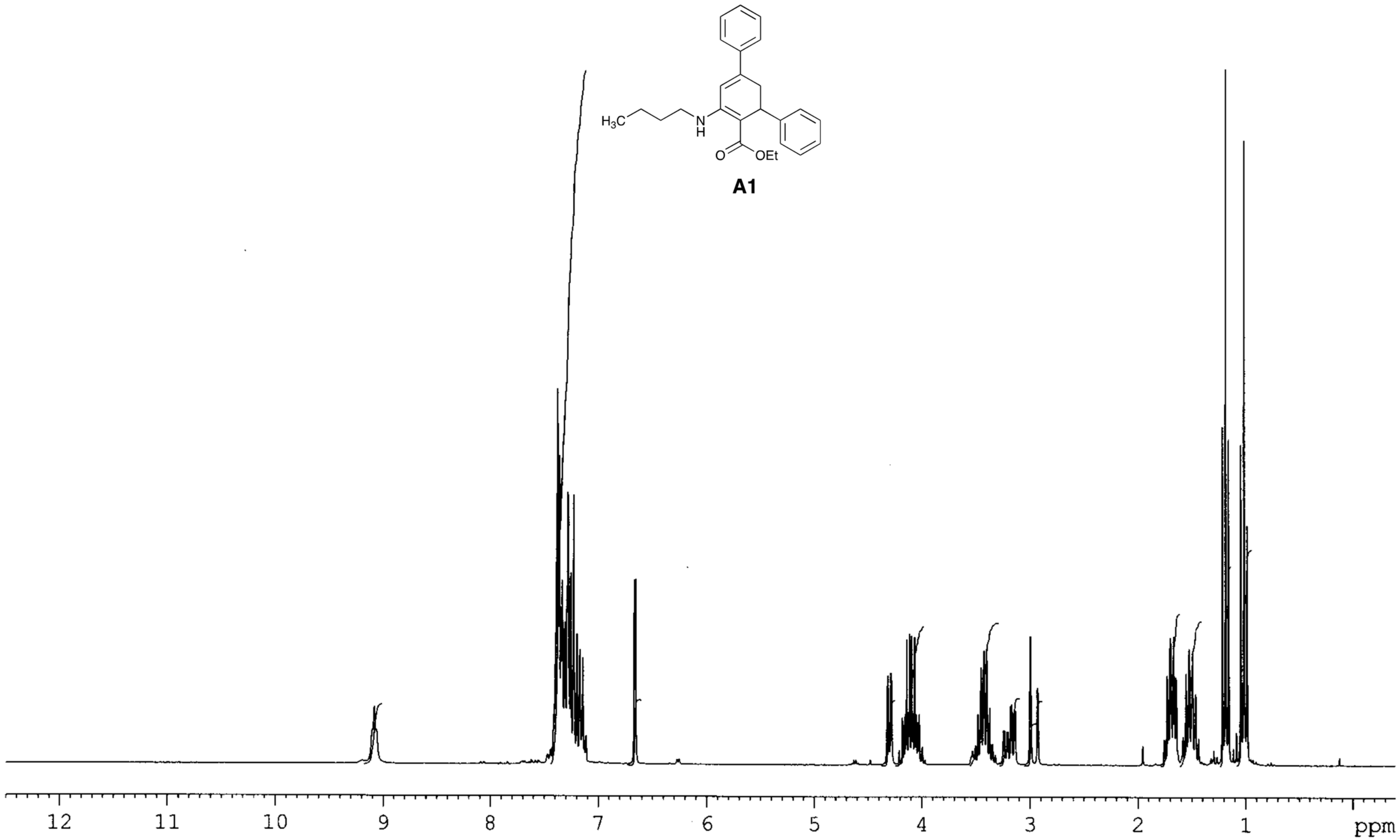

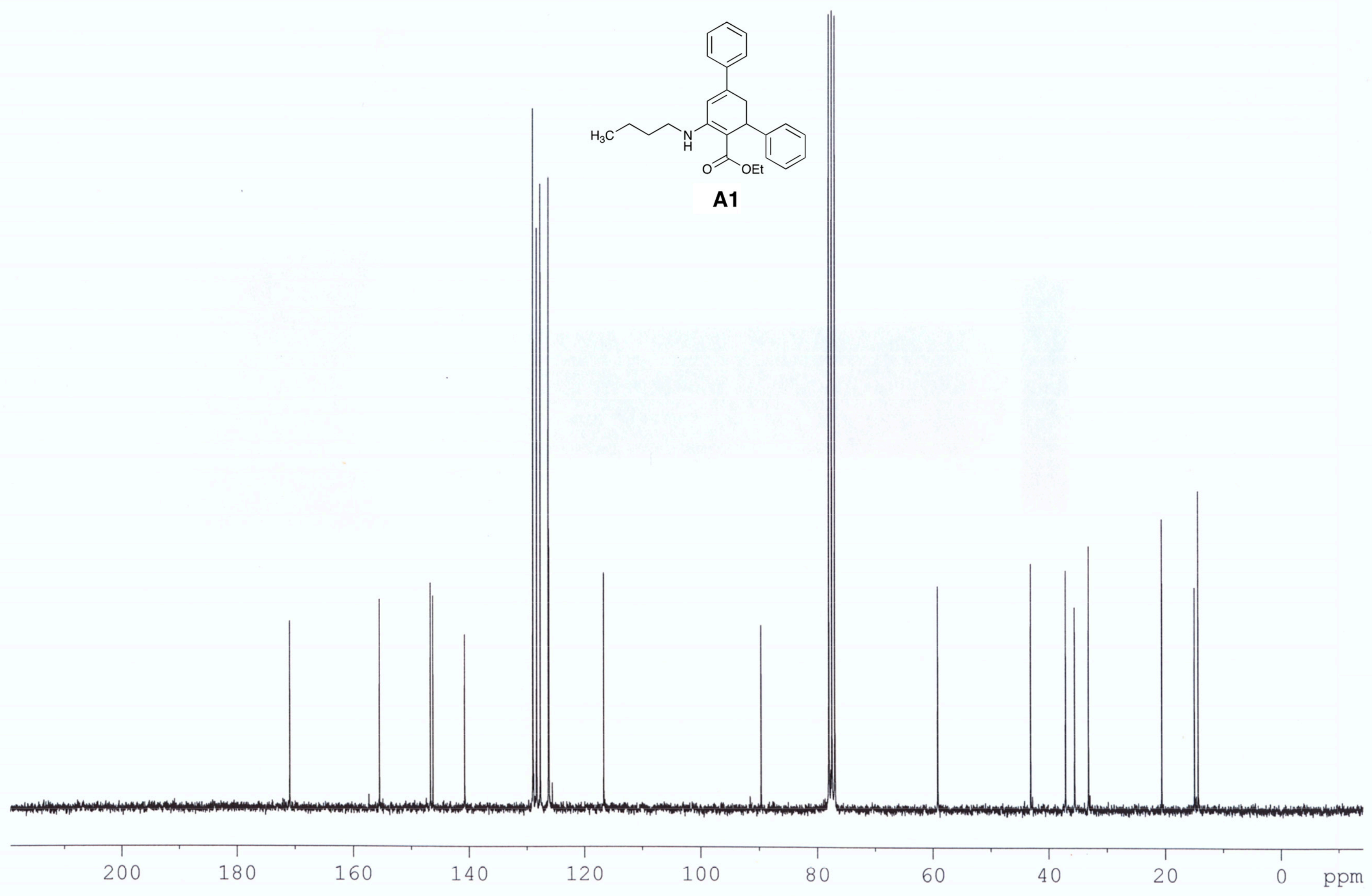
H3C
N
H
O
OEt
A1
200
180
160
140
120
100
80
60
40
20
0
ppm

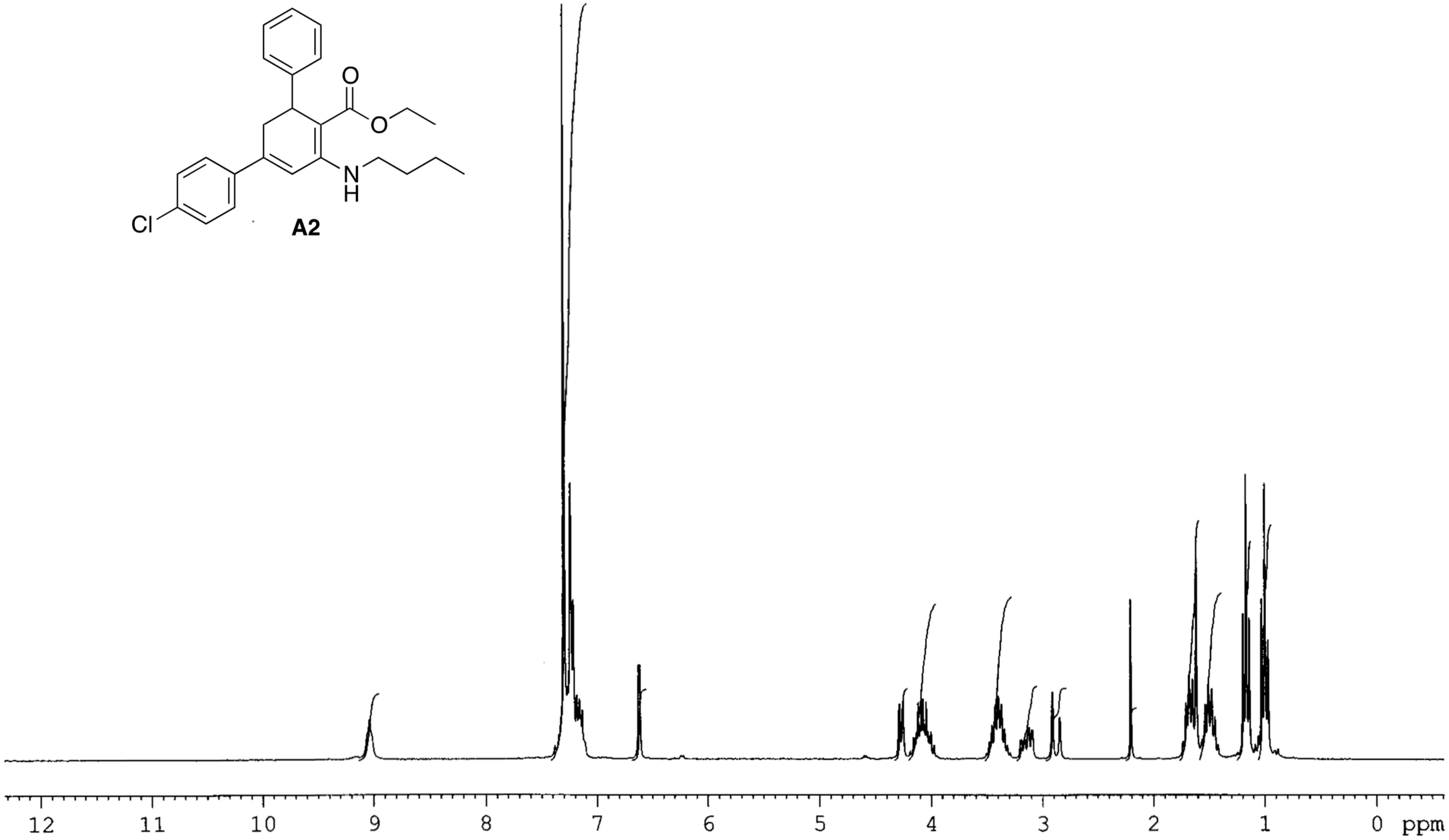

A2

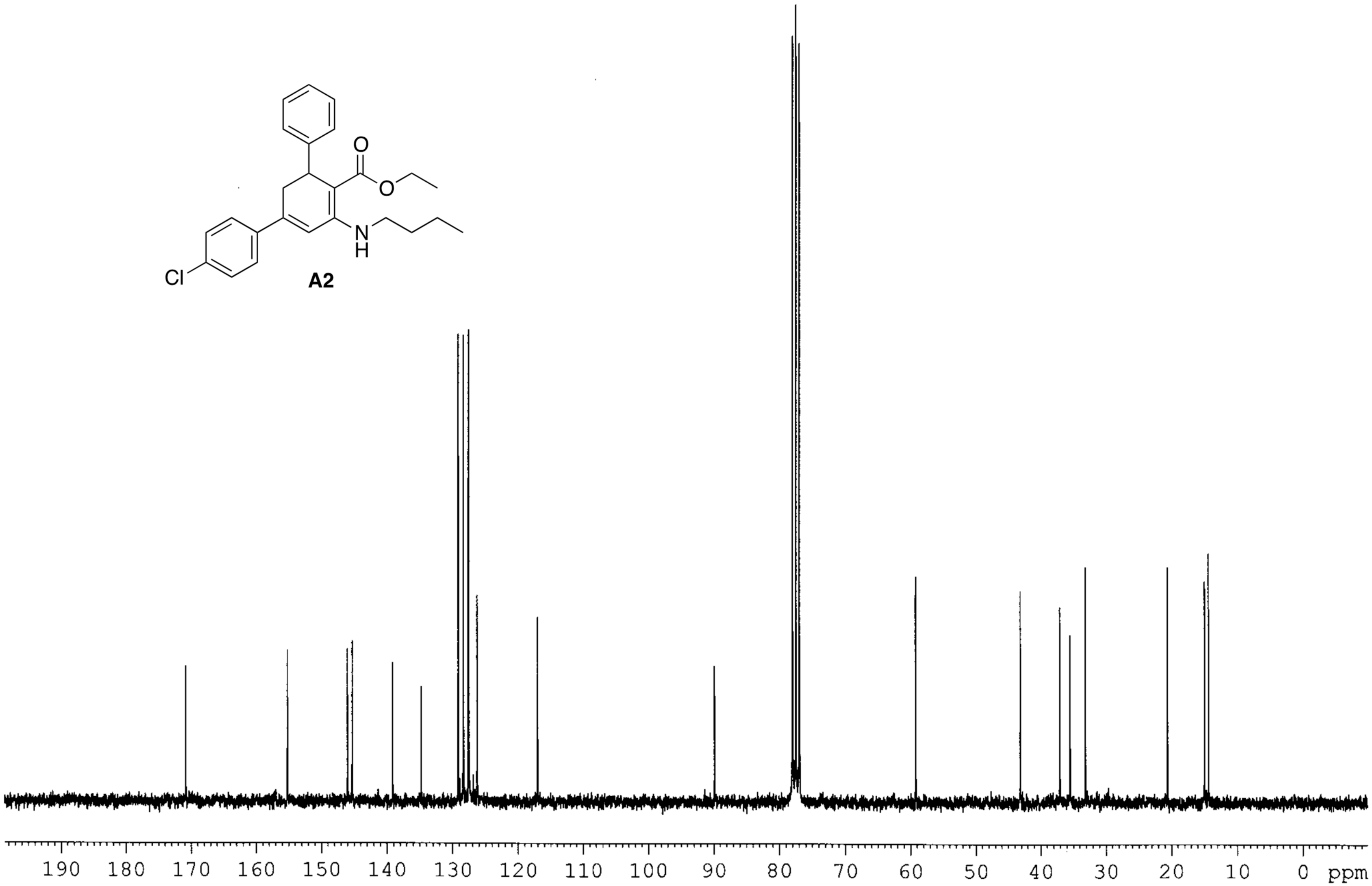
O
O
N
H
Cl
A2
190 180 170 160 150 140 130 120 110 100 90 80 70 60 50 40 30 20 10 0 ppm

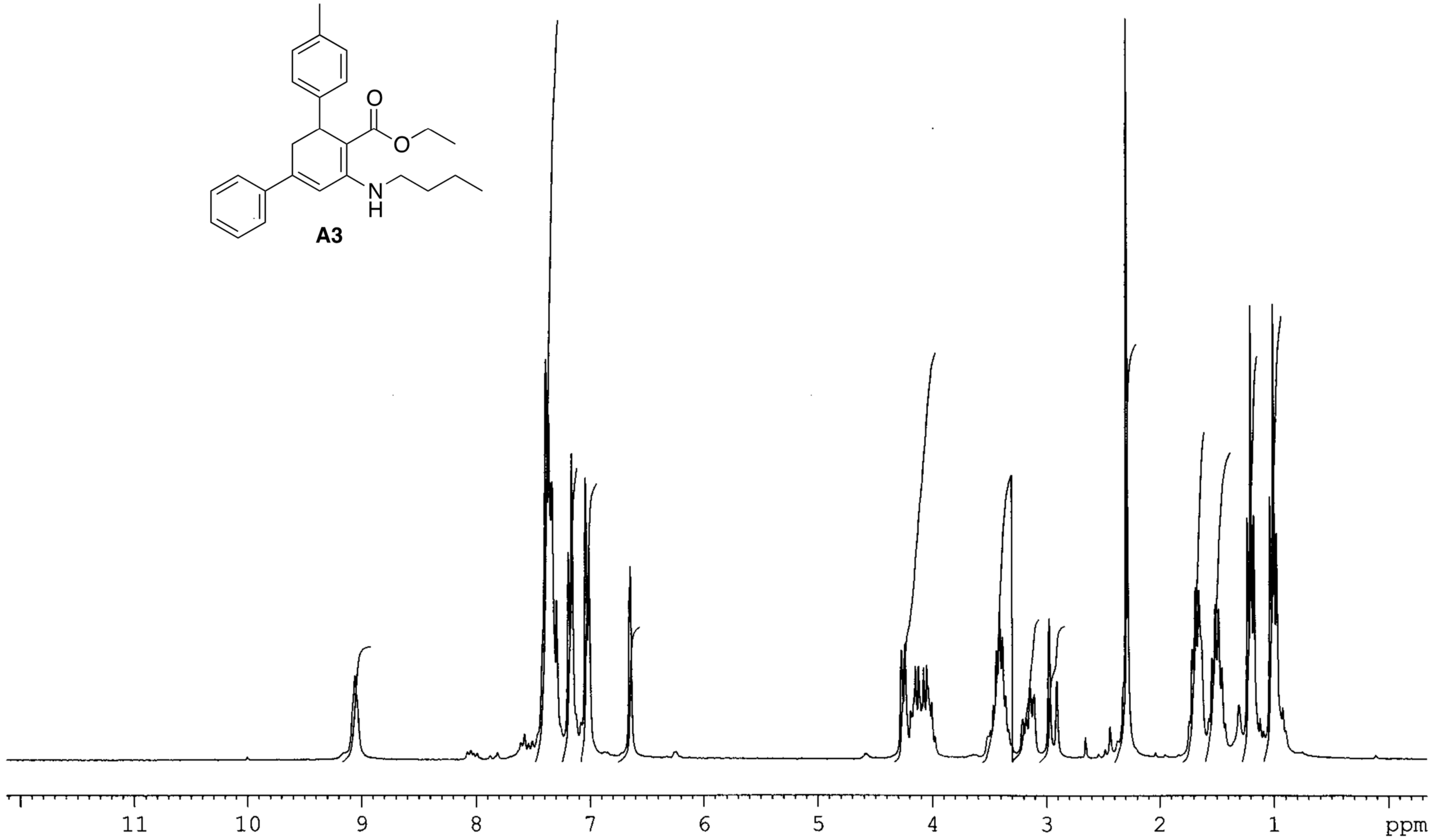
A3
11
10
9
8
7
6
5
4
3
2
1
ppm

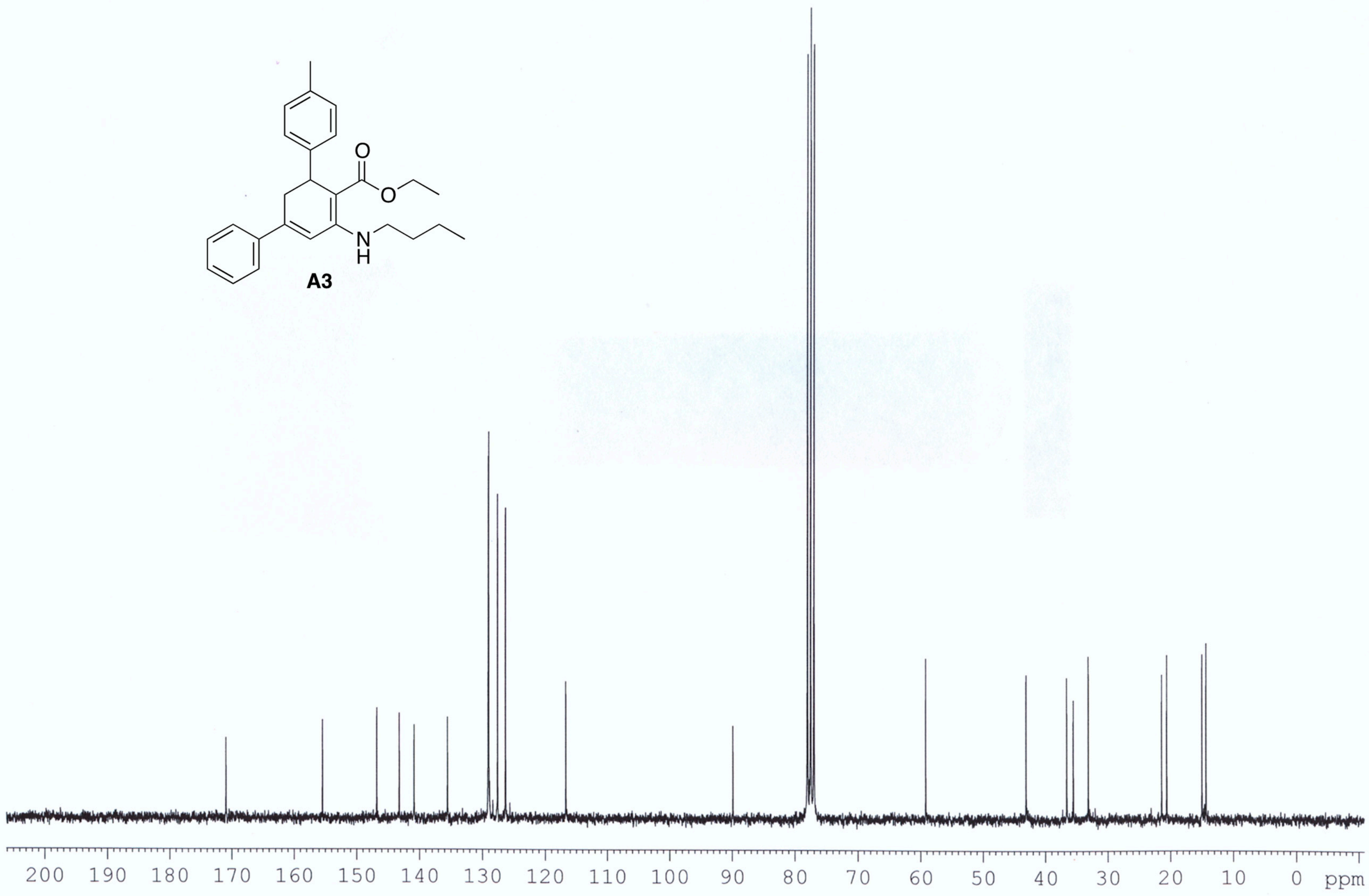
O
O
N
H
A3
200 190 180 170 160 150 140 130 120 110 100 90 80 70 60 50 40 30 20 10 0 ppm

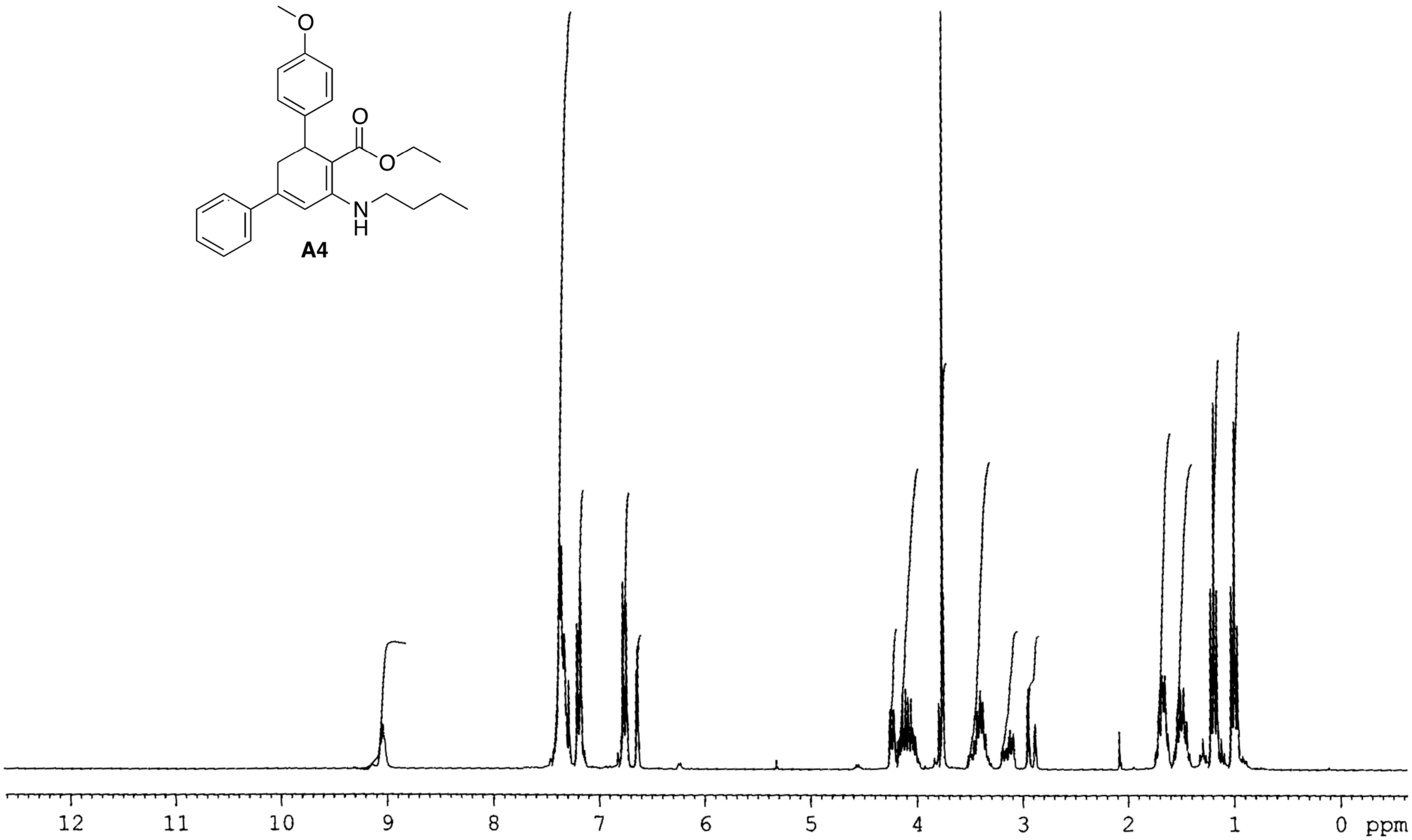

A4

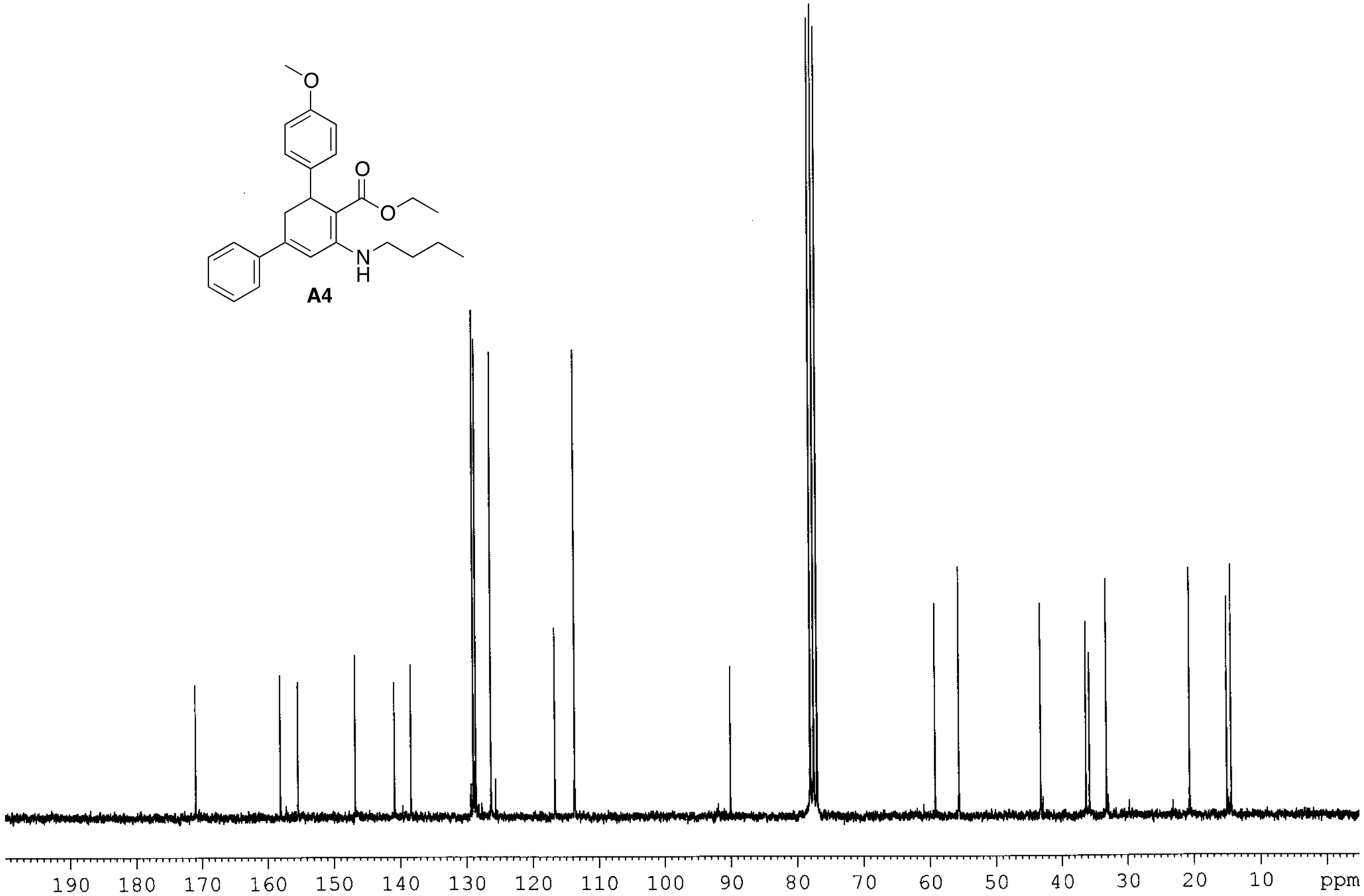
A4
190 180 170 160 150 140 130 120 110 100 90 80 70 60 50 40 30 20 10 ppm

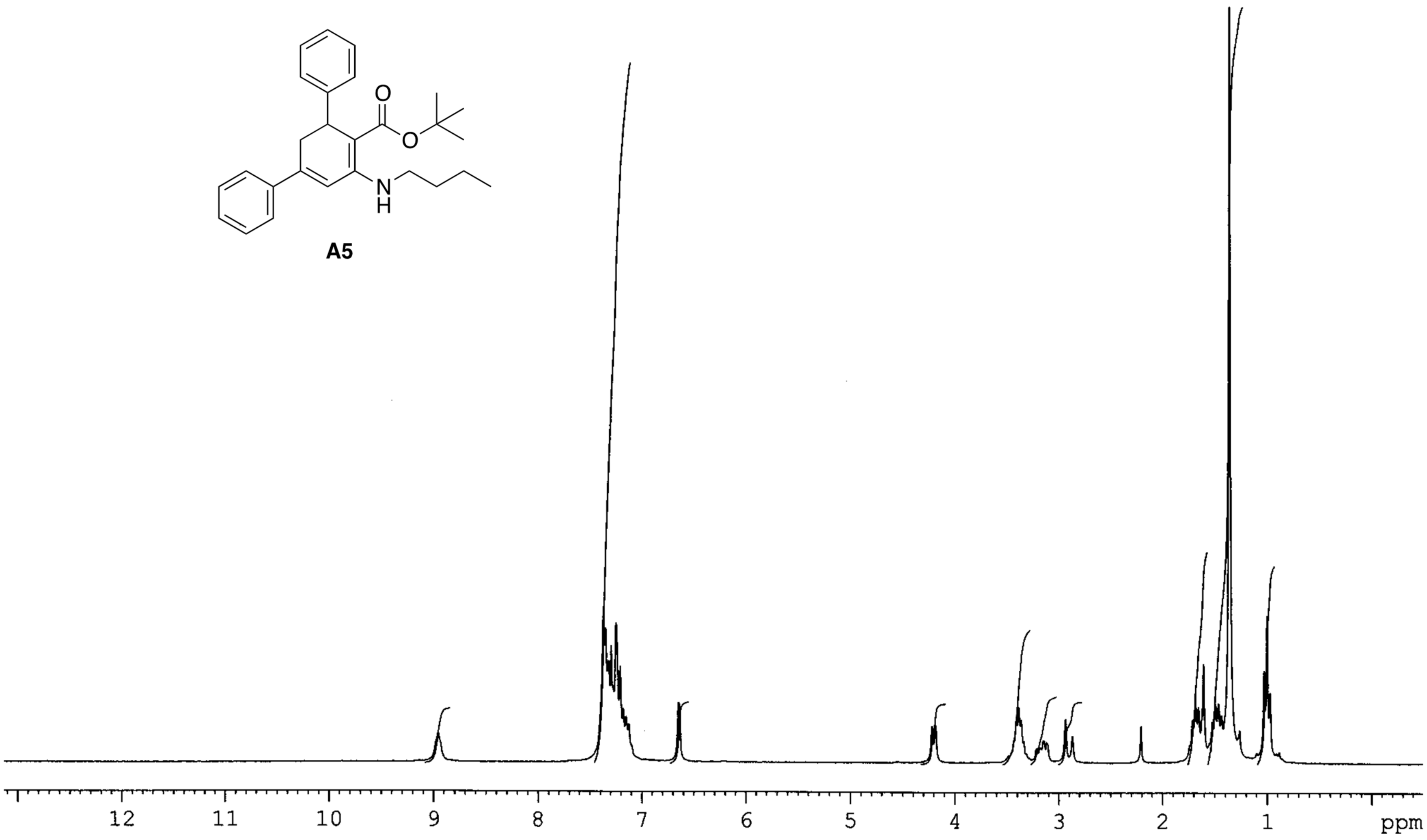
A5
12
11
10
9
8
7
6
5
4
3
2
1
ppm

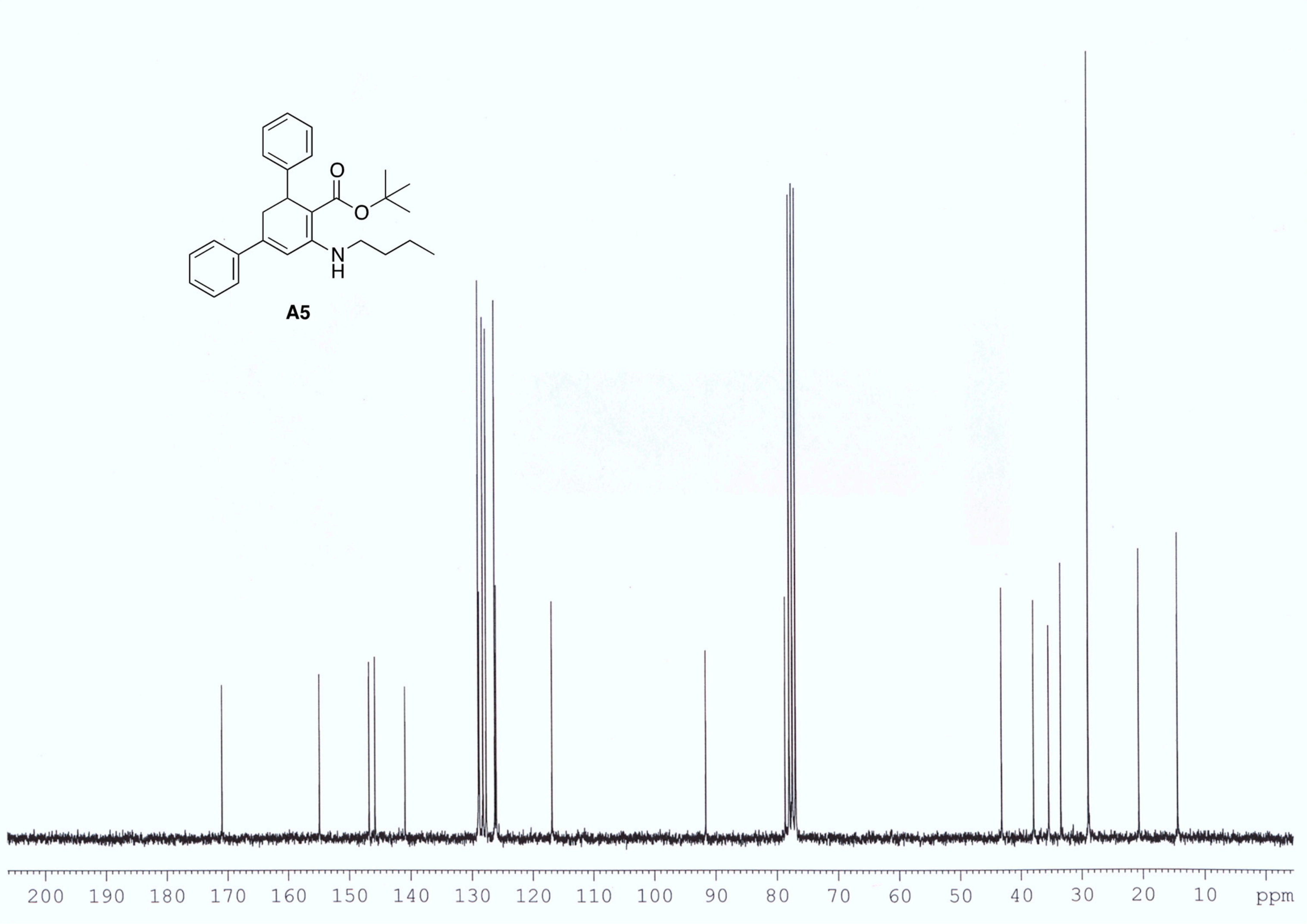

A5
200 190 180 170 160 150 140 130 120 110 100 90 80 70 60 50 40 30 20 10 ppm

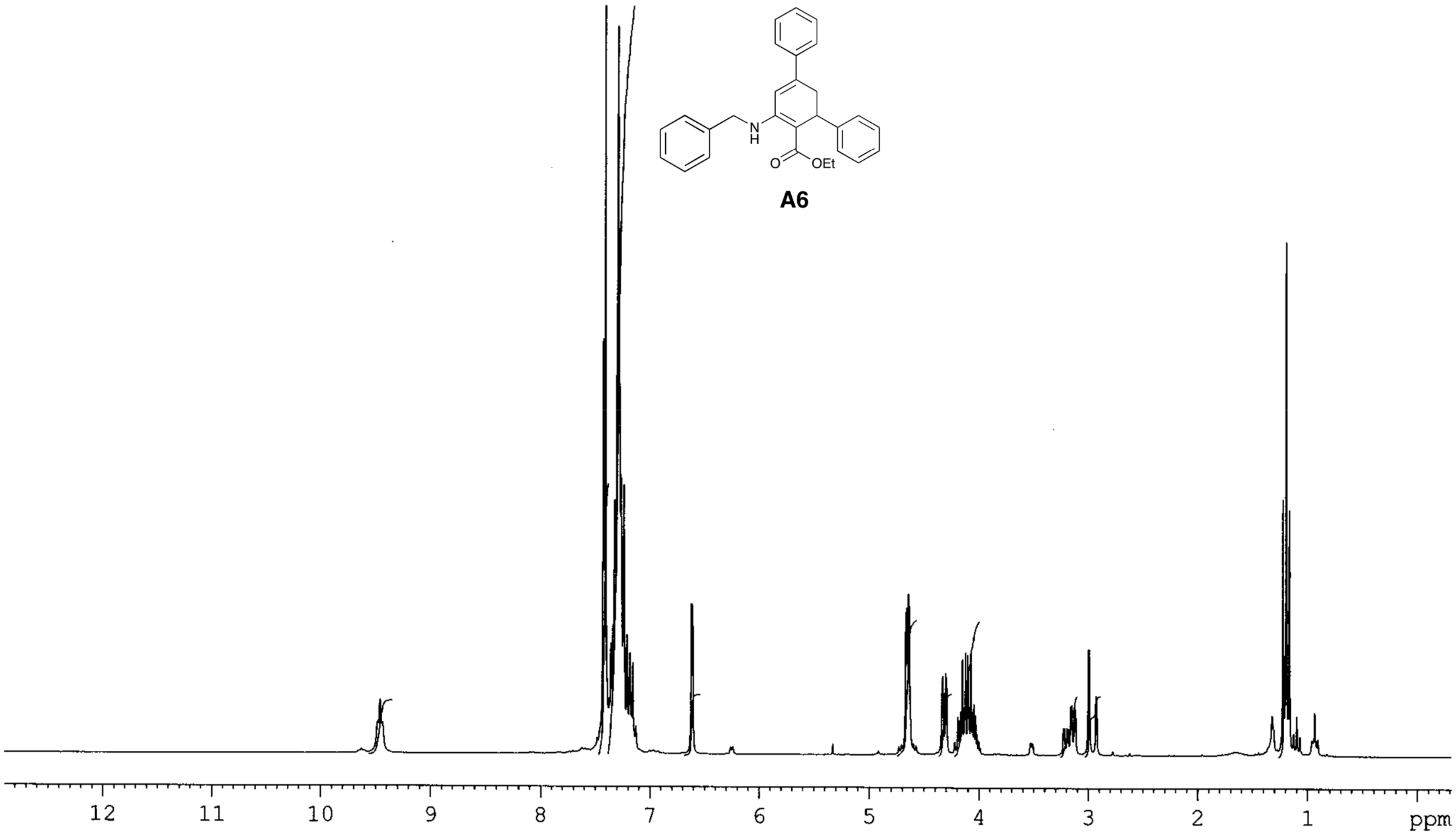

N
H
O
OEt
A6
12
11
10
9
8
7
6
5
4
3
2
1
ppm

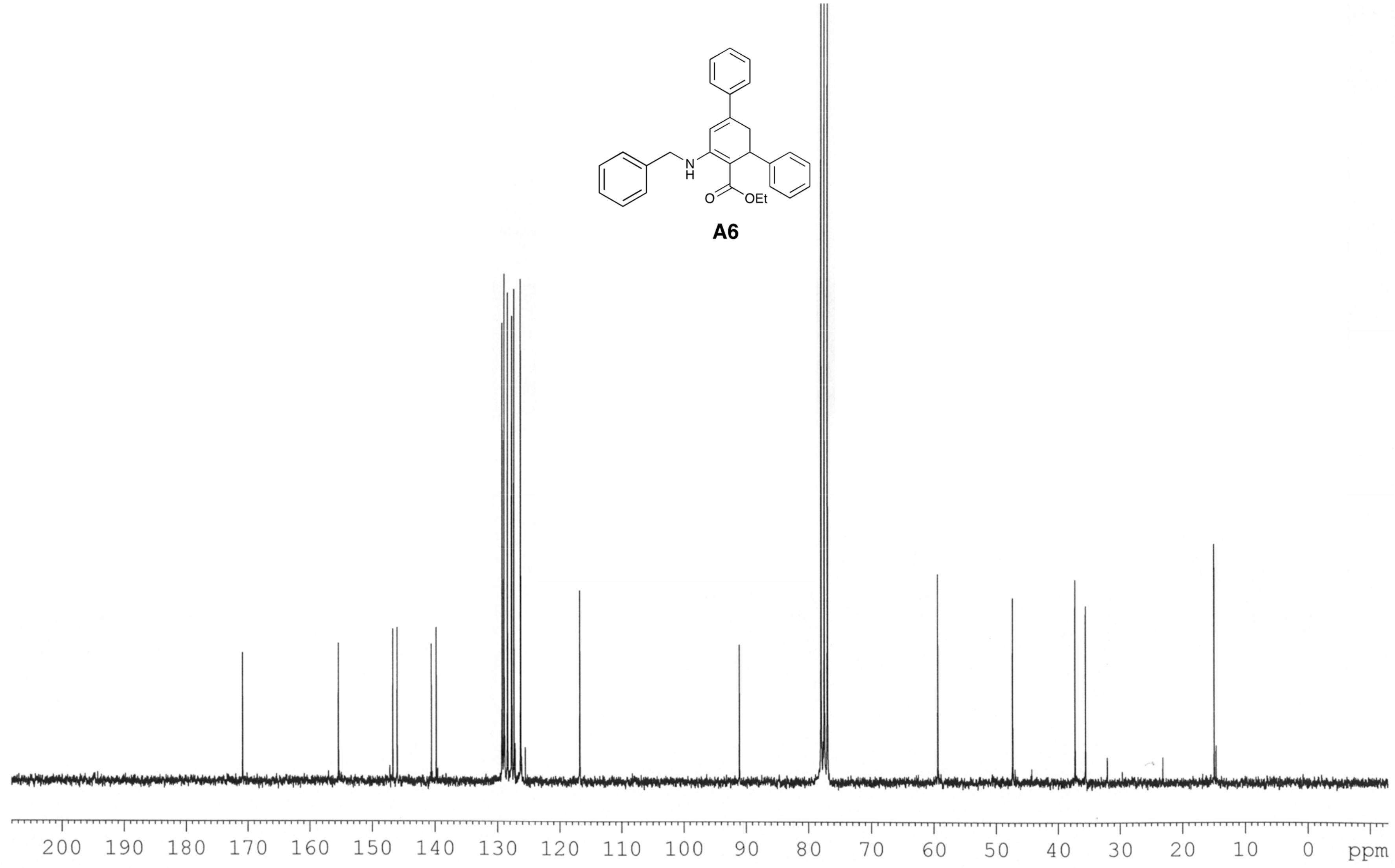
N
H
O
OEt
A6
200 190 180 170 160 150 140 130 120 110 100 90 80 70 60 50 40 30 20 10 0 ppm

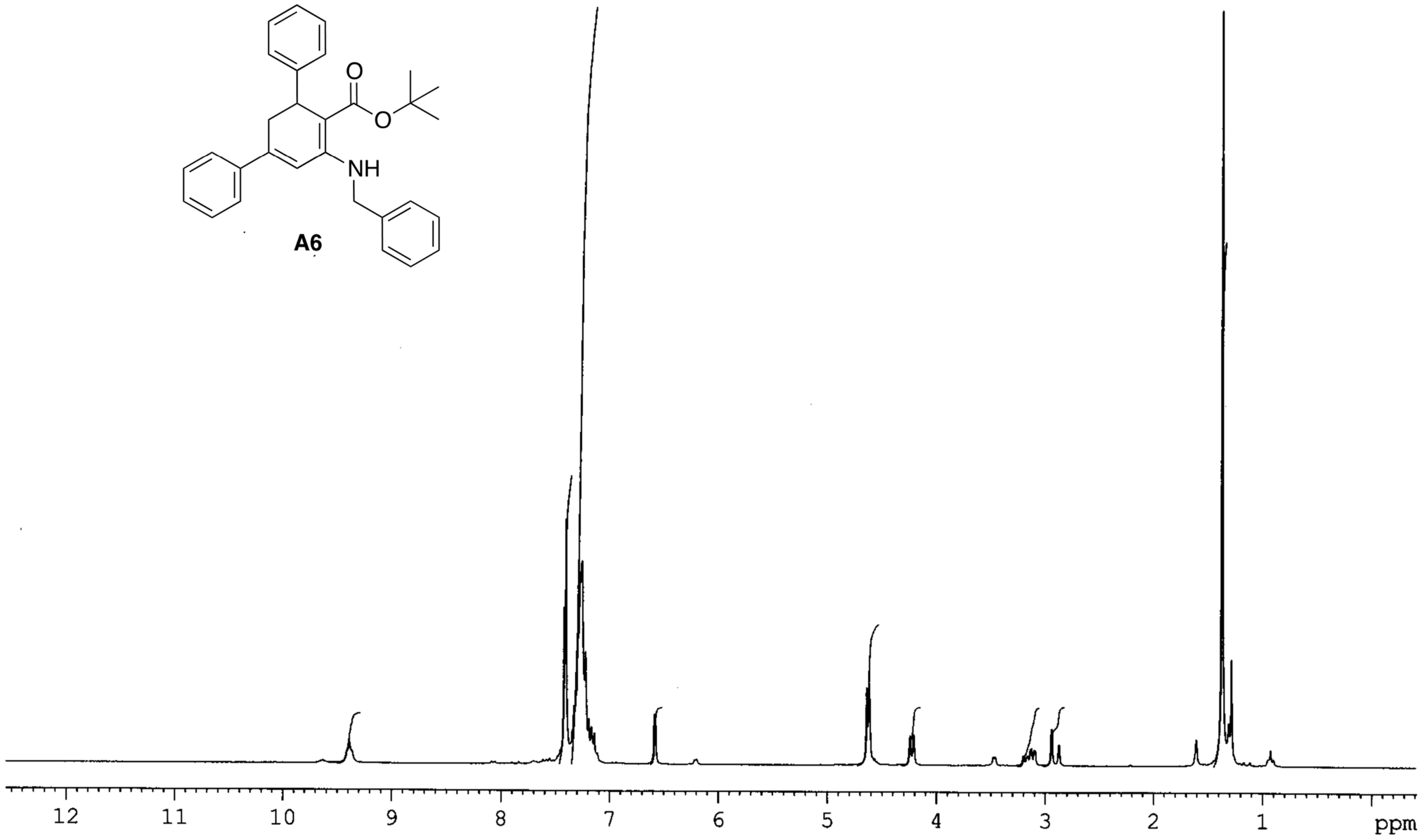
O
O
NH
A6
12
11
10
9
8
7
6
5
4
3
2
1
ppm

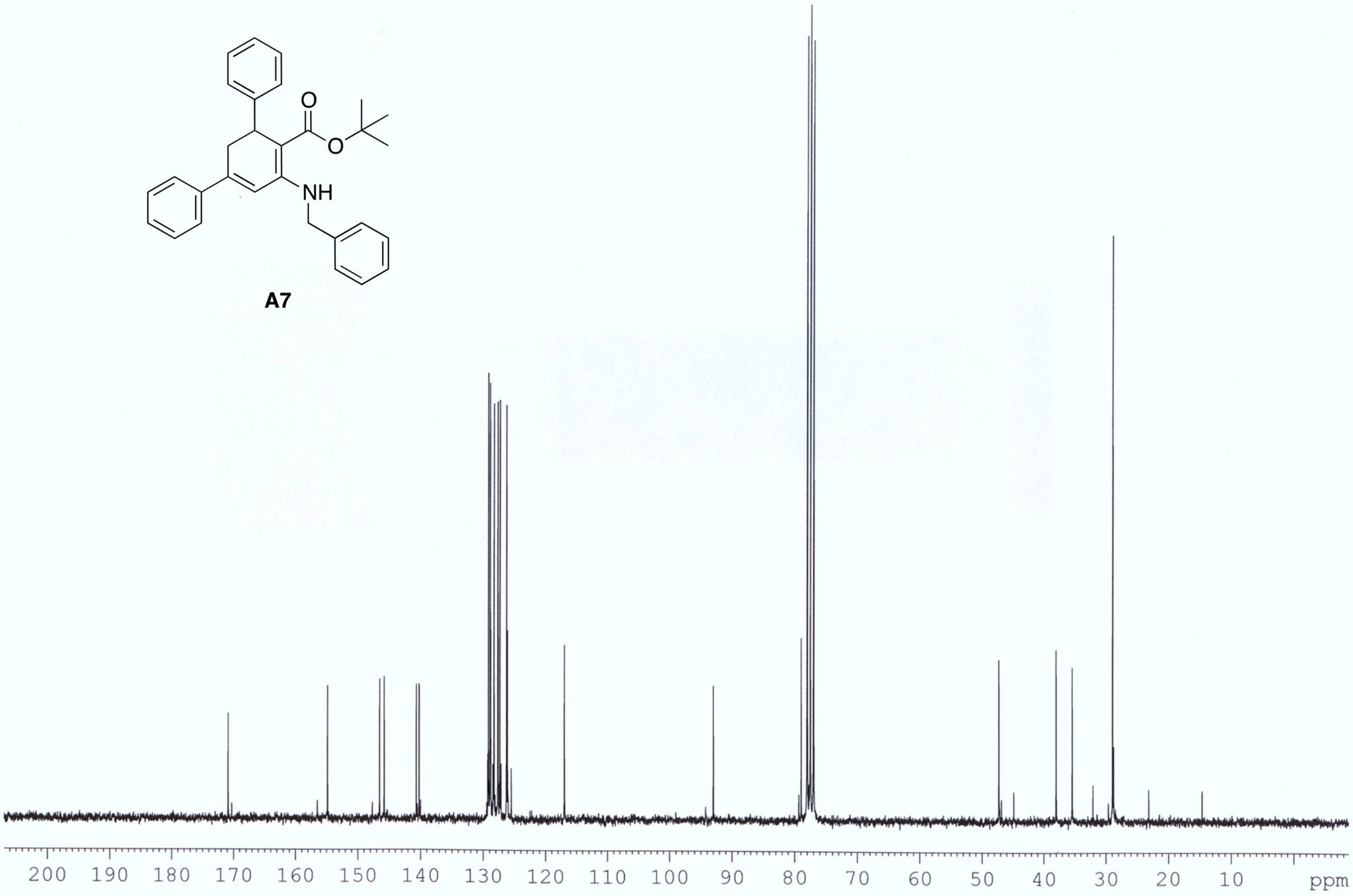
O
O
NH

A7

200 190 180 170 160 150 140 130 120 110 100 90 80 70 60 50 40 30 20 10 ppm

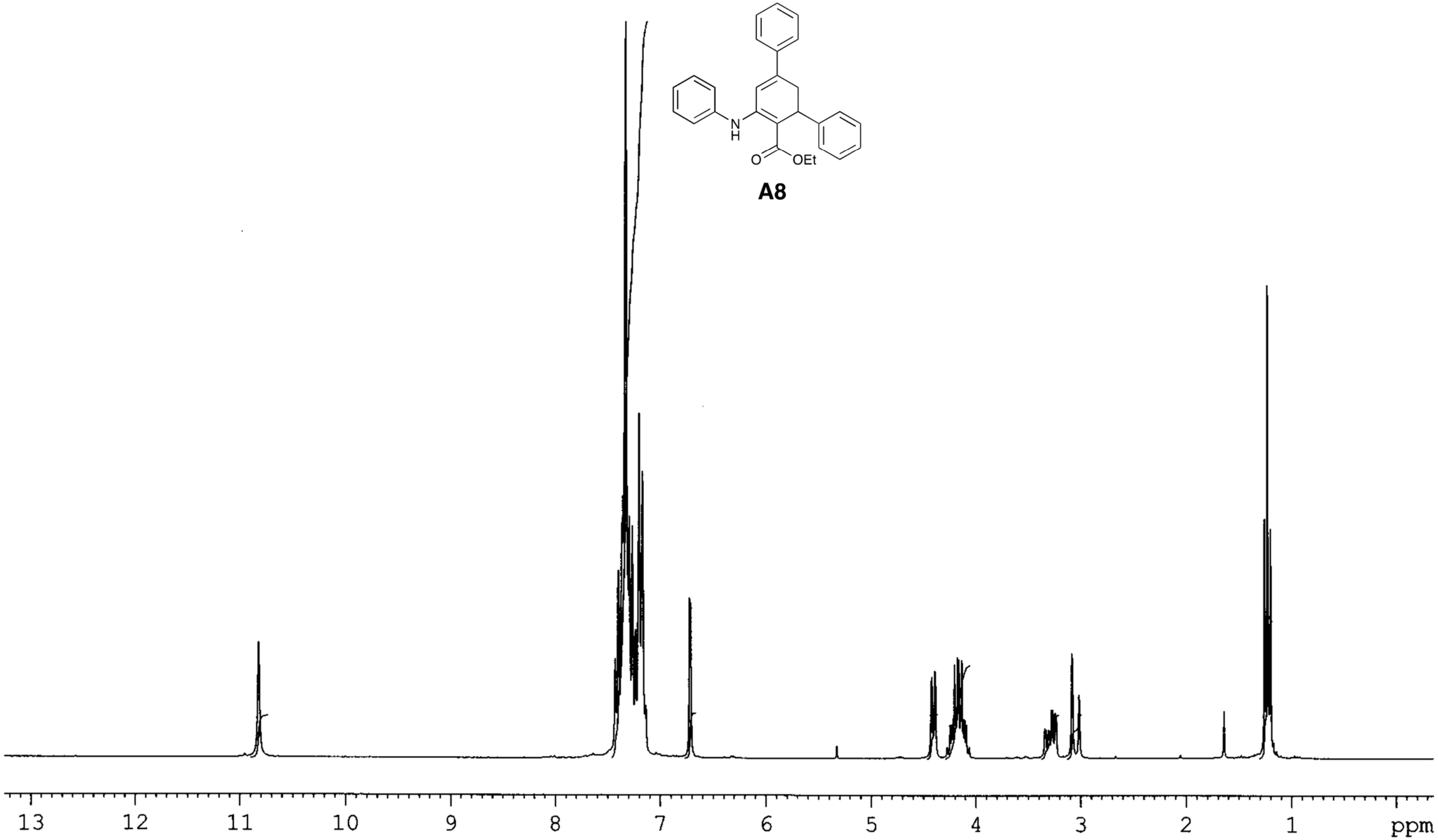
H
N
O
OEt
A8
13
12
11
10
9
8
7
6
5
4
3
2
1
ppm

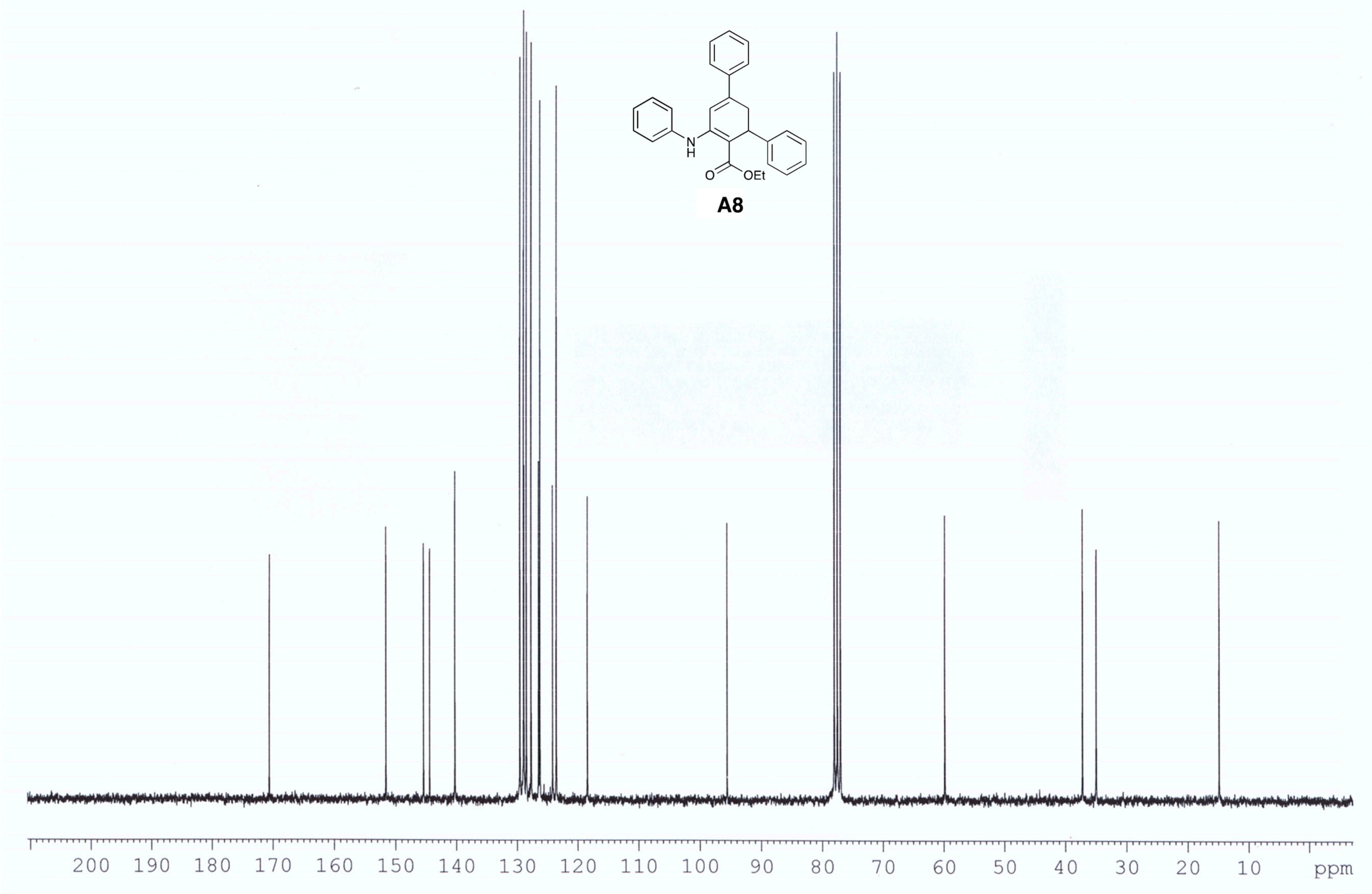
N
H
O
OEt
A8
200 190 180 170 160 150 140 130 120 110 100 90 80 70 60 50 40 30 20 10 ppm

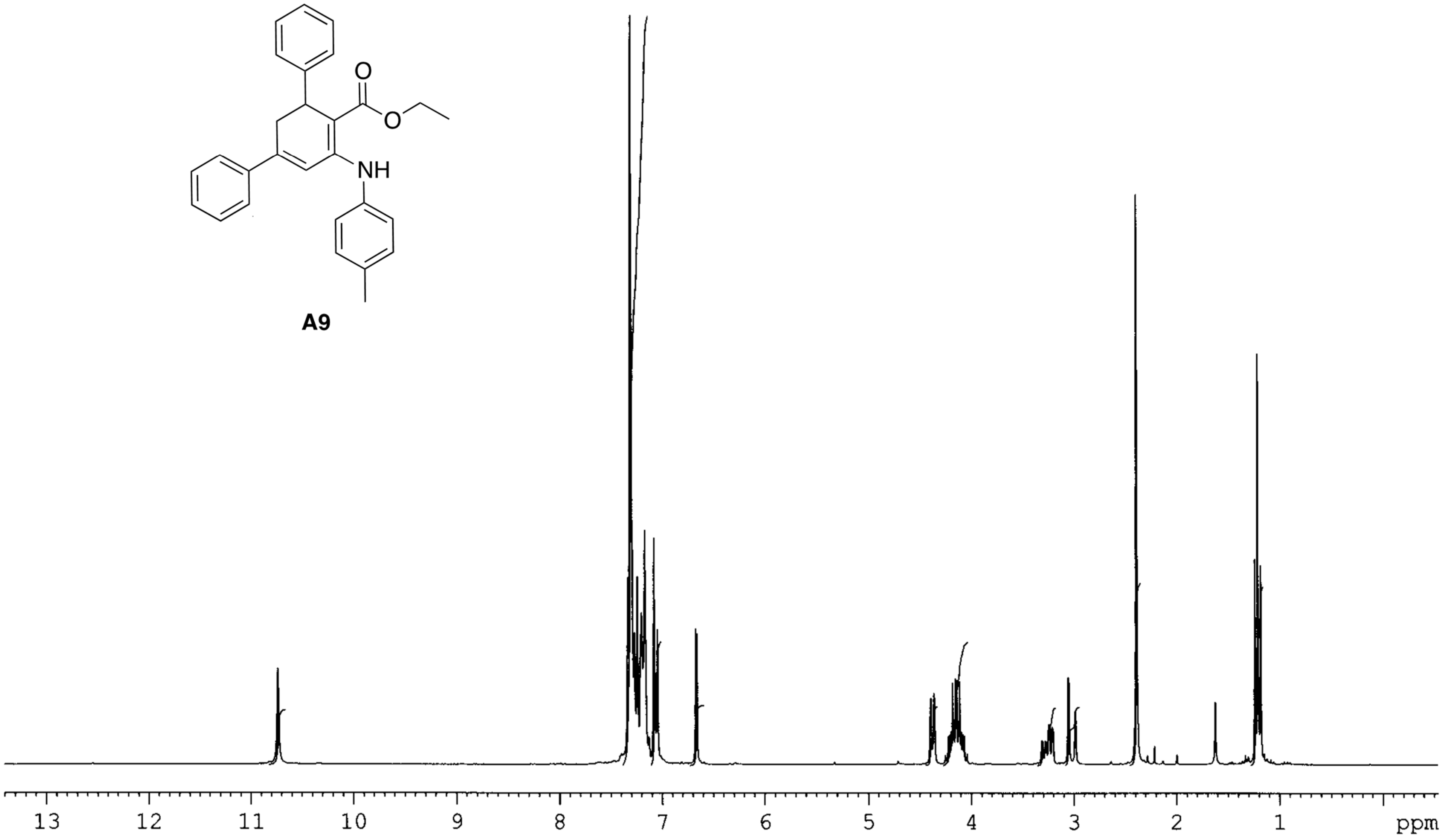
O
O
NH
A9
13
12
11
10
9
8
7
6
5
4
3
2
1
ppm

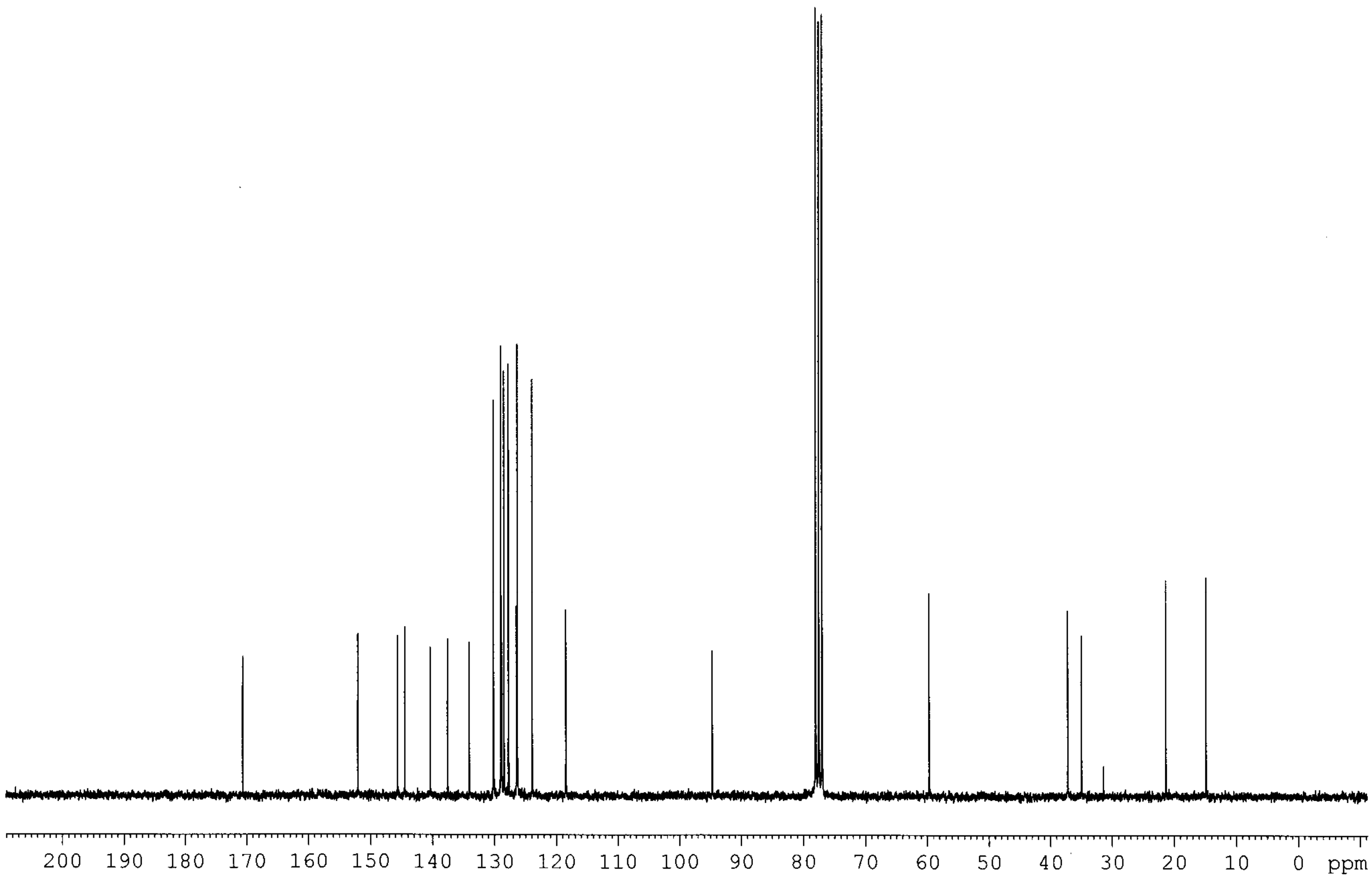

200
190
180
170
160
150
140
130
120
110
100
90
80
70
60
50
40
30
20
10
0
ppm

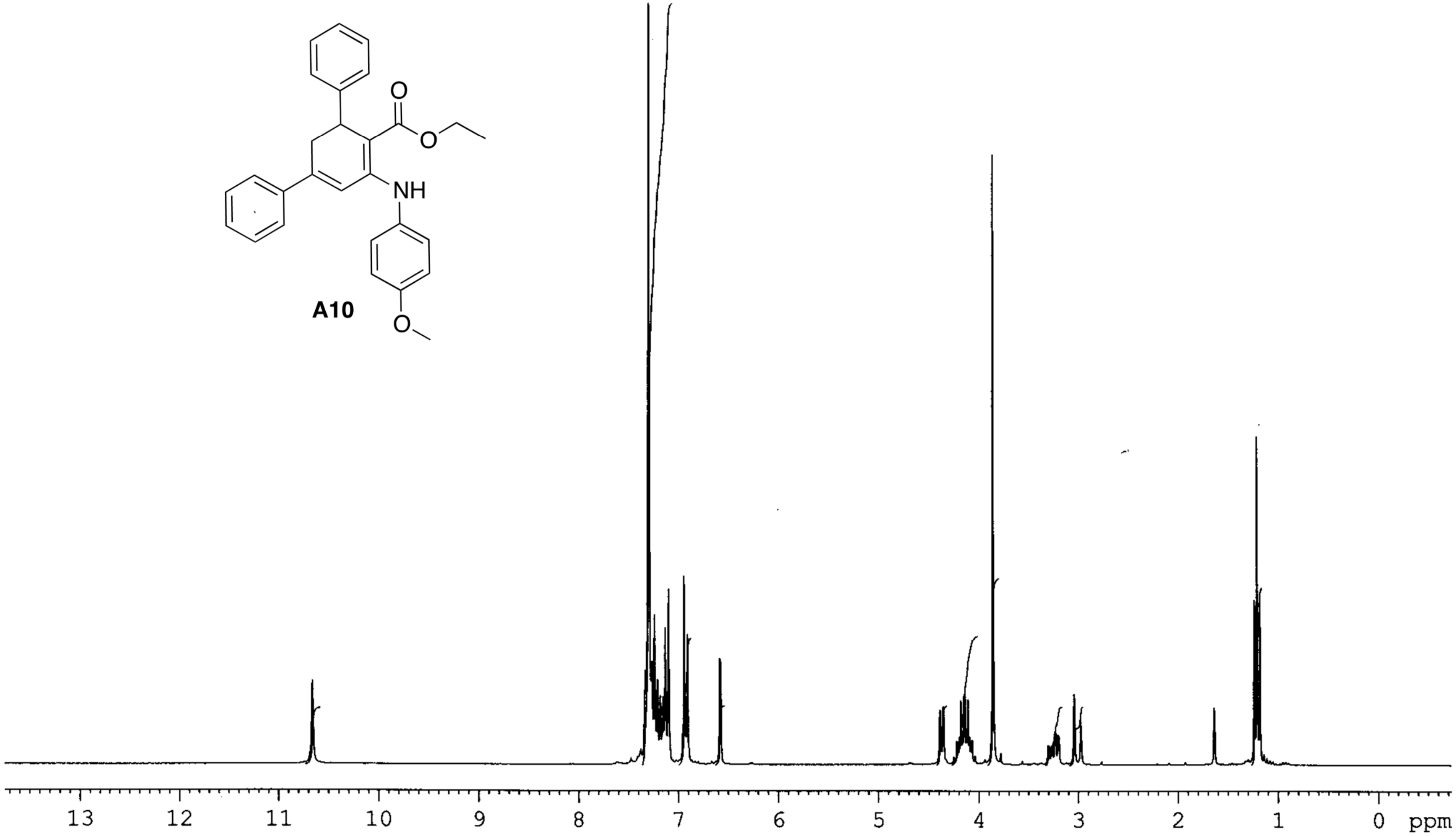

A10

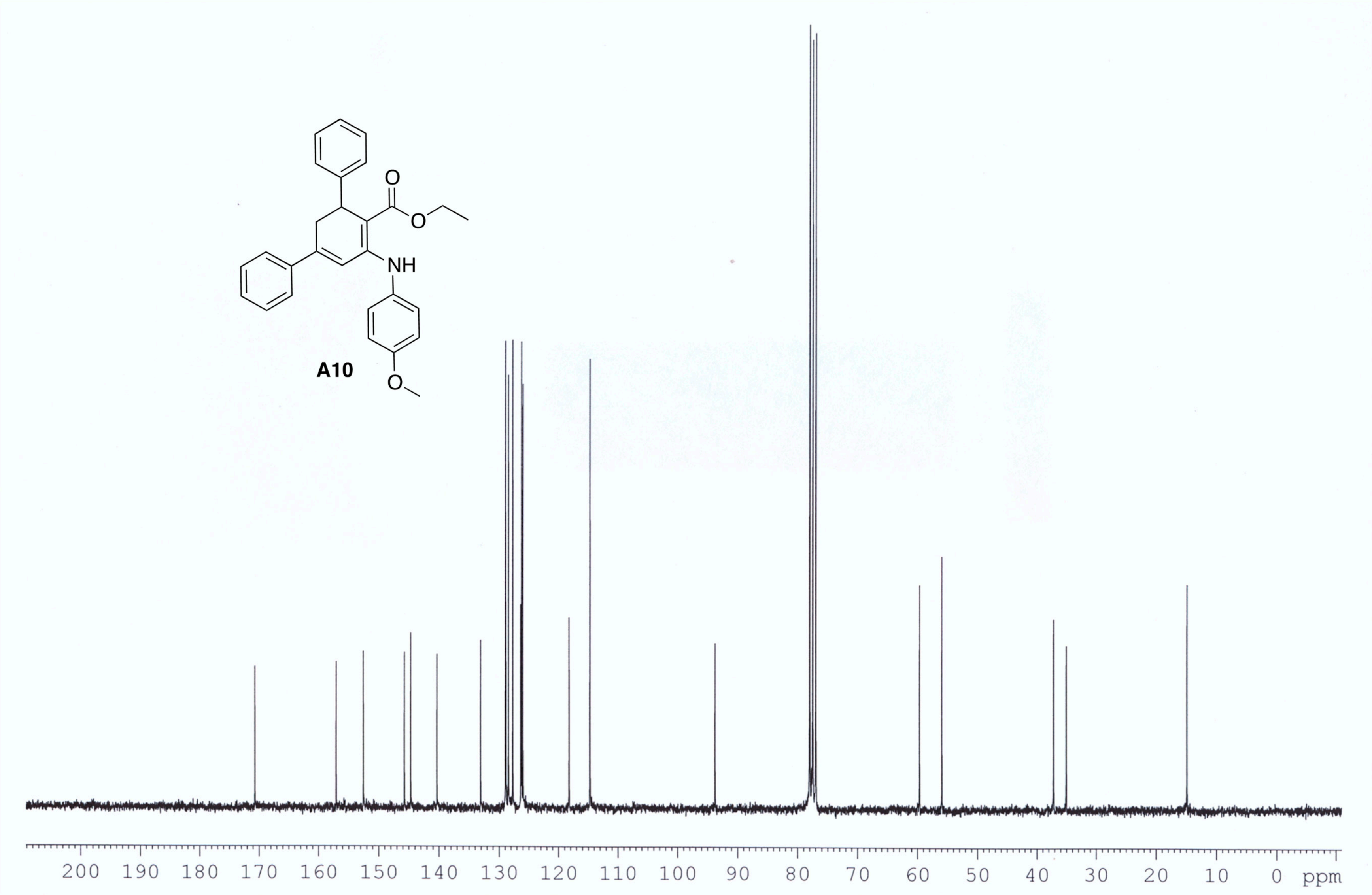
O
O
NH
O
A10
200 190 180 170 160 150 140 130 120 110 100 90 80 70 60 50 40 30 20 10 0 ppm

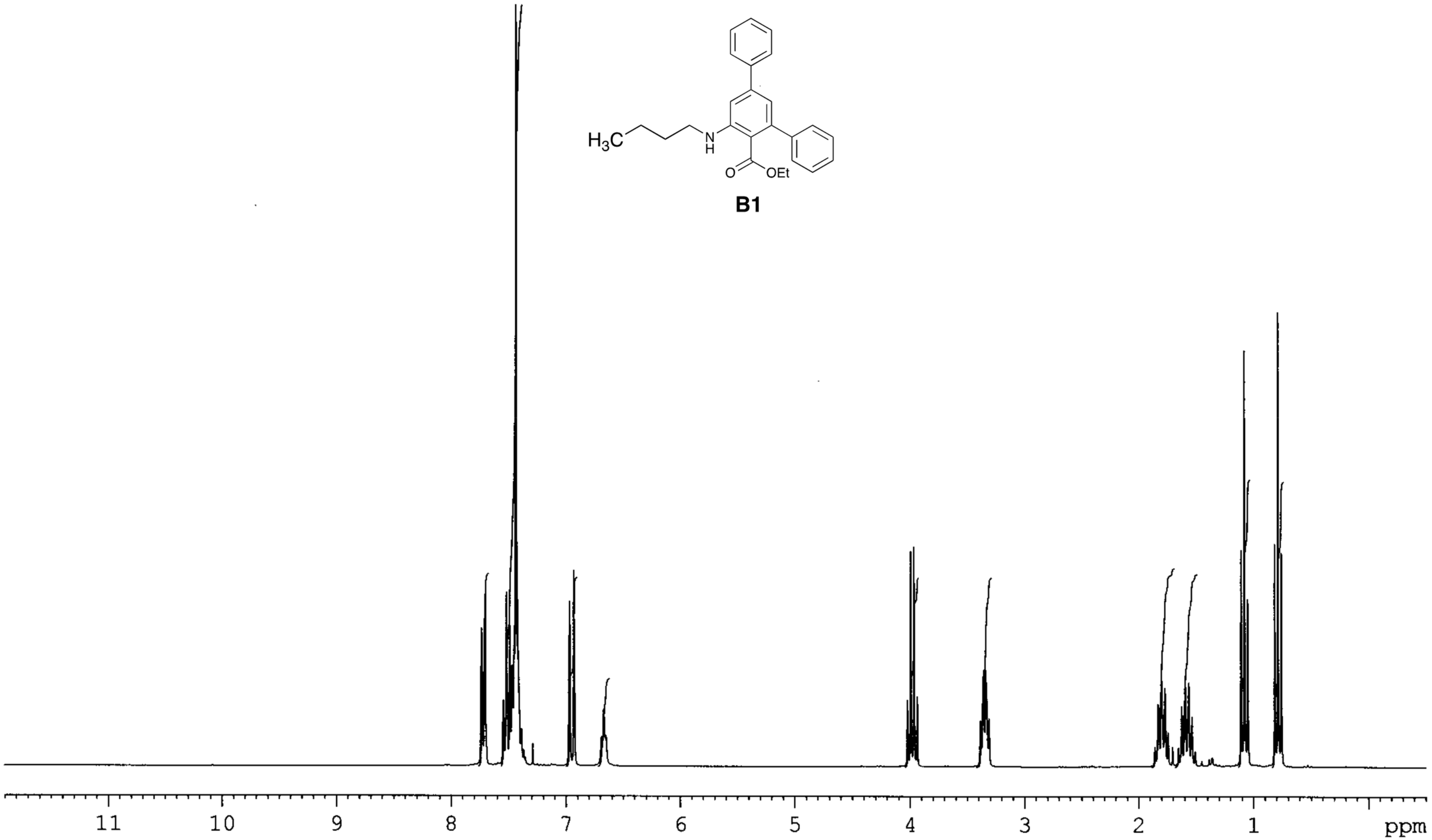
H3C
N
H
O
OEt
B1
11
10
9
8
7
6
5
4
3
2
1
ppm

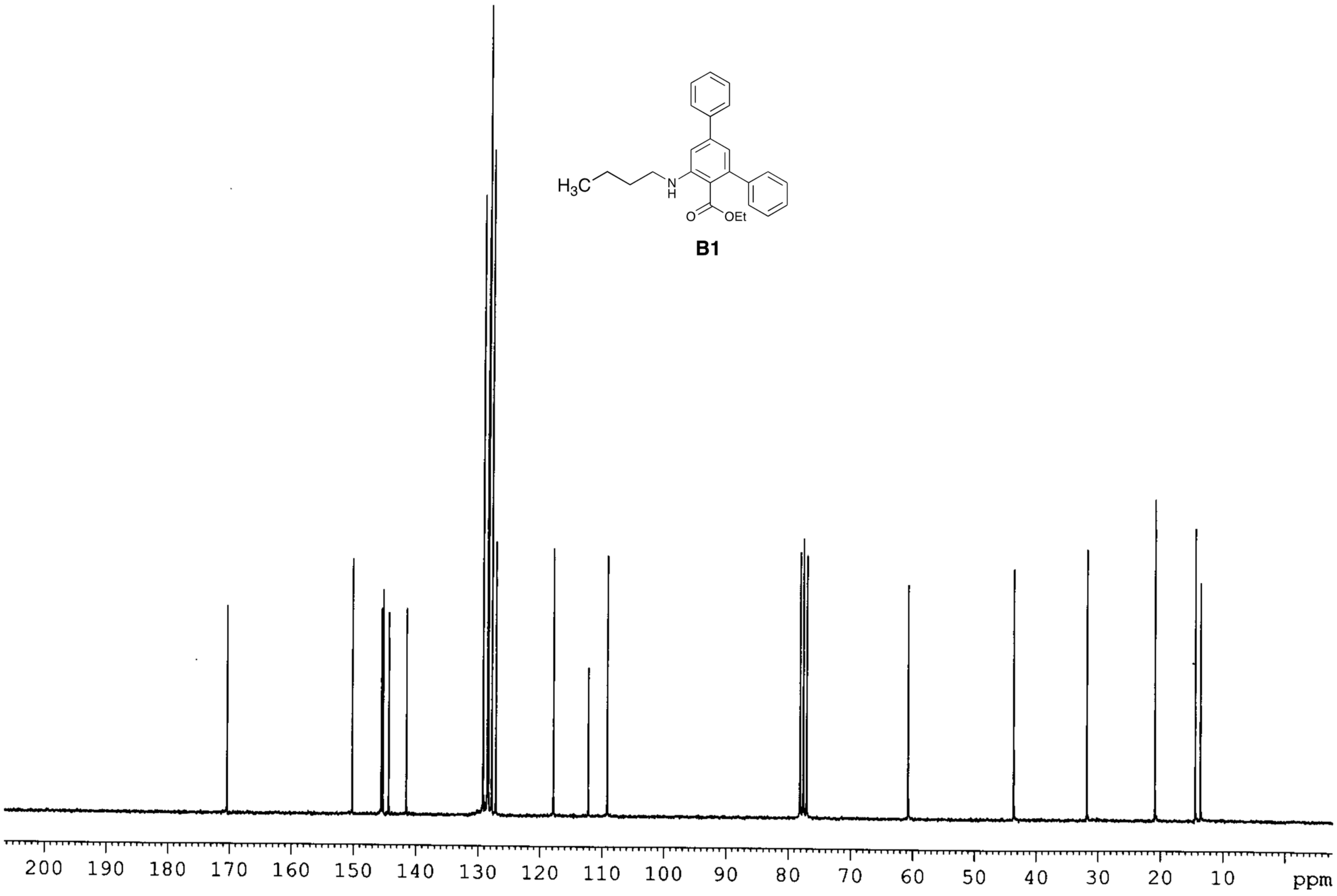
H3C
N
H
O
OEt
B1
200 190 180 170 160 150 140 130 120 110 100 90 80 70 60 50 40 30 20 10
ppm

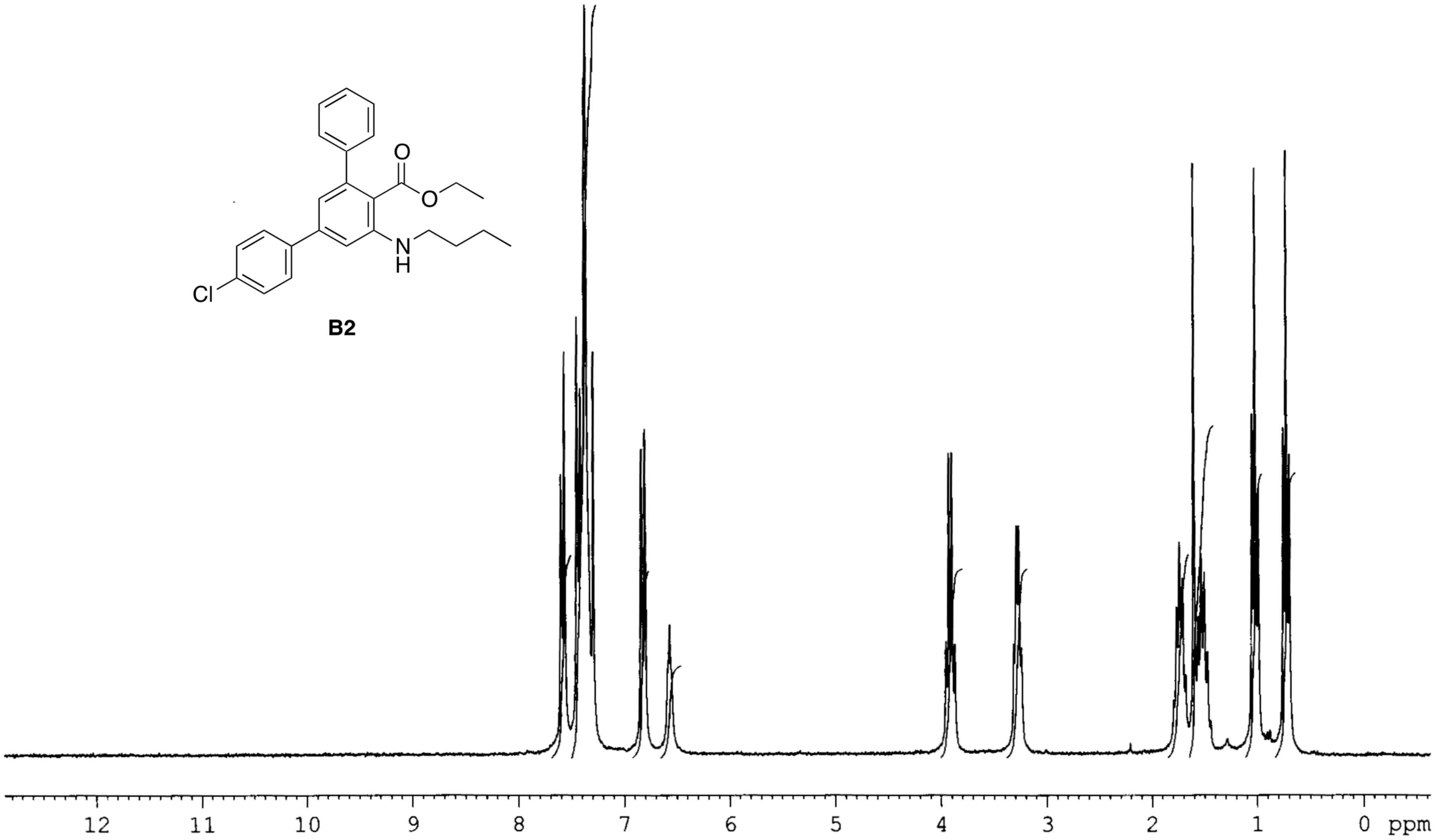
O
O
N
H
Cl
B2
12 11 10 9 8 7 6 5 4 3 2 1 0 ppm

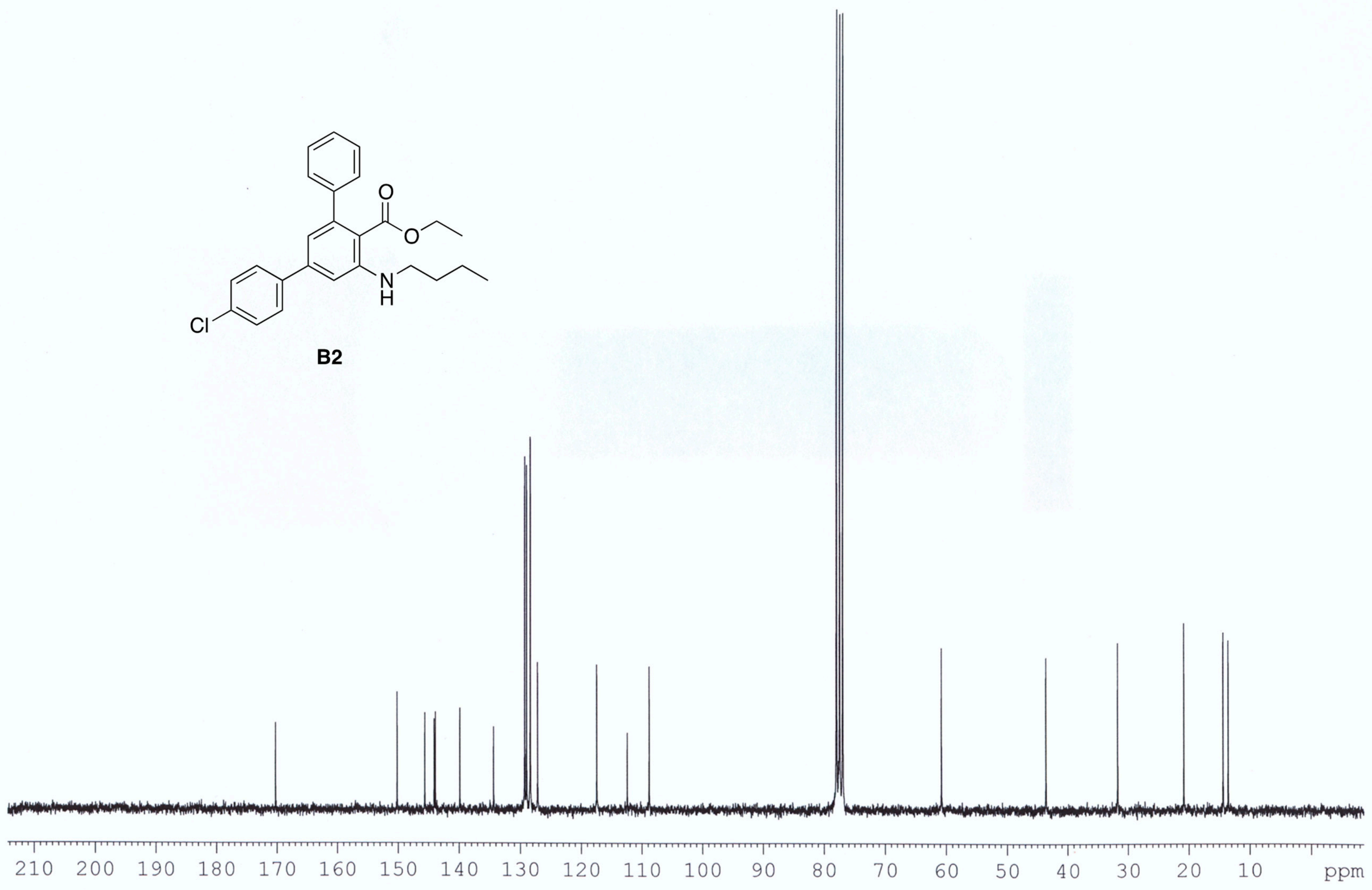
O
O
N
H
Cl
B2
210 200 190 180 170 160 150 140 130 120 110 100 90 80 70 60 50 40 30 20 10 ppm

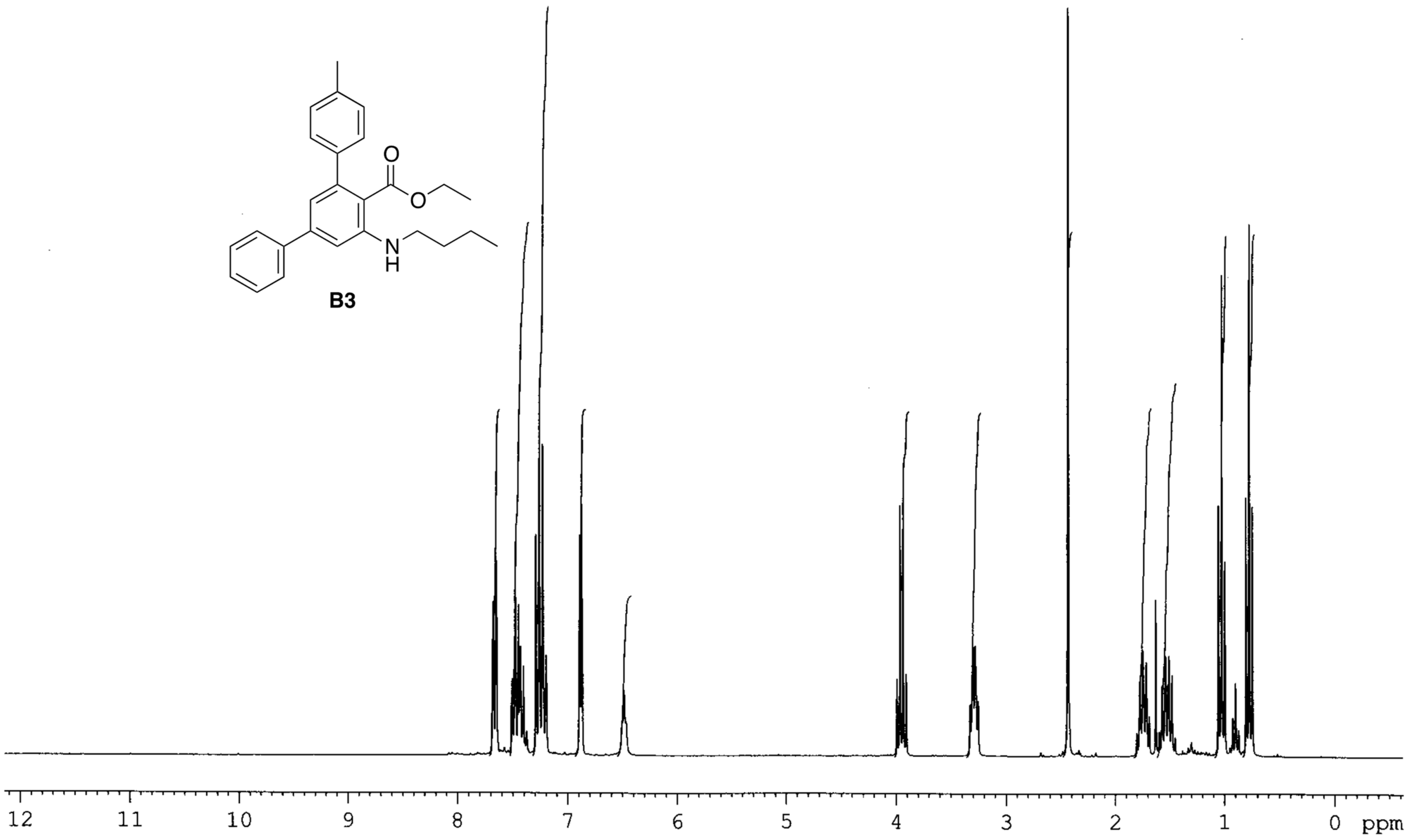

O
O
N
H
B3
12
11
10
9
8
7
6
5
4
3
2
1
0
ppm

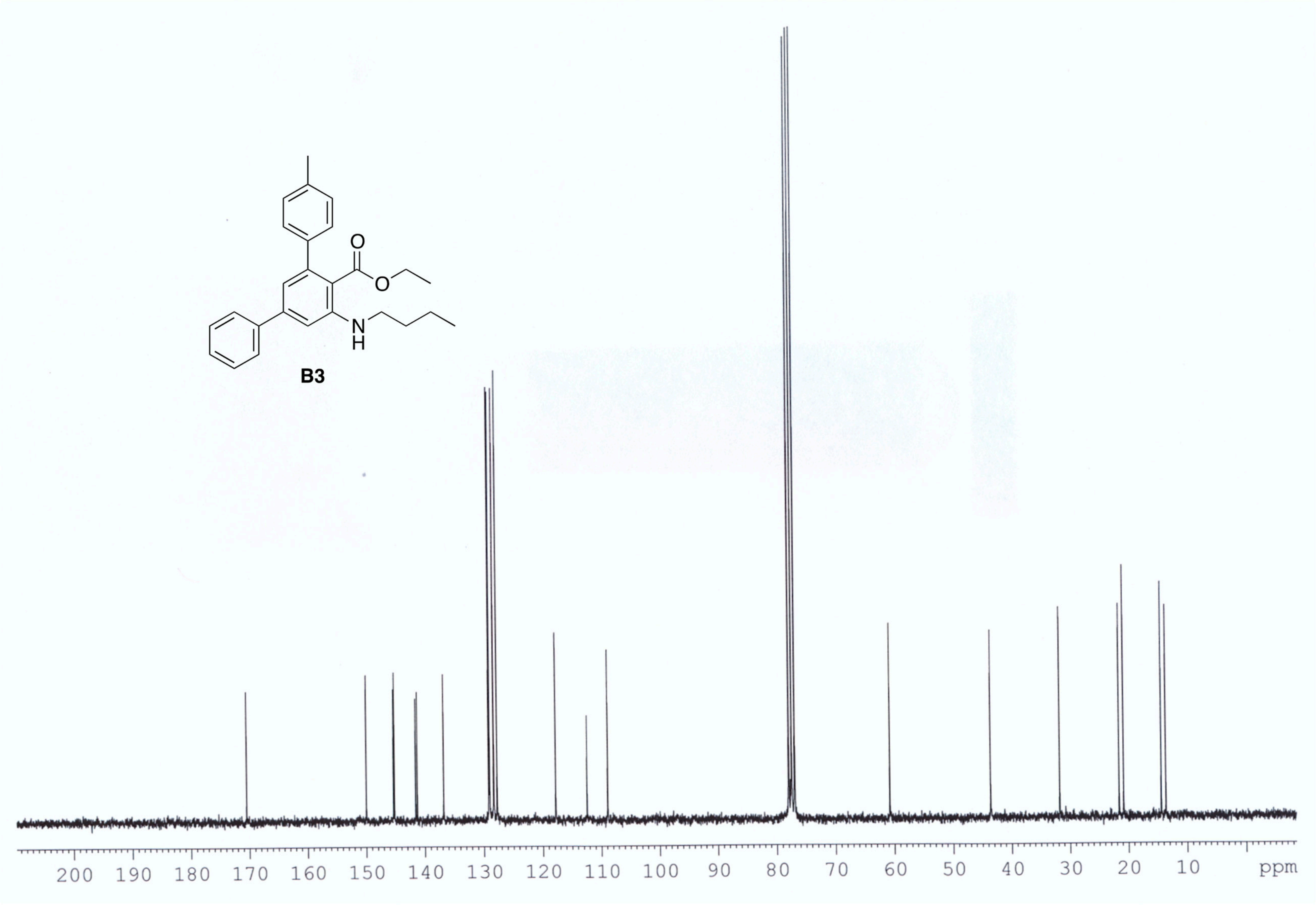
B3
200 190 180 170 160 150 140 130 120 110 100 90 80 70 60 50 40 30 20 10 ppm

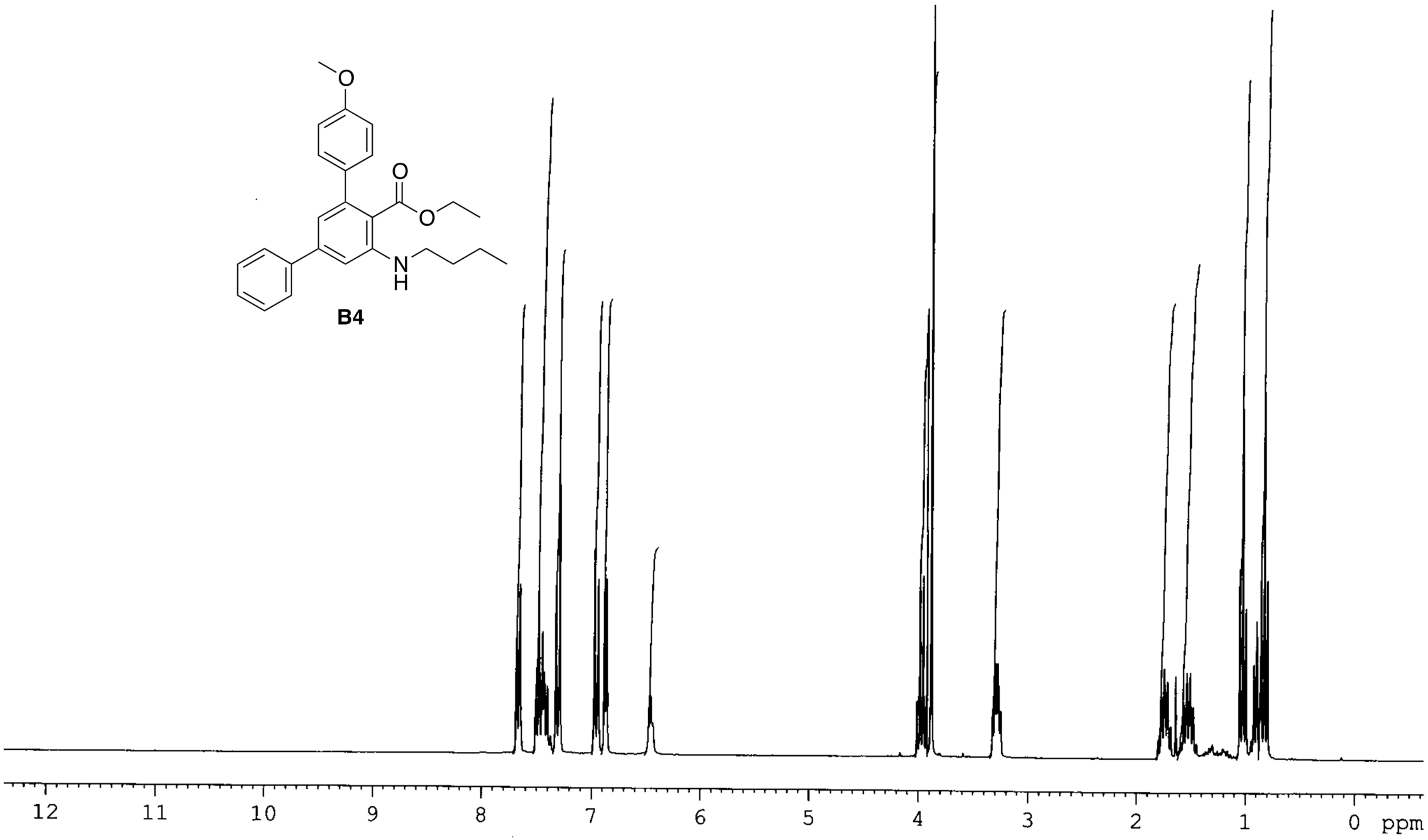

B4

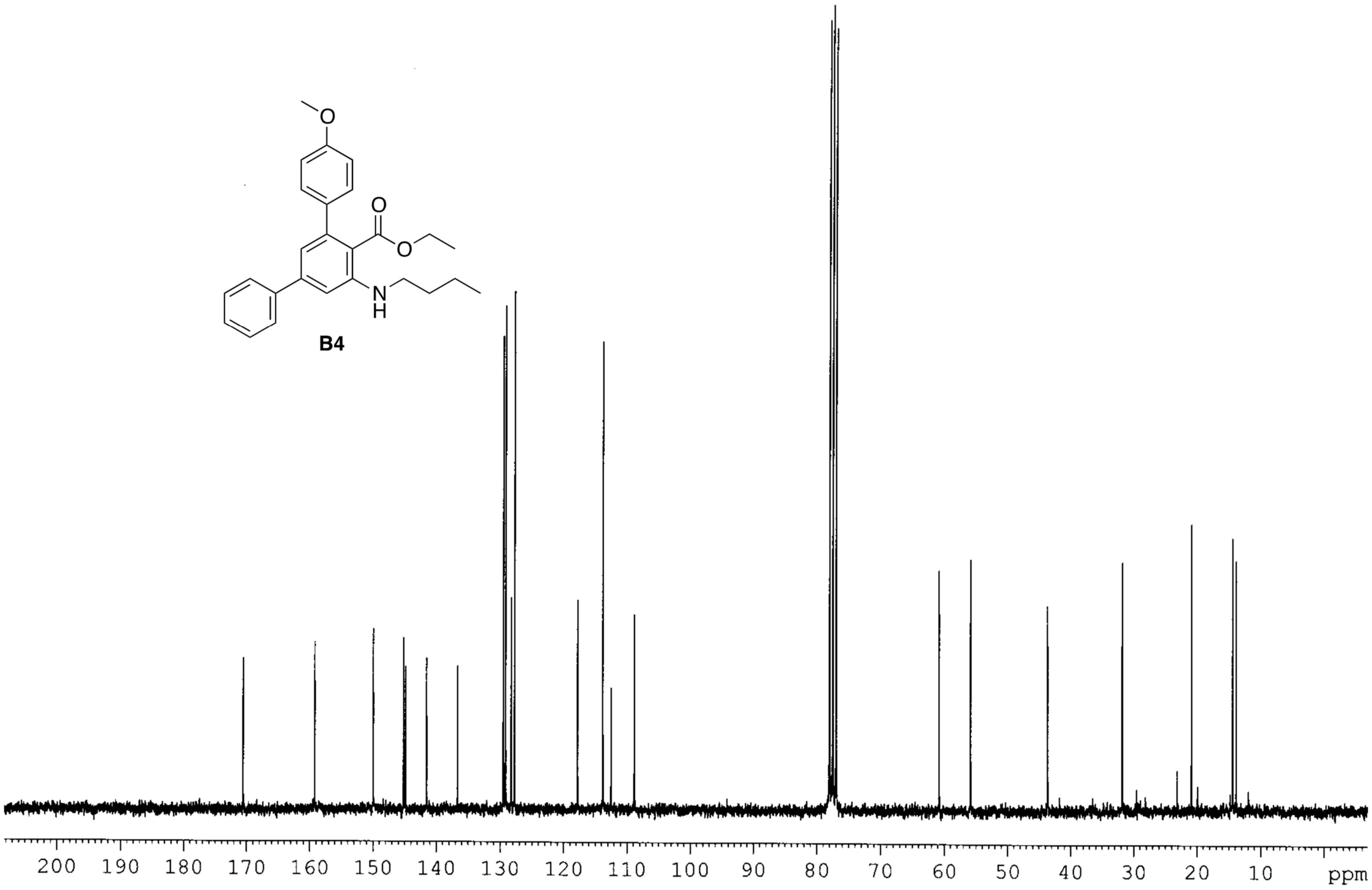
O
O
O
N
H
B4
200 190 180 170 160 150 140 130 120 110 100 90 80 70 60 50 40 30 20 10 ppm

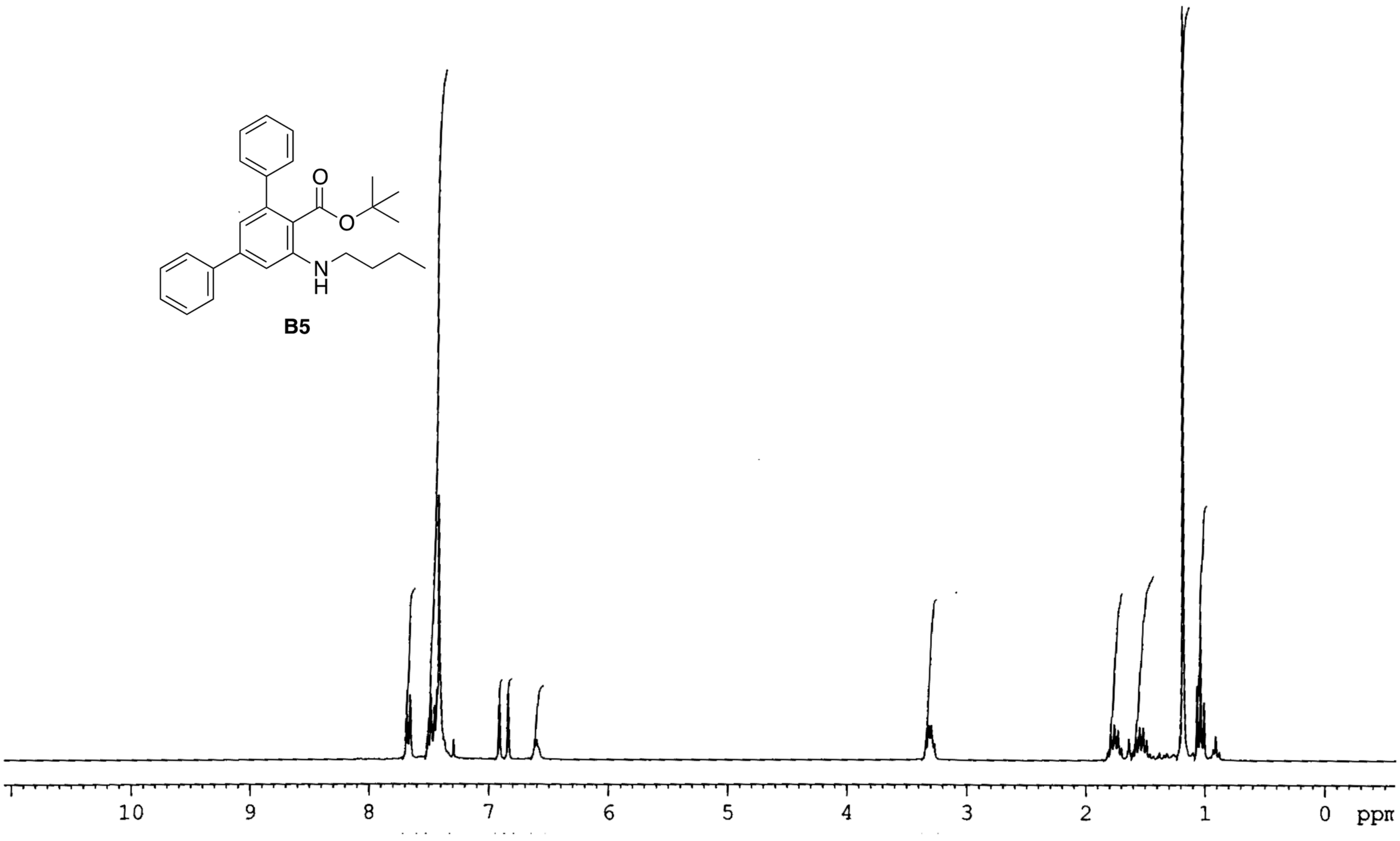
O
O
N
H
B5
10
9
8
7
6
5
4
3
2
1
0
ppm

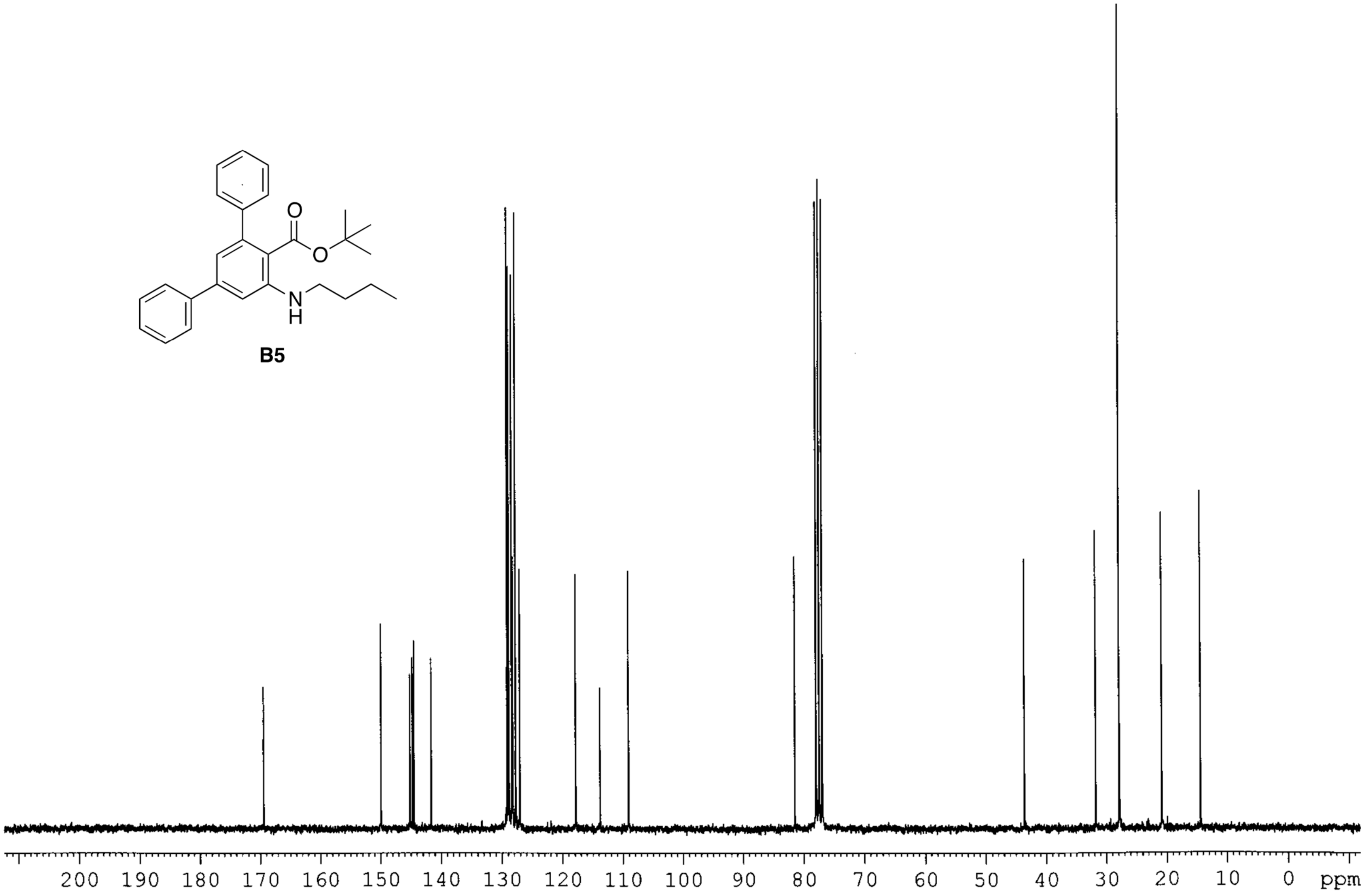
O
O
N
H
B5
200 190 180 170 160 150 140 130 120 110 100 90 80 70 60 50 40 30 20 10 0 ppm

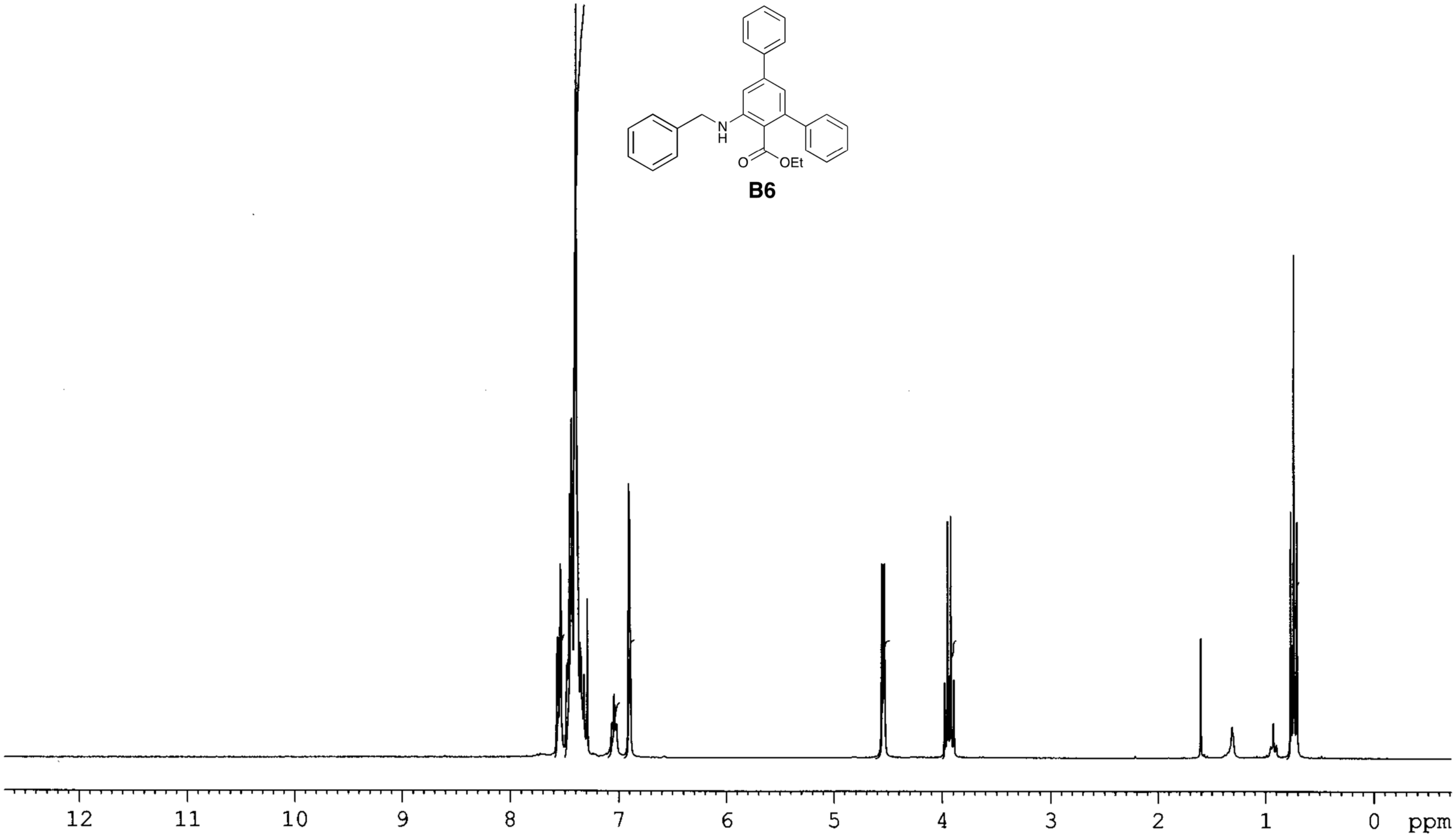

N
H
O
OEt
B6
12
11
10
9
8
7
6
5
4
3
2
1
0
ppm

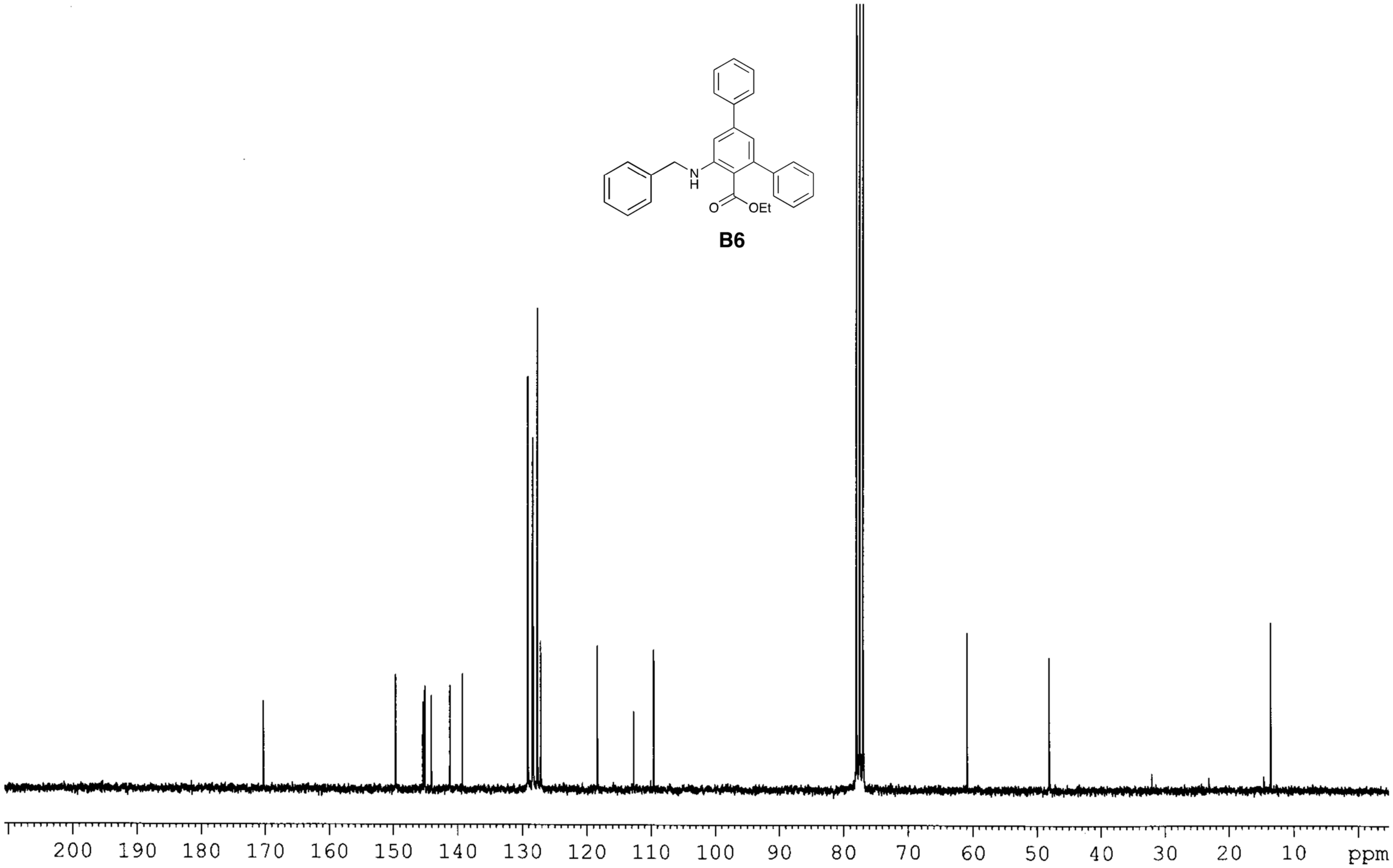
N
H
O
OEt
B6
200 190 180 170 160 150 140 130 120 110 100 90 80 70 60 50 40 30 20 10 ppm

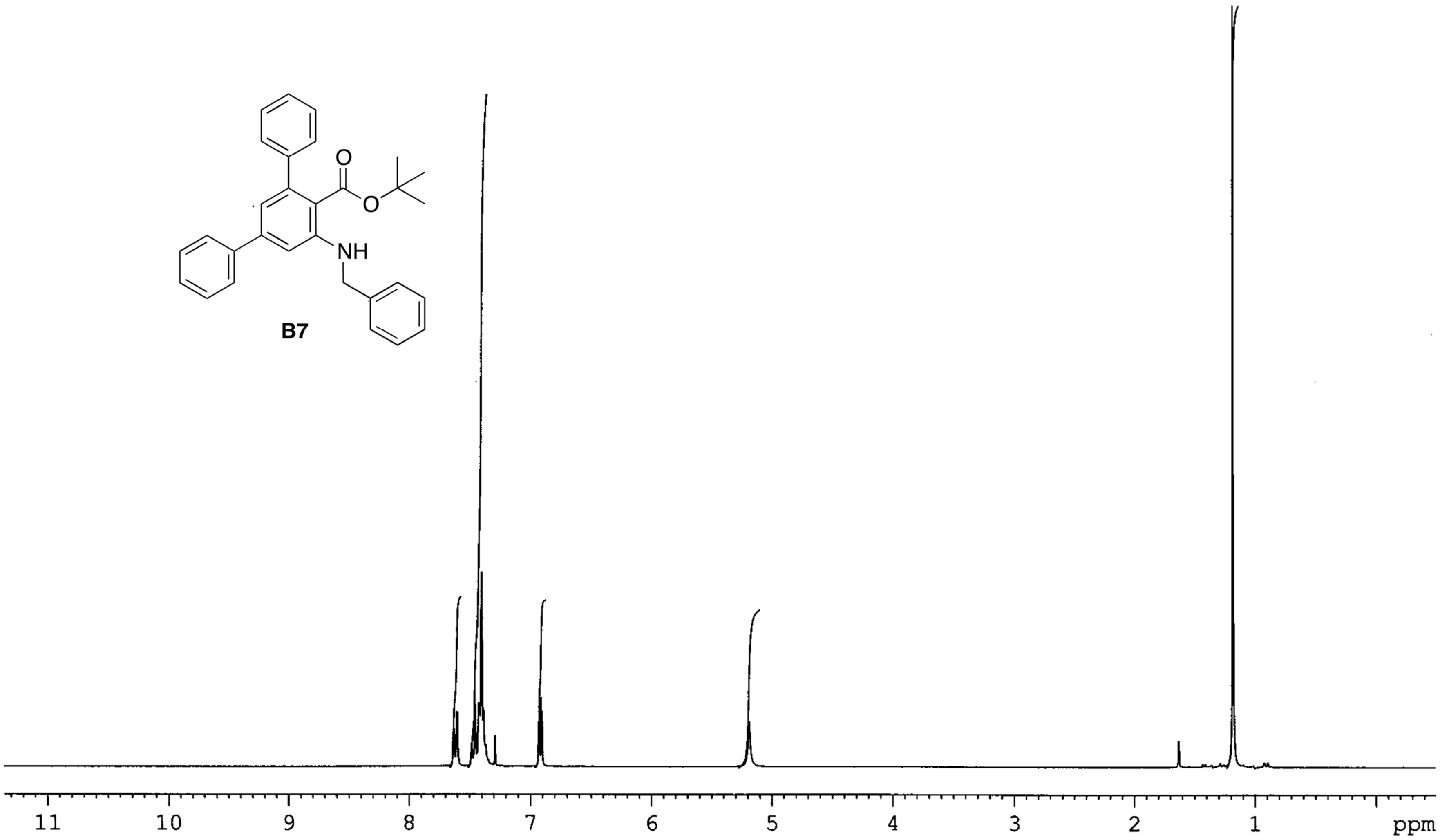
NH
O
O
B7
11
10
9
8
7
6
5
4
3
2
1
ppm

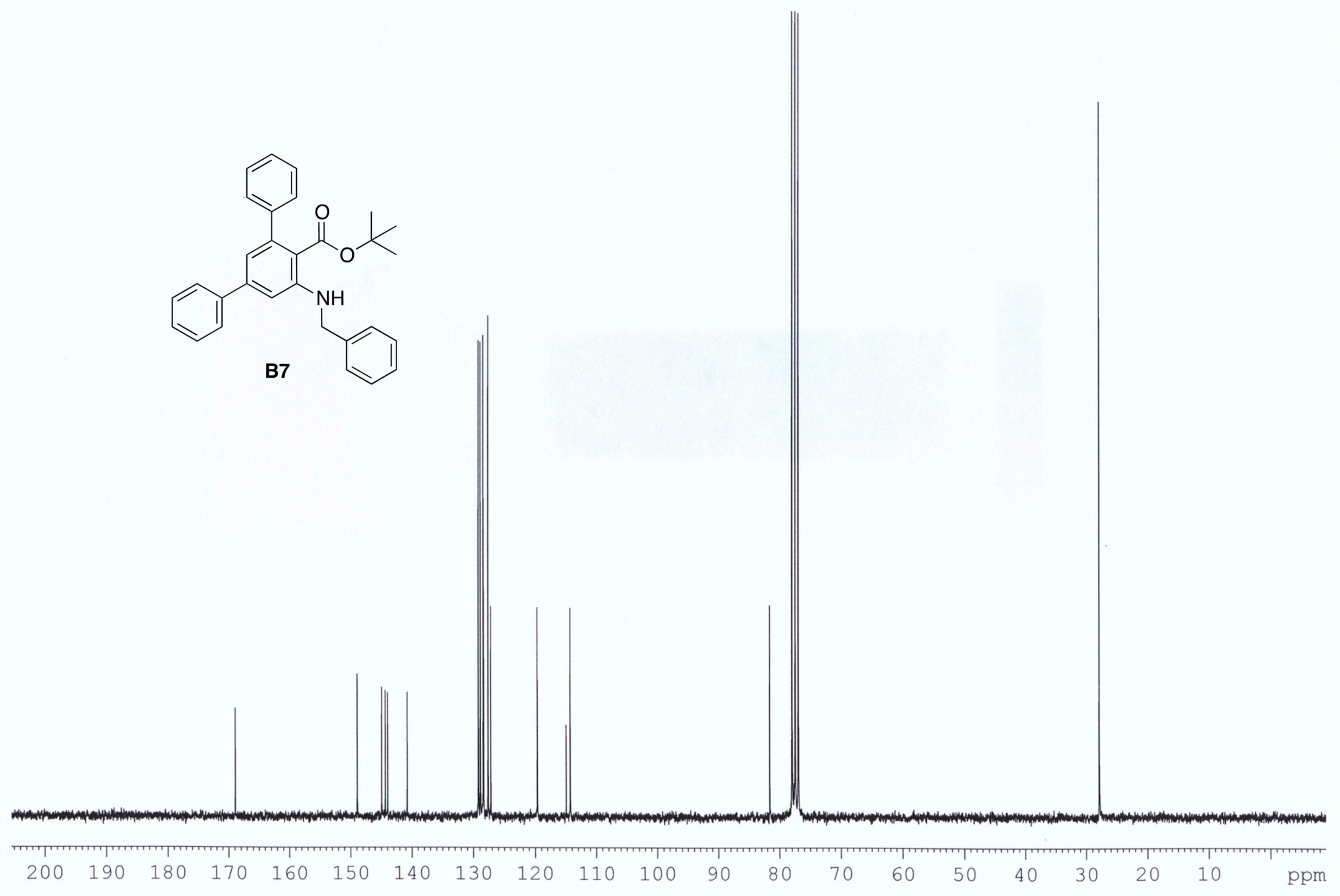
O
O
NH
200 190 180 170 160 150 140 130 120 110 100 90 80 70 60 50 40 30 20 10 ppm

B7

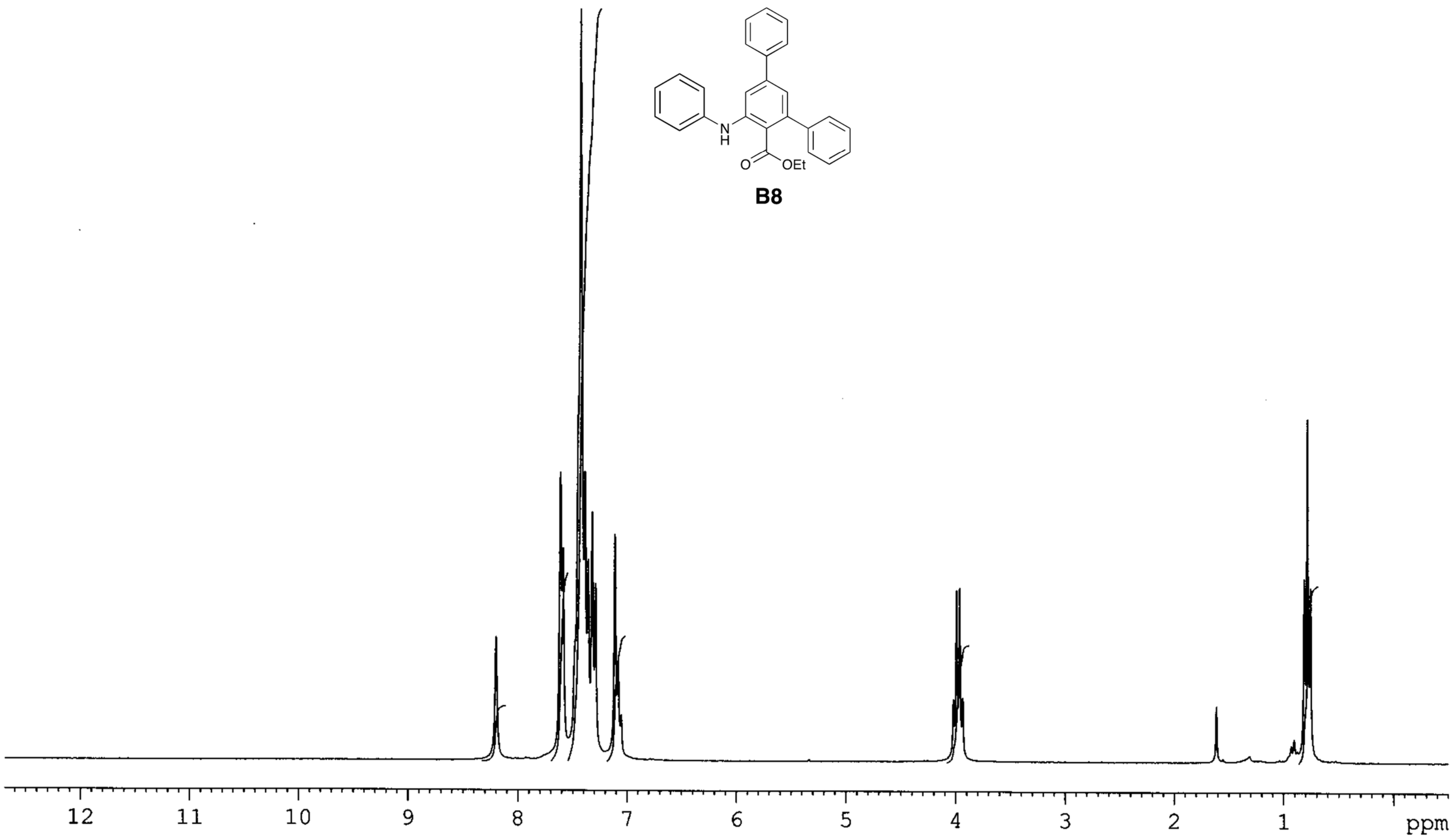

N
H
O
OEt
B8
12
11
10
9
8
7
6
5
4
3
2
1
ppm

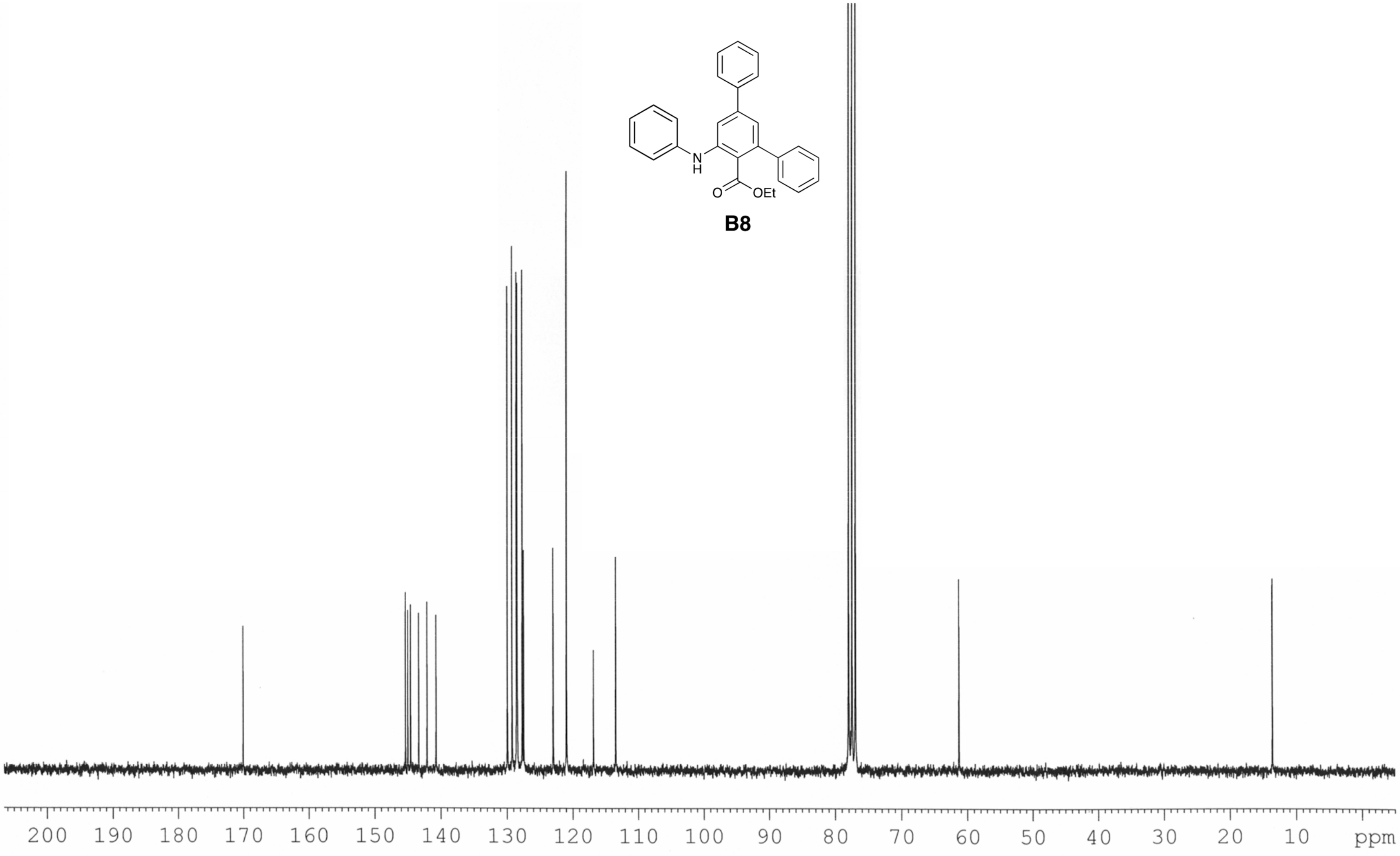
N
H
O
OEt
B8
200 190 180 170 160 150 140 130 120 110 100 90 80 70 60 50 40 30 20 10 ppm

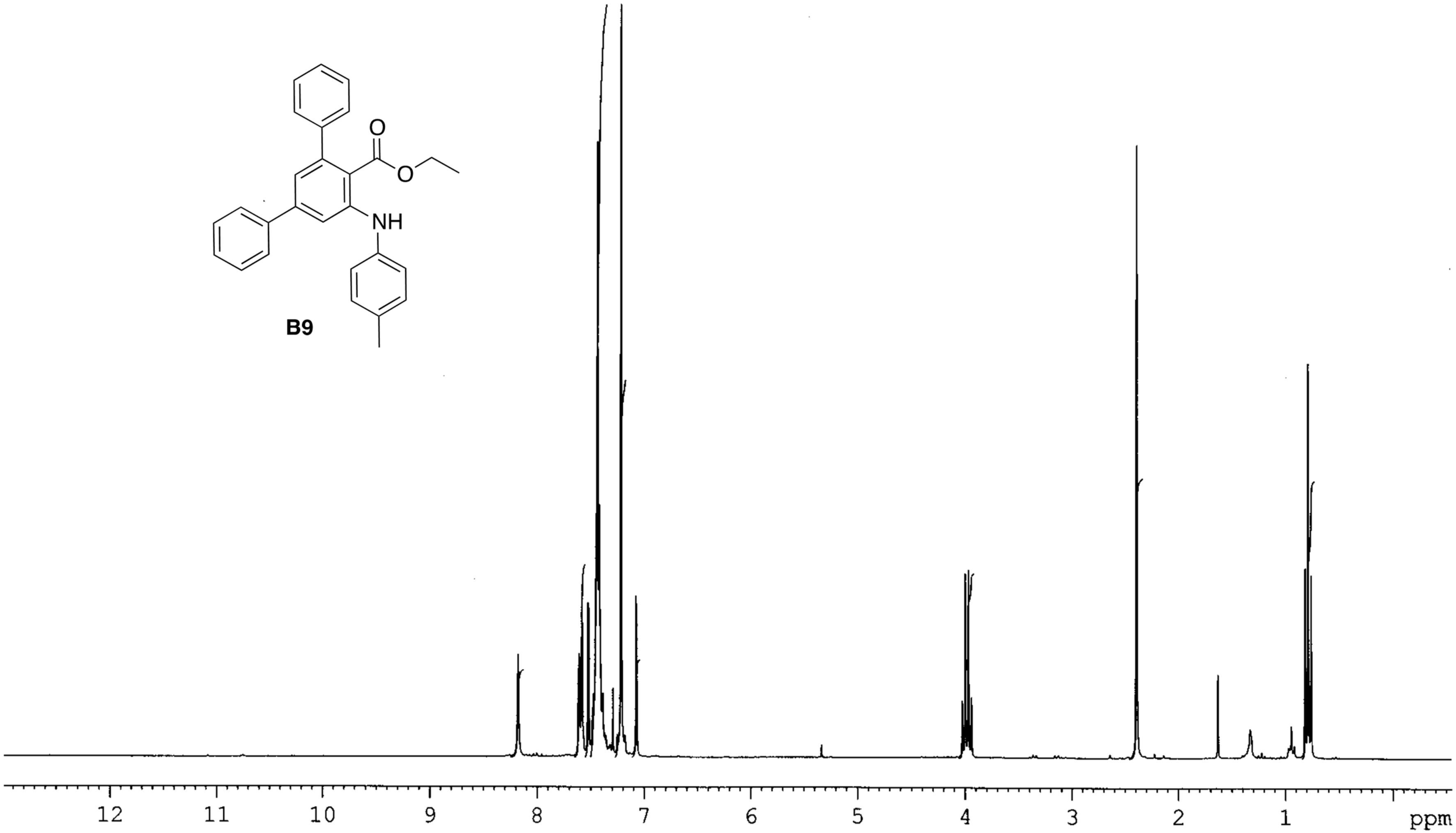

O
O
NH
B9
12
11
10
9
8
7
6
5
4
3
2
1
ppm

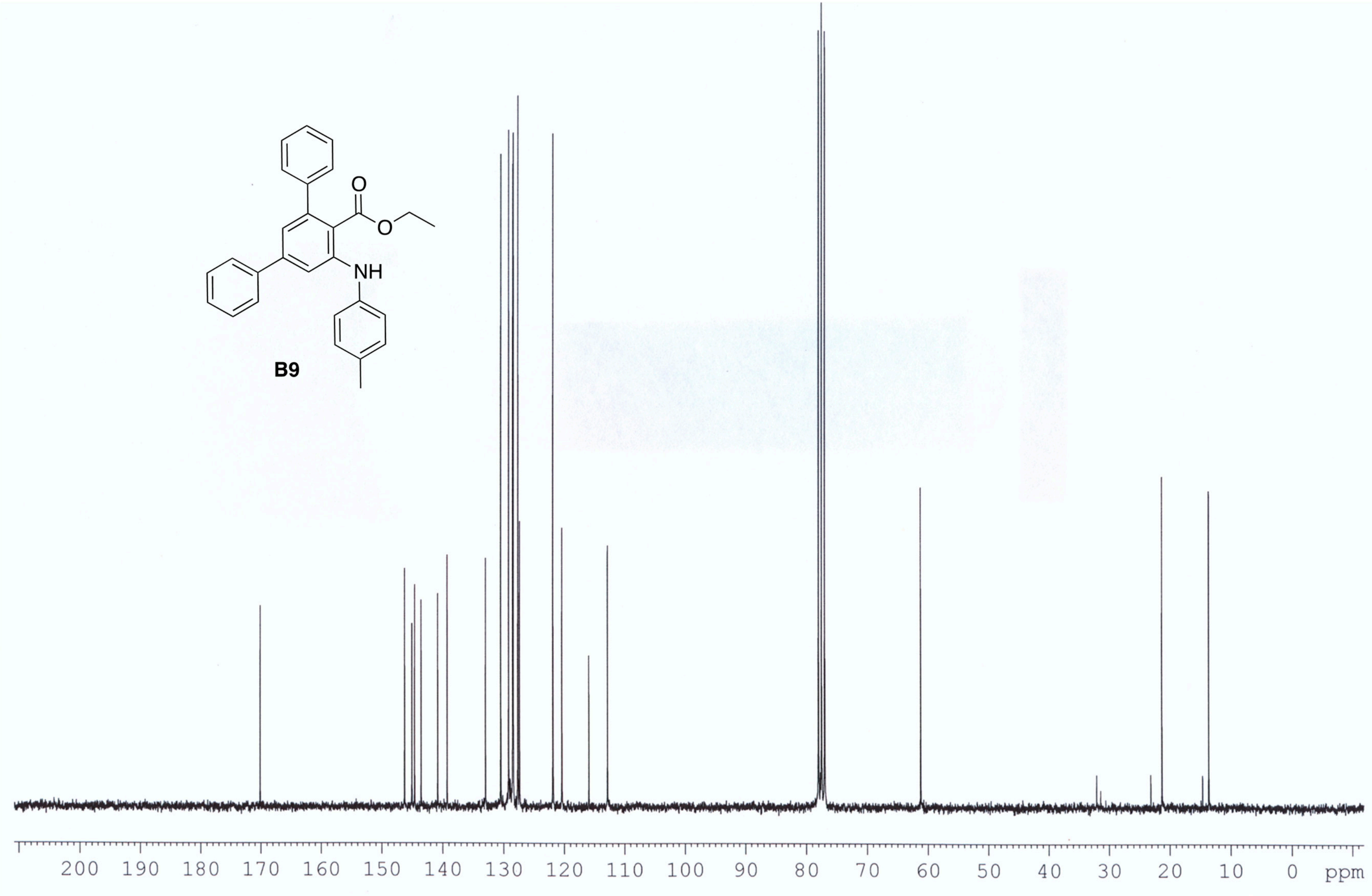
O
O
NH
B9
200 190 180 170 160 150 140 130 120 110 100 90 80 70 60 50 40 30 20 10 0 ppm

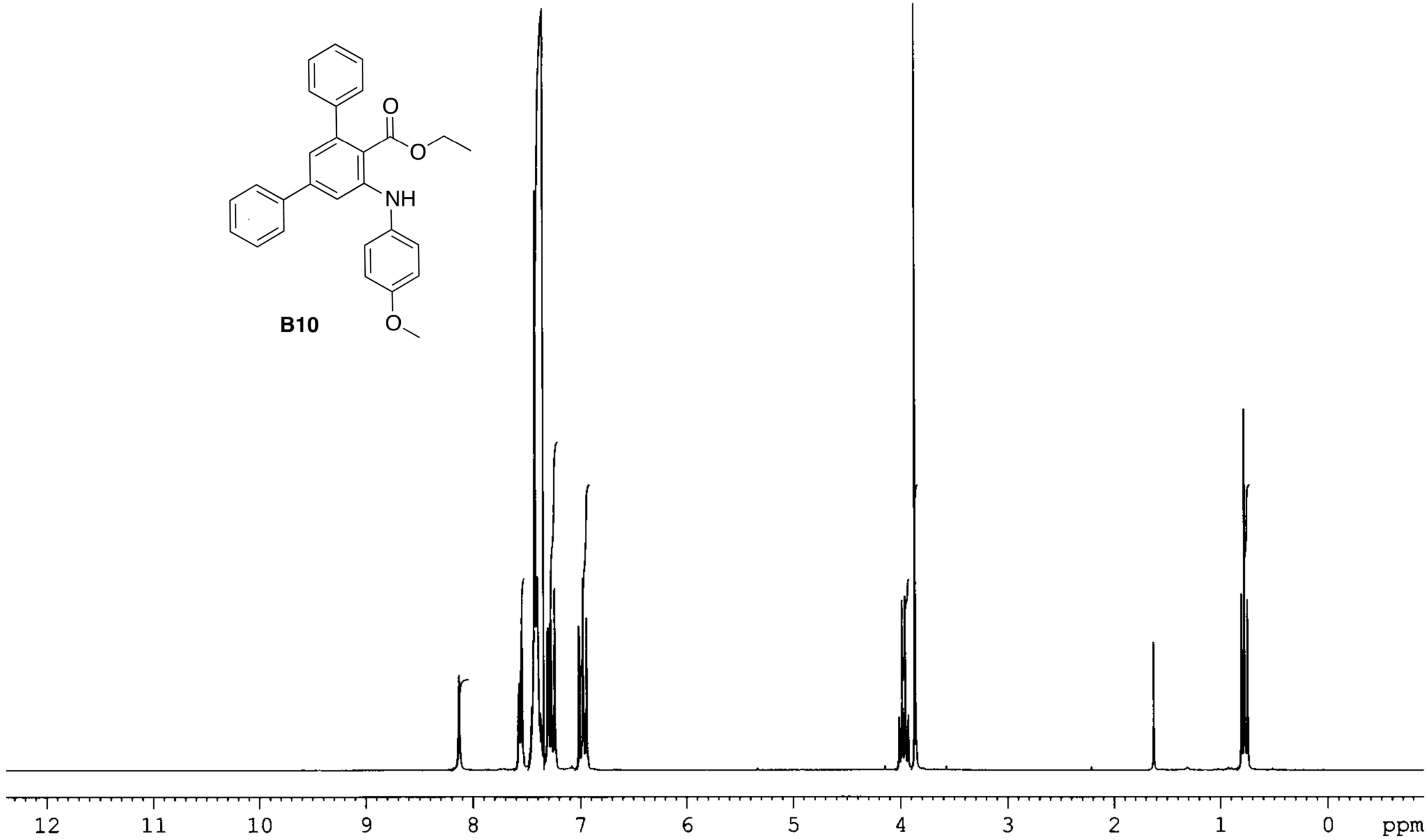

B10
12 11 10 9 8 7 6 5 4 3 2 1 0 ppm

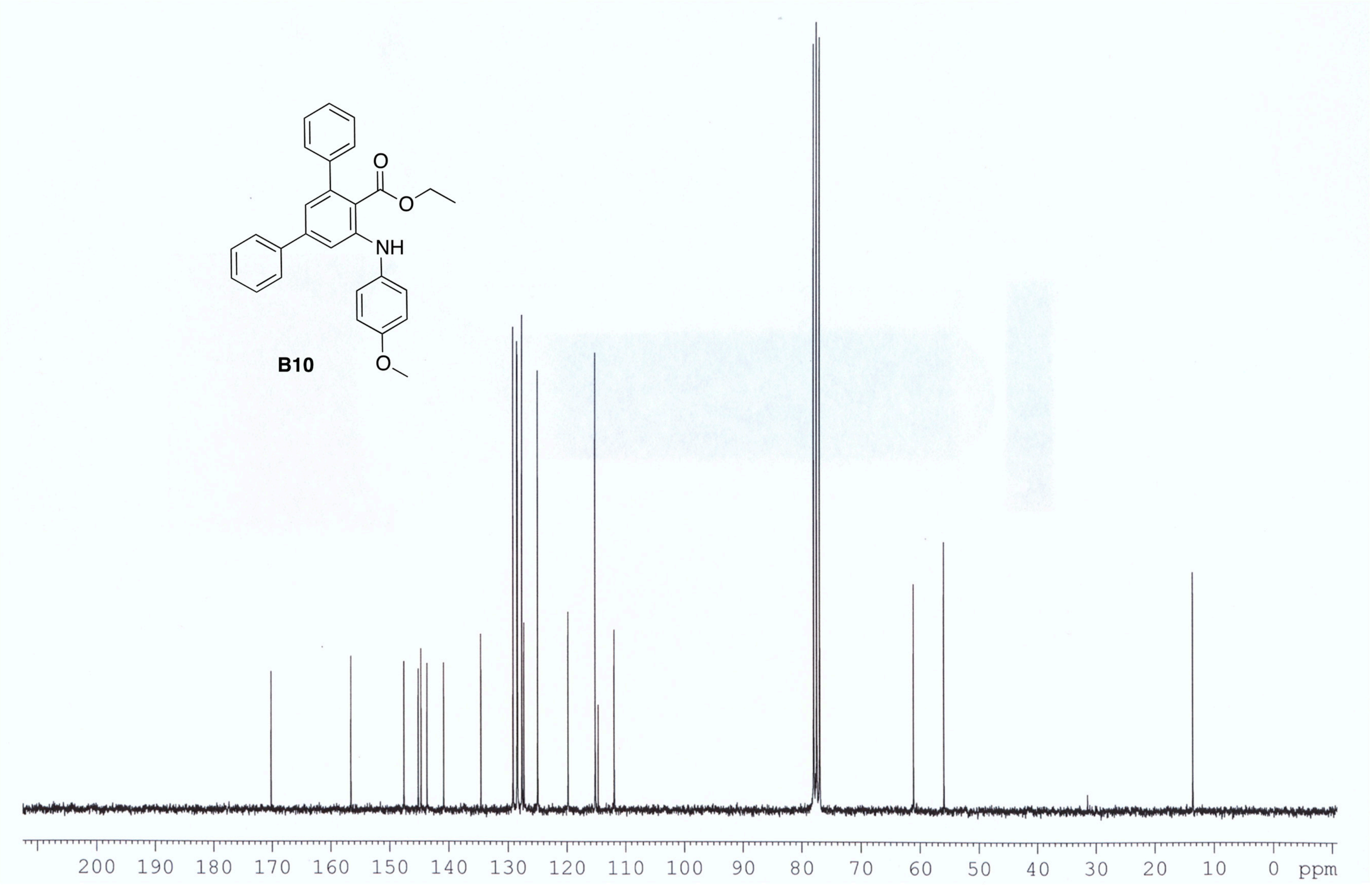

O
O
NH
O
B10
200 190 180 170 160 150 140 130 120 110 100 90 80 70 60 50 40 30 20 10 0 ppm

2. Spectroscopic study of compounds B

Table 1. UV-Visible absorption properties of compounds **B** [a]

Comp.	*Cyclohexane*		*Ethanol*		*Acetonitrile*	
	**λ_{max}, nm**	**ε, M^{-1} cm^{-1} (log ε)**	**λ_{max}, nm**	**ε, M^{-1} cm^{-1} (log ε)**	**λ_{max}, nm**	**ε, M^{-1} cm^{-1} (log ε)**
**B1**	244	36100 (4.56)	244	40100 (4.60)	244	38400 (4.58)
	367	4100 (3.61)	364	4900 (3.69)	364	3700 (3.57)
**B2**	253	44000 (4.64)	256	57000 (4.76)	253	47900 (4.68)
	368	4800 (3.68)	371	6000 (3.78)	367	5800 (3.76)
**B3**	248	32700 (4.51)	250	46100 (4.66)	249	38600 (4.59)
	261	4100 (3.61)	364	4100 (3.61)	361	4000 (3.60)
**B4**	255	46000 (4.66)	252	43400 (4.64)	254	47800 (4.68)
	363	4800 (3.68)	362	4300 (3.63)	364	4500 (3.65)
**B5**	250	35000 (4.54)	247	46000 (4.66)	247	42000 (4.62)
	369	2800 (3.45)	364	4500 (3.65)	364	4100 (3.61)
**B6**	245	47000 (4.67)	245	45000 (4.65)	245	54000 (4.73)
	362	3100 (3.49)	359	3700 (3.57)	359	4000 (3.60)
**B7**	242	41200 (4.61)	245	48000 (4.68)	245	38000 (4.58)
	362	3600 (3.56)	359	3500 (3.54)	359	3900 (3.59)
	244	13400 (4.13)	244	42500 (4.63)	244	19200 (4.28)
**B8**	271	11800 (4.07)	271	34500 (4.54)	274	15400 (4.19)
	364	3300 (3.52)	361	4300 (3.63)	358	3100 (3.49)
	246	34200 (4.53)	246	53900 (4.73)	247	36800 (4.57)
**B9**	273	27100 (4.43)	274	47400 (4.68)	271	28700 (4.46)
	364	5100 (3.71)	364	4300 (3.63)	362	4700 (3.67)
	246	58900 (4.77)	246	54100 (4.73)	247	49900 (4.70)
**B10**	273	47100 (4.67)	270	33000 (4.52)	270	37000 (4.57)
	364	4100 (3.61)	366	3700 (3.57)	362	3800 (3.58)

[a] λ_{max}: maximum absorption wavelength; ε: molar absorptivity (M^{-1} cm^{-1}) at the maximum absorption wavelength

Table 2. Fluorescence properties of compounds **B**

Comp.	*Cyclohexane*			*Ethanol*			*Acetonitrile*		
	λ_{ex}, nm	λ_{em}, nm	Φ_F	λ_{ex}, nm	λ_{em}, nm	Φ_F	λ_{ex}, nm	λ_{em}, nm	Φ_F
**B1**	259, 366*	457	0.13	257, 364*	453	0.29	247, 364*	459	0.24
**B2**	265, 366*	443	0.24	260, 368*	455	0.10	259, 367*	457	0.10
**B3**	260, 365*	444	0.25	260, 365*	454	0.29	258, 365*	455	0.27
**B4**	260, 364*	444	0.21	256, 364*	454	0.28	258, 364*	455	0.23
**B5**	257, 366*	442	0.15	256, 364*	450	0.29	256, 364*	453	0.11
**B6**	259, 362*	437	0.22	261, 362*	449	0.42	257, 360*	450	0.25
**B7**	260, 362*	437	0.24	261, 359*	446	0.38	256, 360*	448	0.21
**B8**	260, 365*	442	0.019	262, 362*	472	0.005	262, 361*	468	0.004
**B9**	272, 357*	462	0.002	278, 364*	494	0.002	274, 364*	494	0.001
**B10**	273, 362*	483	0.0025	275, 368*	511	0.001	275, 362*	532	0.0007

[a] λ_{ex}: maximum fluorescence excitation wavelength; λ_{em}: maximum fluorescence emission wavelength; Φ_F: fluorescence quantum yield; * fluorescence excitation wavelength employed for obtaining the emission spectra.

3. Analytical reactions with compounds A6 and A8

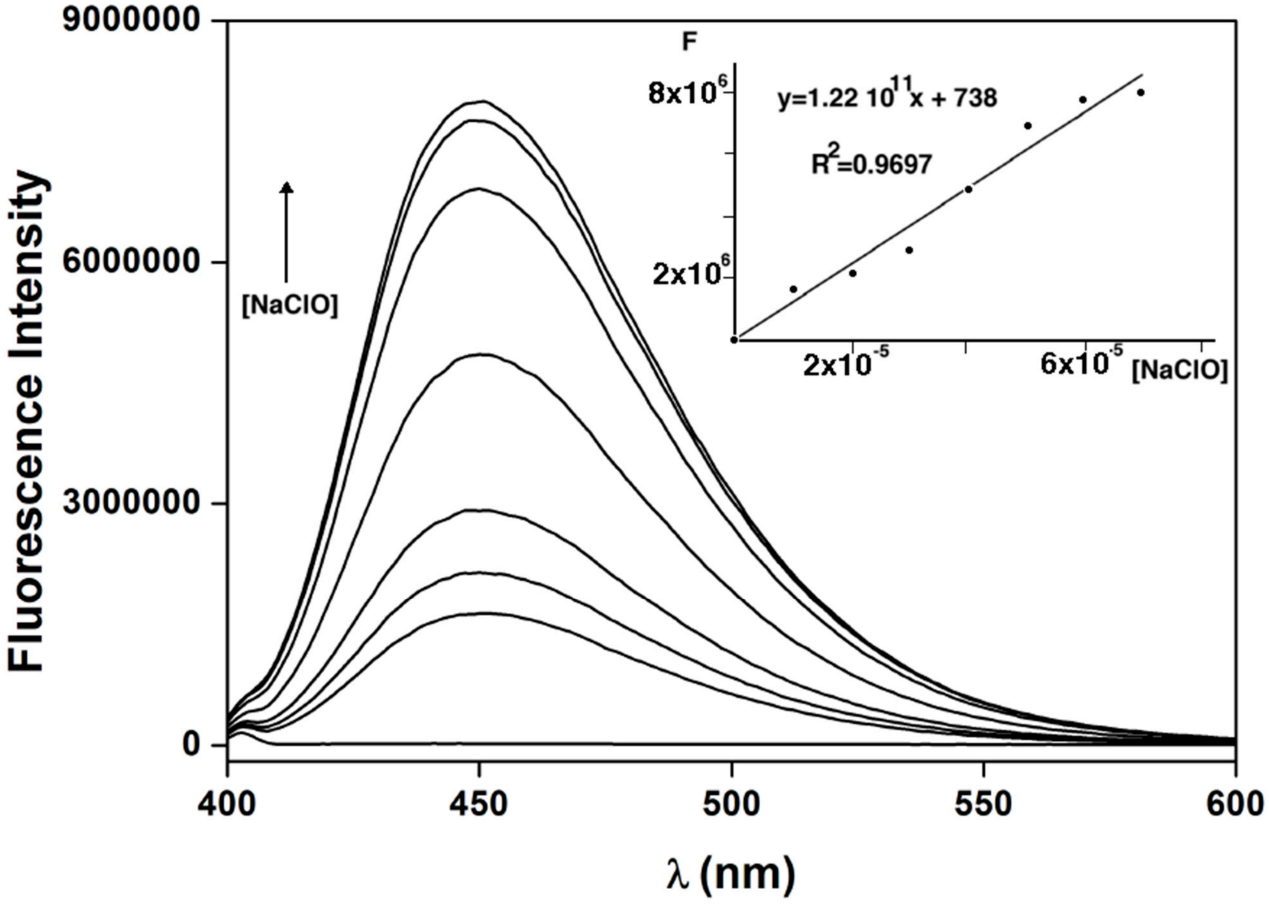

Figure S.1 Fluorescence emission spectra (λ_{ex} 360 nm) of the product obtained from the reaction of **A6** with increasing amounts of sodium hypochlorite.

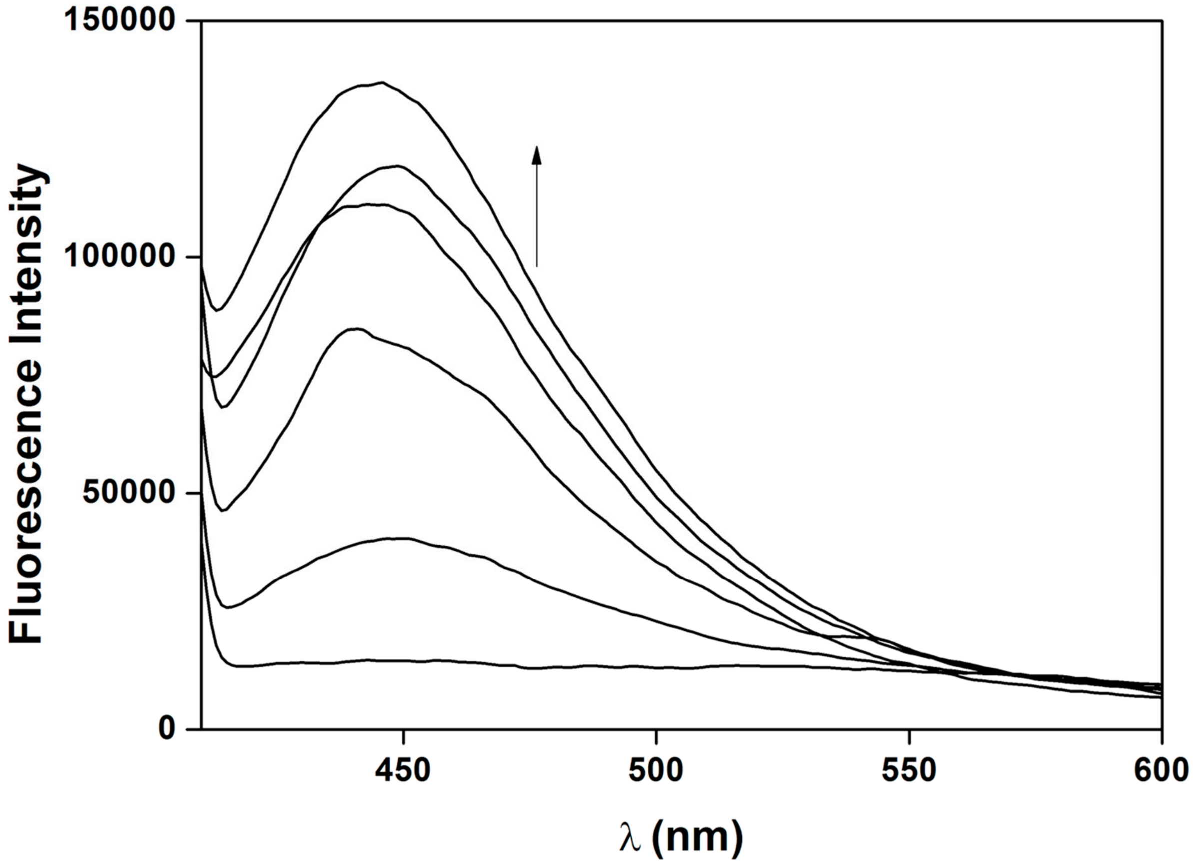

Figure S.2 Fluorescence emission spectra (λ_{ex} 360 nm) of the product obtained from the reaction of **A6** with increasing amounts of hydrogen peroxide.

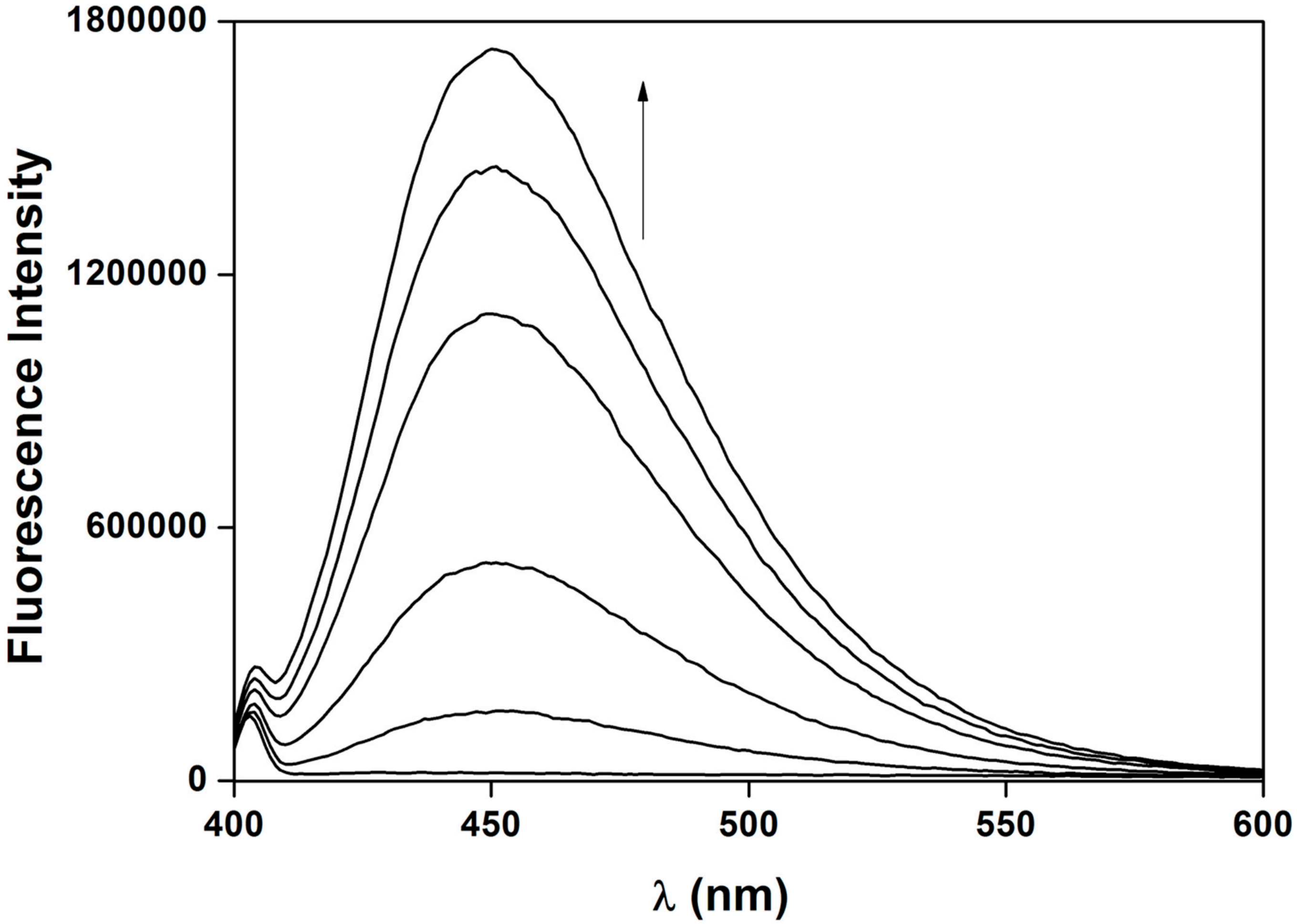

Figure S.3 Fluorescence emission spectra (λ_{ex} 360 nm) of the product obtained from the reaction of **A6** with increasing amounts of *tert*-butyl hydroperoxide

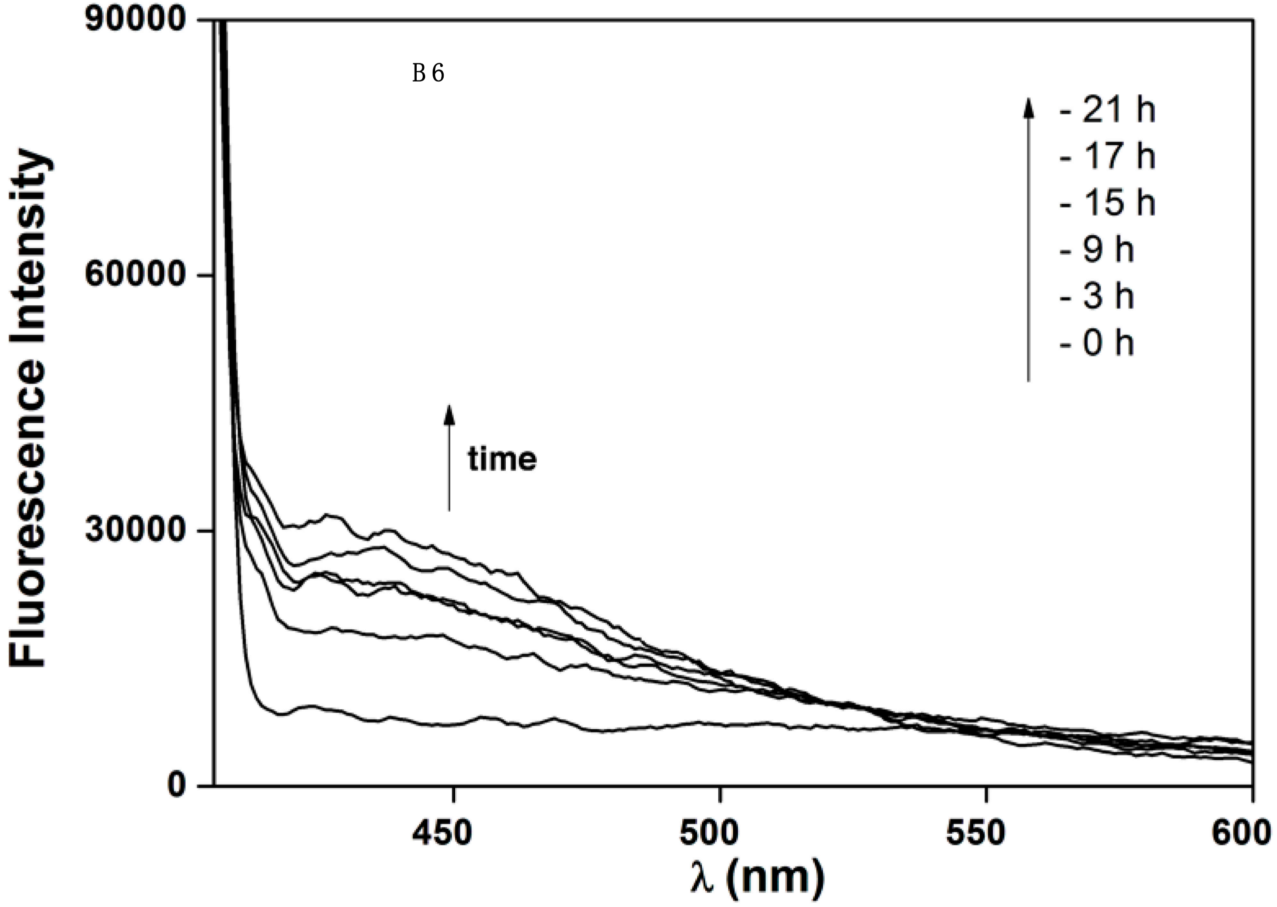

Figure S.4 Fluorescence emission spectra (λ_{ex} 361 nm) of the product obtained from the reaction of compound **A8** with the maximum amount of sodium hypochlorite employed for all other cases, after increasing the reaction time up to 21 h.
